# Metabolic symbiosis between oxygenated and hypoxic tumour cells: An agent-based modelling study

**DOI:** 10.1371/journal.pcbi.1011944

**Published:** 2024-03-15

**Authors:** Pahala Gedara Jayathilake, Pedro Victori, Clara E. Pavillet, Chang Heon Lee, Dimitrios Voukantsis, Ana Miar, Anjali Arora, Adrian L. Harris, Karl J. Morten, Francesca M. Buffa

**Affiliations:** 1 Department of Oncology, Medical Sciences Division, University of Oxford, Oxford, United Kingdom; 2 MRC Weatherall Institute of Molecular Medicine, Radcliffe Department of Medicine, Medical Sciences Division, University of Oxford, Oxford, United Kingdom; 3 Department of Computing Sciences and Institute for Data Science and Analytics, Bocconi University, Milan, Italy; 4 Nuffield Department of Women’s and Reproductive Health, University of Oxford, Oxford, United Kingdom; University of Southern California, UNITED STATES

## Abstract

Deregulated metabolism is one of the hallmarks of cancer. It is well-known that tumour cells tend to metabolize glucose via glycolysis even when oxygen is available and mitochondrial respiration is functional. However, the lower energy efficiency of aerobic glycolysis with respect to mitochondrial respiration makes this behaviour, namely the Warburg effect, counter-intuitive, although it has now been recognized as source of anabolic precursors. On the other hand, there is evidence that oxygenated tumour cells could be fuelled by exogenous lactate produced from glycolysis. We employed a multi-scale approach that integrates multi-agent modelling, diffusion-reaction, stoichiometric equations, and Boolean networks to study metabolic cooperation between hypoxic and oxygenated cells exposed to varying oxygen, nutrient, and inhibitor concentrations. The results show that the cooperation reduces the depletion of environmental glucose, resulting in an overall advantage of using aerobic glycolysis. In addition, the oxygen level was found to be decreased by symbiosis, promoting a further shift towards anaerobic glycolysis. However, the oxygenated and hypoxic populations may gradually reach quasi-equilibrium. A sensitivity analysis using Latin hypercube sampling and partial rank correlation shows that the symbiotic dynamics depends on properties of the specific cell such as the minimum glucose level needed for glycolysis. Our results suggest that strategies that block glucose transporters may be more effective to reduce tumour growth than those blocking lactate intake transporters.

## Author summary

Metabolic alteration is one of the hallmarks of cancer and the well-known metabolic alteration of tumour cells is that cells prefer to do glycolysis over mitochondrial respiration even under well-oxygenated and functional mitochondrial conditions. On the other hand, there is evidence that oxygenated tumour cells could be fuelled by exogenous lactate produced from hypoxic glycolytic cells in which it can create a metabolic co-operation between oxygenated and hypoxic cell populations. This metabolic co-operation could allow tumour cells to economically share oxygen and glucose and promote tumour survival. Using a multi-scale approach combining multi-agent modelling, diffusion-reaction, stoichiometric equations, and Boolean networks representing cell regulatory mechanisms, we studied this metabolic co-operation between different populations of cells, exposed to a changing microenvironment. We predict that the tumour environmental glucose depletion is decreased while the oxygen depletion is increased by this metabolic symbiosis, promoting a further shift towards glycolysis. Our results also show that blocking glucose transporters could be more effective than blocking lactate intake transporters, because the former would disrupt both glycolysis and lactate production, drastically reducing tumour growth.

## 1. Introduction

**Fig 1:**
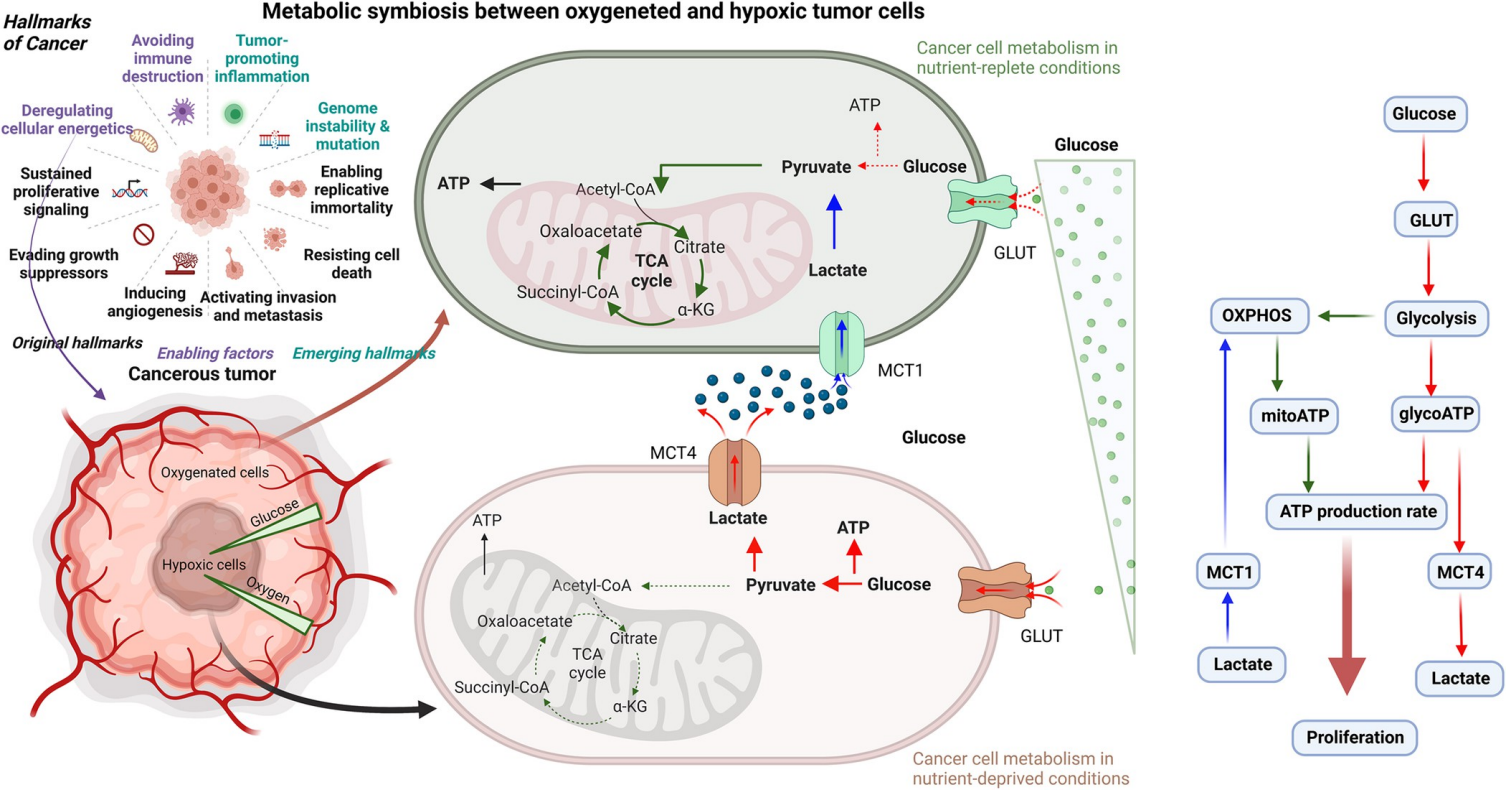
Metabolic symbiosis in tumour. Schematic diagram and flow chart illustrating the metabolic symbiosis mechanism between oxygenated and hypoxic tumour cells. Oxygenated cells at the tumour boundary (shown as green cells) consume exogenous lactate via MCT1 transporters and undergo lactate metabolism through OXPHOS. Inner hypoxic cells (shown as brown cells) consume glucose through GLUT transporters and undergo glycolysis and release lactate into the tumour microenvironment using MCT4 transporters. Metabolic symbiosis between the two cell populations (green cells and brown cells) helps hypoxic cells increase their glucose uptake, thereby helping tumour cells survive under low glucose conditions (created with BioRender.com).

Emergence of metabolic pathways played a vital role in cell evolution [1].Among metabolic pathways, glucose metabolism is one of the basic survival metabolic pathways of human cells. Glucose is imported from the extra-cellular environment through glucose transporters (GLUT) of which GLUT1, GLUT2, GLUT3 and GLUT4 are best characterized. In the presence of oxygen, healthy cells usually convert glucose into pyruvate and then pyruvate is converted to acetyl-CoA. The acetyl-CoA is oxidized in the mitochondria in the tricarboxylic acid (TCA) cycle [2]. This aerobic respiration or oxidative phosphorylation (OXPHOS) can produce about 28–36 ATP molecules per glucose molecule [3,4]. Under hypoxic/anoxic conditions (lack or absence of oxygen), as it often occurs in cancer, cells are not able to produce mitochondrial ATP and instead, they may use glycolytic ATP production. This only yields two ATP molecules per every glucose and lactate molecules. However, this respiration pathway is also used in presence of oxygen [3]. This use of glycolysis in aerobic conditions is known as the Warburg effect and it is one of the hallmarks of cancer cells [5]. The pyruvate produced by glycolysis is converted to lactate and then lactate and protons (H+ ion) are exported to the extra-cellular environment through membrane proteins called monocarboxylate transporters (MCT) which help to maintain the alkaline pH level inside tumour cells [6]. Glycolysis is an inefficient way to produce ATP and therefore more glucose is needed to maintain a sufficient ATP production rate for cell proliferation. When there is a low glucose concentration in the medium, cancer cells use lactate instead of glucose as their energy source [6,7]. Lactate is imported through MCT transporters (in addition to their function as lactate exporters) and the imported lactate is then converted back to pyruvate that can be oxidized in the mitochondria producing far more ATP molecules. This is the so-called “reverse Warburg effect”, one of the adaptive metabolic mechanisms of tumour cells [8,9]. Vascular tumours tend to have oxygenated cells in the tumour boundary, near vessels, and hypoxic cells in distant regions, thus this metabolic reprogramming could induce a metabolic symbiosis between lactate-fuelled oxygenated OXPHOS cells and glucose-fuelled hypoxic glycolytic cells [6,10], which, in turn, could result in a beneficial metabolic cooperation [7,10–14] (Fig 1). Therefore, inhibition of lactate consumption by cancer cells could be an effective therapeutic strategy [10,15]. On the other hand, lactate accumulation could cause higher intra-cellular acidity and lactate can also inhibit pyruvate dehydrogenase (PDH) and activate HIF.

The study of intra-cellular dynamics in the context of metabolic symbiosis of cancer presents a difficult challenge, as experimental approaches alone often fall short of capturing the dynamic and complex nature of these processes. To address these challenges, we employed computational modelling to simulate metabolic symbiosis of cancer. However, to gain a deeper understanding of the complex intra-cellular mechanisms underlying metabolic symbiosis in tumours, we needed a modelling framework suitable to study the interactions between heterogeneous cell populations, including the effect of perturbing intra-cellular gene networks (i.e., cell regulatory networks) and the cellular microenvironment. Thus, we developed a cell regulatory Boolean network built on prior knowledge and integrated it with a multi-scale computational model. Use of a multi-scale model was necessary to capture the effects of gene alterations on emerging properties of tumour and its microenvironment. Our model has three different spatial scales to describe different biological processes and cell-cell and cell-microenvironment interactions. The three scales are the intra-cellular, cellular, and extra-cellular scales and these scales are inter-connected each other. Both intra-cellular and cellular scales were modelled as agent-based models while the extra-cellular scale was modelled as a continuum model. More details about our model are given below. We investigated the possible beneficial effects of this potential synergy on the growth of the whole tumour and what factors influence the metabolic cooperation. Specifically, we asked:

- How would the metabolic cooperation between hypoxic and oxygenated cancer cells affect nutrient levels in the microenvironment?
- Does this metabolic cooperation lead to increased tumour growth, overall and for specific cell populations?
- How is this cooperation affected by changes in environmental conditions (levels of oxygen, glucose, lactate etc.), cell spatial competition, heterogeneous somatic mutations, and continuous fluctuation versus steady state of nutrient levels in the microenvironment?
- What are the best strategies to disrupt this metabolic symbiosis?

## 2. Methods: Multi-scale approach with multi-agent modelling, diffusion-reaction and stoichiometric equations, and Boolean network integration

**Fig 2:**
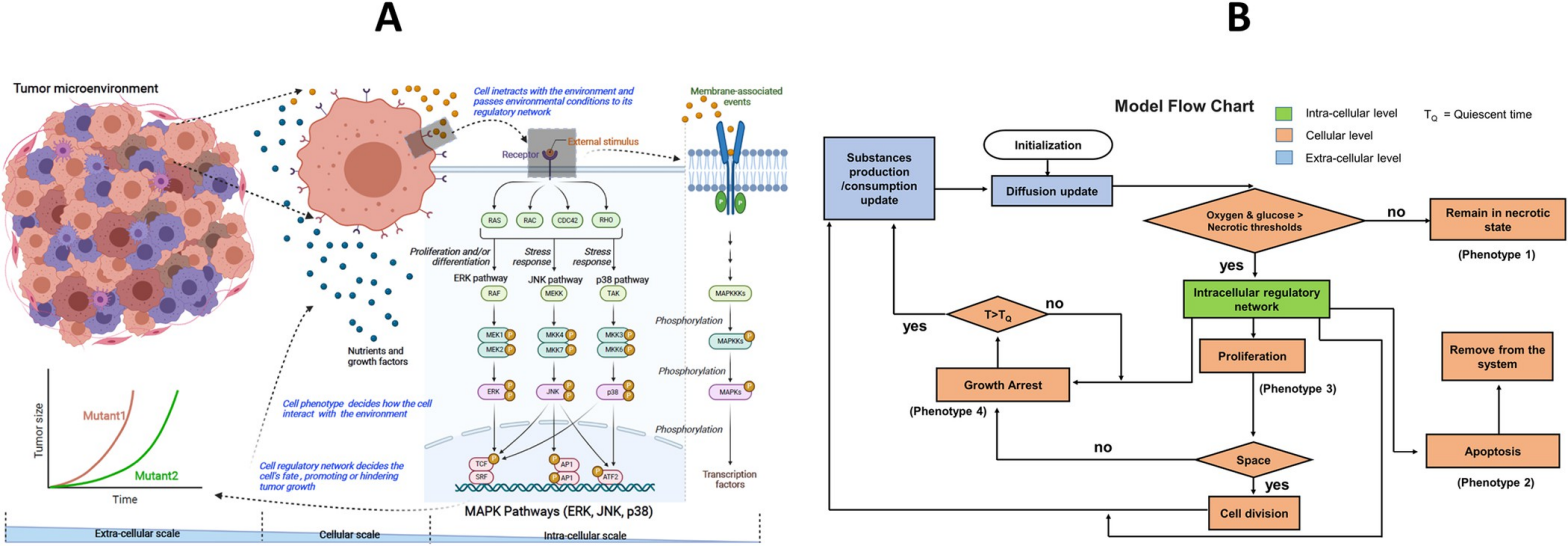
Agent-based mathematical model. **(A)**. The model is a multi-scale agent-based model implemented using the NetLogo platform. The extra-cellular scale models gradients of substances such as oxygen, glucose and lactate by using partial differential equations. The cellular scale models cell-cell interactions using a cellular automaton approach. The smallest scale, the intra-cellular scale, handles subcellular molecular interactions using a Boolean gene regulatory network. All scales communicate with each other and therefore the tumour growth is an emerging property of the sub-cellular molecular interactions. The model can be used to study tumour growth under different environmental conditions, gene alterations and heterogeneous cell populations (created with BioRender.com). **(B).** The model flow chart shows how the three different scales are connected. If the local oxygen and glucose levels are below their respective threshold values, the cell becomes necrotic. If the cell is not in the necrotic state, the intra-cellular regulatory network determines the cell fate which is Proliferation or Apoptosis or Growth Arrest. An apoptotic cell is removed from the simulation immediately. A proliferative cell can divide if there is empty space nearby, otherwise it can switch to growth arrest state and waits a T_Q_ time before checking environmental conditions again to find its new phenotype. The cell phenotype can influence the gradients of the diffusible substances on the microenvironment through producing/consuming diffusible substances based on its phenotype. The altered environmental properties are fed to the intra-cellular network of the cell again through input nodes and then the network can decide its new phenotype according to altered microenvironmental conditions.

Agent-based modelling (ABM) is a useful methodology to study ecological problems such as the one considered here and identify emerging behaviours of heterogeneous populations [16–20]. Indeed, ABM models have been employed by us and others to study how tumours respond to drugs [19,21–23], the impact of environmental conditions [22,24], cell metabolism [3,4,25,26] and cell competition [27,28]. However, most of these models have assumed homogeneous environments and populations and have none or extremely simplified sub-cellular molecular interactions. To investigate emerging behaviours from heterogeneous utilization of cellular pathways, we adopted a recently proposed multi-scale agent-based framework that enabled us to model cancer cells, the microenvironment surrounding them (including nutrients and oxygen), and their respective gene regulatory networks [16]. The model was developed building on the widely adopted modelling platform NetLogo [29], with previous [16] and new gene network and spatial functionalities developed by our laboratory as described below. Fig 2A shows the basic components of our model, and Fig 2B demonstrates how different spatial scales communicate with each other. More detailed descriptions of the model are given in S1 Text. The intra-cellular scale represents the cell’s gene regulatory mechanisms, which is a mitogen-activated protein kinase (MAPK) network [30], driving the growth of cancer cells (S2 Fig). Additionally, we modelled metabolic pathways including cellular respiration, glucose and lactate metabolism, and resulting ATP production. Specifically, glucose is imported through GLUT1 transporters (Note that not all GLUT isoforms are included here and GLUT1 is highly expressed in breast cancer samples as shown in S4 Fig) and is converted to pyruvate at the end of the glycolysis process (Figs 1 and S2). The pyruvate can go through OXPHOS, which produces mitochondrial ATP (mitoATP) or can be converted into lactate resulting in only glycolytic ATP (glycoATP). Both mitoATP and glycoATP will determine the ATP production rate, and if the ATP production rate is above a certain threshold, that is 80% of the maximum possible ATP production rate [4], the cell will be able to proliferate–subject to inhibition by other parts of the regulatory network. When cells do not use mitochondrial respiration, the pyruvate will be converted to lactate and then it will be exported to the environment through MCT4 membrane proteins. When there is enough lactic acid and oxygen in the medium, cells will import lactic acid through MCT1 membrane proteins. The lactate will be converted back into pyruvate and pyruvate will go through OXPHOS producing mitochondrial ATP [12,31,32]. To cover cellular functions including proliferation, apoptosis, growth arrest, cellular response to growth factors and the hypoxic microenvironment, we modelled the links between glucose/lactate metabolism and a previously developed MAPK-HIF Boolean network [16,30]. Each node of the Boolean network has its own specific Boolean logical condition and if the condition is true the node is considered as active (i.e., 1) and otherwise inactive (i.e., 0). To model Boolean logical conditions for the metabolic network nodes and the nodes linking with the existing MAPK-HIF network, supporting evidence was used from the literature (Table A in S1 Text). This network is encapsulated inside each tumour cell and the network can obtain stimuli such as growth factors and nutrients from the extra-cellular environment through input nodes which represent membrane receptors and transport gates. Our choice of a Boolean network to represent intra-cellular molecular interactions has been discussed previously [16]. Briefly, it was motivated by its ability to capture the essence of complex regulatory networks in a simplified yet biologically meaningful and computationally efficient manner. Boolean networks allowed us to model cancer cell behaviours through binary on/off states of its genes, effectively lumping complex intra-cellular molecular interactions into manageable, logical conditions. In this study, we leveraged the inherent simplicity and flexibility of Boolean networks over other computational methods such as ordinary differential equations to gain insights into the regulatory processes that drive metabolic symbiosis within tumour cells.

In the cellular scale, each cell is represented by entities in a lattice. Gradients of diffusible substances in the microenvironment (glucose, oxygen, lactate etc.) are modelled in the extra-cellular scale. The soluble substances: oxygen, glucose, lactate and growth factors (GFs), are described by the diffusion-reaction equation as given below.

$$\frac{\partial C_{S}}{\partial t}=D_{S}\nabla^{2}C_{S}+R_{S}\rho_{cell,S}$$ (1)

where *C*, *D* and *R* are the substance concentration, its diffusion coefficient and its rate of consumption or production, respectively. *ρ*_*cell*,*s*_ represents the density of cells which consume or produce substance *S*. Typically, the time scale of substance diffusion (order of seconds) is much smaller than that of cell phenotype changes (order of hours) and therefore the diffusion is assumed to be in a steady state when the cell phenotype is updated. Cell necrosis occurs when both oxygen and glucose levels are lower than their respective critical values, as previously used [20]. If proliferative cells do not have enough neighbouring space to divide, those cells will switch to a quiescent state until they have some empty space to proliferate, thus modelling contact inhibition [33,34]. We used the model in two-dimensional space (2D) for the present study.

The sink term of Eq (1) (R) is described in accordance with the stoichiometry of oxygen, glucose, and lactate in their respective reaction equations [3,4,27]. Therefore, the oxygen consumption rate *R*_*o*2_ is modelled as $R_{o2}=\mu_{o2}\frac{C_{o2}}{K_{o2}+C_{o2}}(mitoATP+K\cdot glycoATP),K=0.5$, in which *μ*_*o*2_ is the maximum oxygen consumption rate and *K*_*o*2_ is the half-saturation coefficient. mitoATP and glycoATP are dynamic variables and their status are provided by the executable cell regulatory network encapsulated inside each cell (S1 and S2 Figs), at each time step of the simulation. Specifically, when the cell uses glycolytic ATP production, glycoATP is 1 and otherwise it is 0. Similarly, if the cell uses mitochondrial ATP production, then mitoATP is 1 and otherwise it is 0. The value of *K*·glycoATP reflects the amount of oxygen consumption by a cell engaged in the glycolysis process. Here, we assume that even under glycolysis the tumour cells can consume a certain amount of oxygen for some other cellular processes, such as for example macromolecule synthesis [35].

The glucose consumption rate can be calculated using the stoichiometry of the glucose oxidation equation given below.

$$C_{6}H_{12}O_{6}+6O_{2}\to {6CO}_{2}+{6H}_{2}O+A_{0}\cdot ATP$$ (2)

where, *A*_0_ is the ATP yield at the relevant oxygen and glucose abundance conditions, which is about 28 to 36 ATP molecules. The proportion of ATP production by glycolysis and OXPHOS depends on the cell type [36] and therefore, we assume that glycolytic and OXPHOS cells have similar ATP production rates for their proper functioning. The glucose consumption rate is then modelled as

$$R_{G}=\frac{\mu_{o2}}{6}\frac{C_{o2}}{K_{o2}+C_{o2}}\frac{C_{G}}{K_{G}+C_{G}}(mitoATP)+\frac{\mu_{o2}}{6}\frac{A_{0}}{2}\frac{C_{G}}{K_{G}+C_{G}}(glycoATP).$$ (3)

Taking different ATP production efficiencies of OXPHOS and glycolysis, the ATP production rate of a cell is then described as

$$R_{ATP}=A_{0}\frac{\mu_{o2}}{6}\frac{C_{o2}}{K_{o2}+C_{o2}}\frac{C_{G}}{K_{G}+C_{G}}(mitoATP)+A_{0}\frac{\mu_{o2}}{6}\frac{C_{G}}{K_{G}+C_{G}}(glycoATP).$$ (4)

The glucose conversion into pyruvate in the glycolysis process can be written as

$$C_{6}H_{12}O_{6}+{2NAD}^{+}+2{HPO}_{4}^{2-}+2ADP\to 2C_{3}H_{3}O_{3}+2NADH+2ATP+2H_{2}O+2H^{+}$$ (5)

and then pyruvate conversion to lactate can be written as

$$C_{3}H_{3}O_{3}+NADH+H^{+}\to C_{3}H_{6}O_{3}+{NAD}^{+}.$$ (6)

Eqs (5) and (6) show that one glucose molecule can produce two lactate molecules through the Warburg effect, and therefore the lactate production rate can be modelled as

$$R_{L,p}=\frac{{2\mu }_{o2}}{6}\frac{A_{0}}{2}\frac{C_{G}}{K_{G}+C_{G}}(glycoATP).$$ (7)

Considering that stoichiometry between glucose and oxygen is 1:6 for mitochondrial respiration (Eq 2), and glucose to pyruvate is 1:2 for glycolysis, it is assumed that lactate to oxygen ratio for reverse Warburg effect is 1:3. Therefore, the lactate consumption rate for the reverse Warburg effect is estimated as

$$R_{L,c}=\frac{{2\mu }_{o2}}{6}\frac{C_{o2}}{K_{o2}+C_{o2}}\frac{C_{L}}{K_{L}+C_{L}}(mitoATP).$$ (8)

The glycolysis pathway can produce two protons (H+) per glucose molecule and hence the proton production rate is modelled as

$$R_{H+}={\beta A}_{0}\frac{\mu_{o2}}{6}\frac{C_{G}}{K_{G}+C_{G}}(glycoATP).$$ (9)

Here, *β*<1 is the proton buffering coefficient of the tumour microenvironment **[4]** and it is chosen as 0.001 for this model because it results in a realistic pH level in the tumour microenvironment. The local extra-cellular pH due to exported H+ ion is calculated as *pH* = −*log*10([*H*+]).

The consumption rate of growth factors and inhibitors are modelled as *R*_*S*_ = *γ*_*S*,*C*_*C*_*S*_ and the production rate of any growth factor is described as *R*_*S*_ = *γ*_*S*,*P*_.

The diffusible substance (oxygen, glucose, growth factors etc.) concentrations are kept constant at the boundary of the computational domain. These boundary values and all other model parameters are given in Table C in S1 Text. Not all the parameters are based on evidence from previous studies, and therefore some parameter values are assumed based on other relevant data. However, we ran ten replicates of each simulation to assess the effect of different initial conditions, and a sensitivity analysis based on Latin hypercube sampling and partial rank correlation [37,38] was performed to ensure the correctness and robustness of all our conclusions.

The level of symbiosis is quantified by the metabolic symbiosis index (*MSI*) and its definition is motivated by [31] as below:

$MSI=\frac{\emptyset_{L}(A_{oxy})}{\emptyset_{L}(A_{oxy})+\emptyset_{G}(A_{oxy})}‐\frac{\emptyset_{G}(A_{oxy})}{\emptyset_{L}(A_{oxy})+\emptyset_{G}(A_{oxy})}$ if ∅_*L*_(*A*_*oxy*_)>∅_*G*_(*A*_*oxy*_) and ∅_*L*_(*A*_*hypo*_) < ∅_*G*_(*A*_*hypo*_); and *MSI* = 0 otherwise. Here, ∅_*L*_(*A*) and ∅_*G*_(*A*) are lactate metabolic and glycolytic cell fractions of the tumour region A, respectively. *A*_*oxy*_ and *A*_*hypo*_ are oxygenated and hypoxic regions of the tumour, respectively. The symbiosis index would vary from 0 to 1 depending on the strength of the symbiosis.

## 3. Results & discussion

### 3.1 A gene network model of cellular metabolism and respiration

First, we used RNAseq data for multiple breast cancer cell lines grown under normoxic and hypoxic conditions (see S4 Fig for results, S1 Text for experimental conditions) to evaluate the coherence of our modelling assumptions. We observed that for cells under hypoxic conditions, the expression levels of SLC2A1 (or GLUT1), SLC16A3 (or MCT4) and LDHA increased, in agreement with our model of the Warburg effect and previous reports [39–41]. The increased expression of these genes supports our modelling hypothesis that hypoxic tumour cells transport more glucose, then to convert it into pyruvate, and finally the pyruvate would be metabolized to lactate and exported to the tumour microenvironment through MCT4 transporters as we have modelled in our Boolean network (S2 Fig).

**Fig 3:**
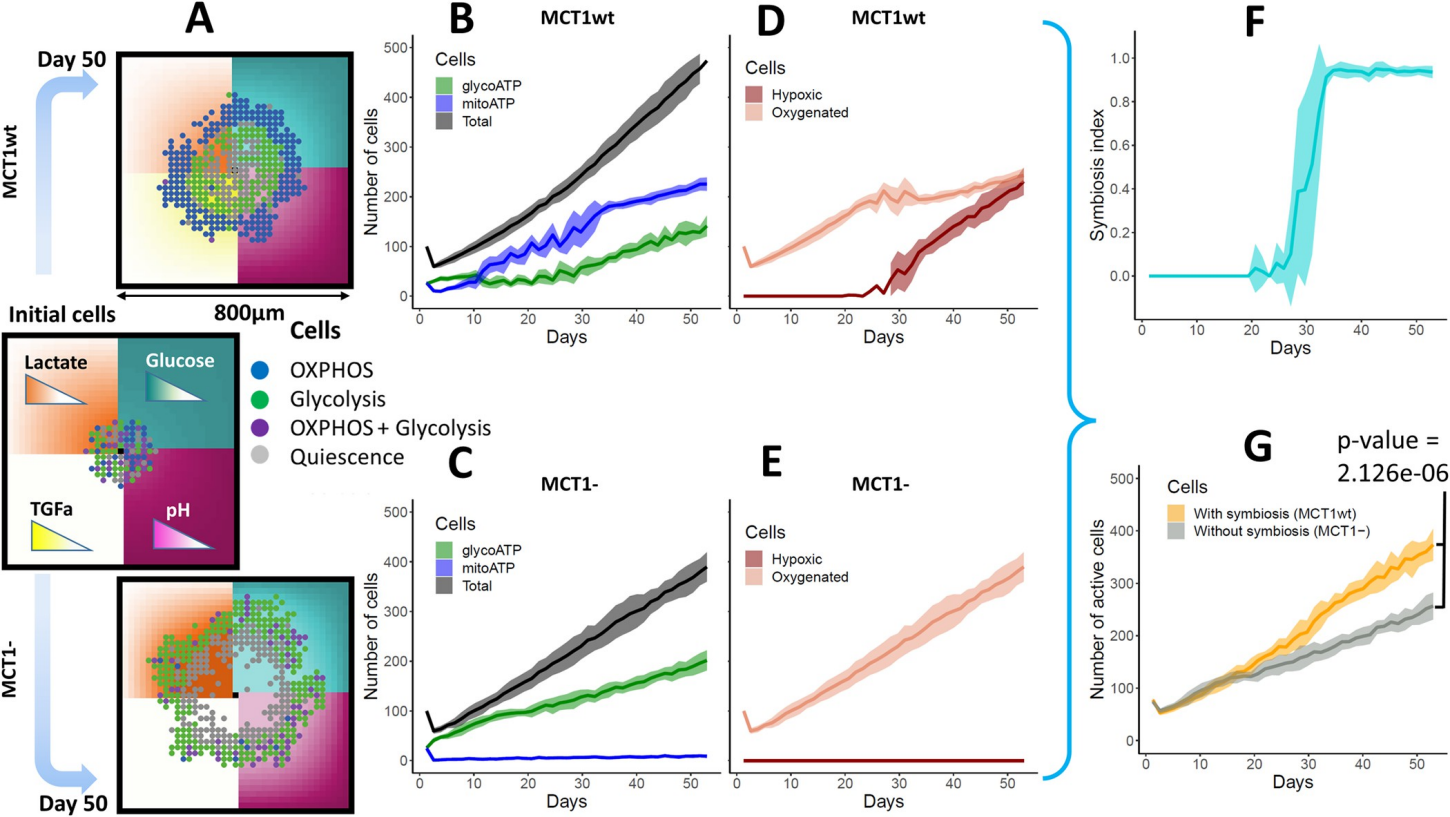
Tumour growth and symbiosis. **(A)**. If MCT1 is not mutated (MCT1wt), symbiosis is observed, while when MCT1 is mutated with loss of function (MCT1-), symbiosis is not observed. Different cell populations are shown: OXPHOS (blue cells, glucose/lactate—> pyruvate—>OXPHOS), Glycolysis (green cells, glucose—>pyruvate—>lactate), both OXPHOS and glycolysis (purple cells), not metabolically active or quiescence (gray cells). Gradients of glucose, lactate, tumour growth factor alpha (TGFA) and pH are shown on the microenvironment. **(B, C)**. Number of total, glycoATP and mitoATP cells are shown. The total number of cells contains all the cells in the tumour including dead cells. The mitoATP and glycoATP are the number of cells that rely on OXPHOS and glycolysis for ATP production, respectively. **(D, E)**. Number of hypoxic and oxygenated tumour cells with MCT1 wild type and mutated condition are shown. The hypoxic cell population is not seen when symbiosis is lost (MCT1-) because oxygen cannot be depleted fast enough without lactate oxidation. **(F, G)**. Metabolic symbiosis index and number of active (viable) cells are shown when tumour grows with MCT1wt and MCT1-, so with and without symbiosis. The metabolic symbiosis index quantifies the strength of symbiosis between hypoxic and oxygenated tumour cells. The index can range from 0 to 1 depending on the symbiosis strength. The active cells are the cells with their metabolic pathways are active (i.e., blue, green and purple cells shown in A). The shaded area of curves shows respective standard deviation.

We then used the model with parameters setting in Table C in S1 Text to investigate metabolic symbiosis between oxygenated and hypoxic cell populations. Our simulations showed metabolic symbiosis between hypoxic and oxygenated cells of the tumour when MCT1 was in wild type status (MCT1wt), while this symbiosis was lost when MCT1 was knocked out in our model. Of note, we model knockouts by setting a constrain on the status of the corresponding node in the Boolean network (MCT1) to inactive (i.e., 0 status), for the duration of the current simulation. When a tumour grows with MCT1wt, we observe symbiosis, whereby the well-oxygenated tumour cells on the boundary of the simulated organoid switch to OXPHOS while hypoxic inner cells switch to glycolysis (Fig 3A). Importantly, glycolytic and OXPHOS cell populations co-exist under symbiosis, while the tumour growth mainly depends on the aerobic glycolytic population when symbiosis stops (Fig 3B and 3C). In the symbiotic tumour, the size of both cell populations is initially comparable, and the OXPHOS cell population dominates over the glycolytic cell population later when the glycolytic cells produce more lactate for OXPHOS cells (Fig 3B). However, when MCT1 is inactive the symbiosis is lost and the OXPHOS cell population is about only 4.5% of the glycolytic cell population at the end of the simulation because OXPHOS is not driven by lactate (Fig 3C). When symbiosis exists in the tumour, we observe that the cells at the boundary consume more oxygen for lactate metabolism, and therefore oxygen is depleted inside the tumour pushing to a more hypoxic environment (Fig 3D). However, when symbiosis stops, the emergence of a hypoxic cell population is delayed (Fig 3E). As the hypoxic population emerges when symbiosis exists in the tumour, the symbiosis index rapidly increases from zero to 1 indicating that metabolic cooperation between oxygenated and hypoxic tumour cells establishes at that time (Fig 3F). These simulation results clearly show that a tumour would gain a growth advantage due to metabolic symbiosis because symbiotic tumour cells can proliferate through either glycolysis (i.e., glucose to lactate) or OXPHOS (i.e., glucose/lactate to OXPHOS), or both pathways (Fig 3G). In support of the results from our simulations, in-vitro experiments have also shown that lactate metabolism can increase growth of human cancer cells in glucose-limited mediums of breast [15], glioma [31] and colon [10] cancer. In-vitro studies of breast cancer cells have also shown that the presence of lactate would enable cells to withstand glucose-limited conditions [15].

### 3.2 Metabolic symbiosis between oxygenated and hypoxic tumour cells depends on the status of genes that are commonly altered in cancer

We then asked if a similar scenario would be observed under a number of gene alterations that are commonly observed in cancer. Therefore, we examined gene aberration and expression patterns observed in the clinical setting for breast cancer samples taken from The Cancer Genome Atlas (TCGA) (see S1 Text for details). We specifically asked how our network genes are likely to either over or under-express in clinical samples. We took a conservative approach and considered the gene as over-expressed when the standard deviation was above +3 with respect to normal samples and under-expressed as when it was below -3. S5 Fig shows the probability of aberrant gene expression (i.e., the percentage of samples that gene is over (S5A Fig) or under expressed (S5B Fig)). Of note, typically a greater number of genes would be considered as differentially expressed with respect to normal samples, but for the current study, we restricted the set of genes to the genes associated with our network. As can be seen in our results, in our network more genes tend to be under-expressed than over-expressed. Using a published protocol-sigQC [42] we also looked for a correlation among the absolute expression of these genes taken from TCGA (S6A Fig) and Cancer Cell Line Encyclopedia (CCLE) (S6B Fig) databases. Although some correlations could be observed, this was overall not strong with correlation coefficients found between -0.5 and +0.5, and many around 0. Thus, for the purpose of our simulations we assumed in first instance that the aberrant over- or under-expression of these genes occurred independently of each other (S6C Fig). While this might be improved in future studies, it allowed us to simplify the problem in first instance, and simulate each gene knockout or enrichment in our model as a single event rather than as linked coordinated events. Furthermore, TCGA and CCLE datasets (S6C Fig) showed similar distributions of sigQC metrics of variability and expression, suggesting that this gene set would be applicable to both cell lines and clinical samples. More details about these metrics can be found in [42] and are not repeated here.

**Fig 4:**
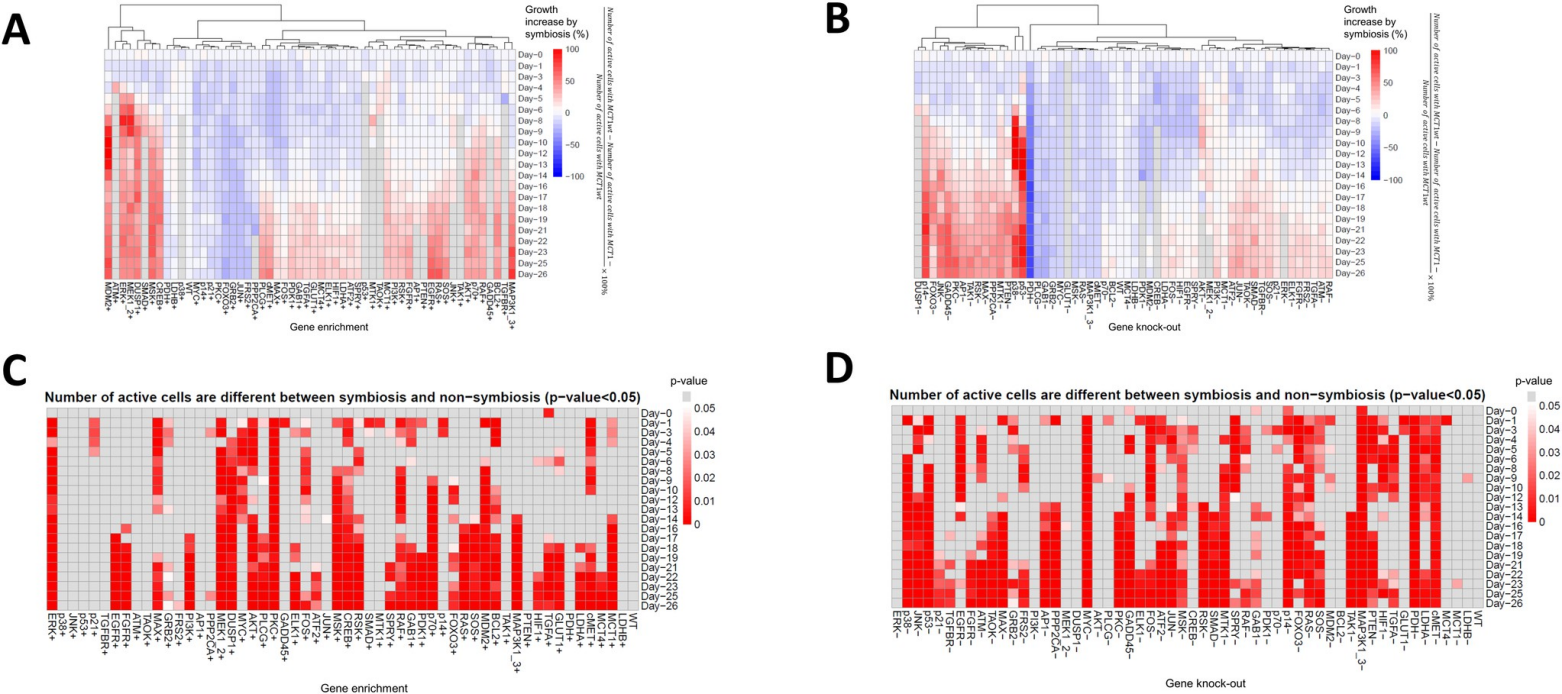
Metabolic symbiosis simulations with network gene alterations: The gene enriched (+) and knockout (-) status were simulated by setting the respective node of the regulatory network to 1 and 0, respectively. The gene wild type (WT) status was simulated without setting the respective node to either 0 or 1. Each gene was altered individually. **(A)**. Percentage tumour growth increase due to symbiosis is shown for each gene enrichment status. **(B)**. Percentage tumour growth increase due to symbiosis is shown for each gene knockout status. **(C)**. Whether tumour growth is significantly different (p-value < 0.05) between symbiosis and non-symbiosis for each gene enrichment status is shown. **(D)**. Whether tumour growth is significantly different (p-value < 0.05) between symbiosis and non-symbiosis for each gene knockout status is shown. Clusters of gene alterations can be identified, which enhance tumour growth due to symbiosis while some other gene alterations together with symbiosis adversely affect tumour growth (A, B). Colours indicate percentage growth increase by symbiosis (A, B) and p values (C, D). p values from 0 to 0.05 are shown in red to white colour scale and p values ≥ 0.05 are shown in grey colour.

Next, we perturbed the status of each of the network genes in our model and the model was run with MCT1wt and MCT1- conditions to investigate symbiosis and possible interaction between symbiosis and gene alterations commonly observed in cancer. To investigate the symbiotic relationships and potential interactions between gene alterations in cancer, we utilized our Boolean network for efficient simulation in our model. Each gene in the network was systematically perturbed to observe dynamic responses under different genetic conditions. The model was then run with MCT1wt and MCT1- conditions, allowing us to explore the interplay between gene alterations and symbiotic processes. The discrete nature of Boolean networks has provided us a straightforward approach to alter individual genes and study their interactions with metabolic symbiosis. This investigation can not only reveal individual effects but also discover potential synergies or antagonisms between altered genes and metabolic symbiosis. We asked whether each of these gene alterations would affect the metabolic symbiosis-induced growth of the tumour (Fig 4A and 4B), symbiosis index (S7A and S7B Fig) and if there was a difference between the number of active (viable) cells in symbiotic and non-symbiotic tumours (Figs 4C, 4D, and S7C–S7E). As we can see in Figs 4A, 4B, S7A, and S7B, some gene alterations give a growth advantage when there is symbiosis between hypoxic and normoxic cells (MDM2+, ERK+, MSK+, p38-, p53-, p14- etc.; here + and—indicate enriched and knockout status, respectively, in which the corresponding Boolean node is set to 1 and 0, respectively), while some other gene alterations reduce tumour growth under symbiosis (p14+, FOXO3+, GRB2+, RAS-, PLCG-, MYC- etc.). Symbiosis-induced growth advantage is seen at the later stage of tumour growth because it takes some time for the emergence of hypoxic cells in the tumour and then establish a cooperative interaction between two cell populations, approximately after 17 days (S7A and S7B Fig).

Gene enrichments such as EGFR+, ERK+ and DUSP1+ and gene knockouts such as p53-, p38-, PDH- significantly change the number of active cells obtained between symbiotic and non-symbiotic tumours (Figs 4C, 4D, and S7E). Similarly, gene enrichments such as MDM2+, ERK+ and DUSP1+ and gene knockouts such as p53-, p38-, p14- significantly increase the number of active cells obtained under symbiosis compared to non-symbiosis conditions (S7C–S7E Fig).

It is important to note that under MYC+ condition, the tumour growth is increased under both MCT1wt and MCT1- conditions. However, the tumour growth increase in the presence of symbiosis (MCT1wt) is slightly less than the tumour growth increase in the absence of symbiosis (MCT1-), and therefore the percentage change in tumour growth due to symbiosis is negative (i.e., decrease) as seen in Fig 4A. Interestingly, for some genes, including for example GRB2, MYC, and LDHB, the tumour growth in the presence of symbiosis is reduced regardless of the nature of gene alteration (enrichment or knockout). This is somewhat counterintuitive; however, it does not mean that tumour growth rate is exactly the same under both types of gene alterations. For example, GRB2- tumour cells grow faster than GRB2+ cells, though both alterations reduce tumour growth at similar proportions under symbiosis.

These results taken together introduce the important concept that the impact of symbiosis on the tumour, whether beneficial or detrimental, will also depend on the type of gene aberrations present in the tumour cells. Thus, this supports the need for more advanced genomics and metabolic combined classifications of patients, which consider not only the gene expression observed at a given time before treatment, but also the predicted interaction of that specific gene expression profile with a given perturbation. In the present model, knockout of MCT1 transporter blocks lactate consumption and hence metabolic symbiosis is disrupted, but these concepts could be extended to other transporters and receptors. As shown above, the application of Boolean gene networks proves highly valuable in understanding the impacts of such alterations in cell molecules on the macroscopic emergent characteristics of tumors.

### 3.3 p53 status affects the metabolic symbiosis between oxygenated and hypoxic cells

**Fig 5:**
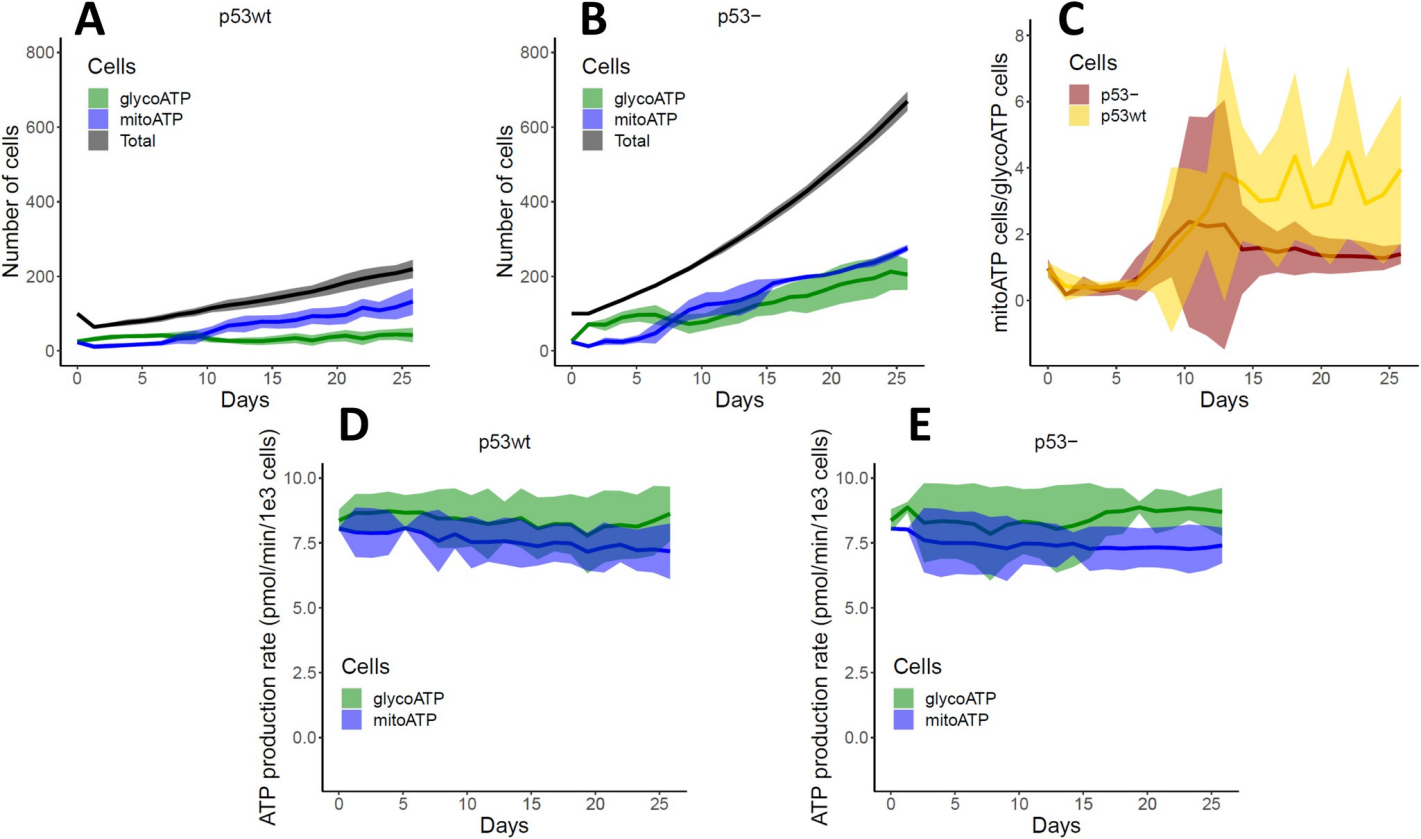
Tumour growth under wild type and mutated p53 status. Effect of p53 status on OXPHOS (shown as mitoATP), glycolysis (shown as glycoATP) and ATP production rate are shown. p53wt and p53- cells were grown in isolation. The starting number of cells was 100. **(A, B)**. Growth of p53wt (A) and p53- (B) cells over time. The OXPHOS cell population is marginally dominant over glycolytic population regardless of the p53 status. **(C)**. Evolution of OXPHOS/Glycolysis cell ratio with p53wt and p53- status. **(D, E)**. Variation of OXPHOS (mitoATP) and glycolytic (mitoATP) ATP production rate of p53wt (D) and p53- (E) cells as tumour grows. The tumour cells with p53 knockout were found to grow faster than p53wt cells because they lacked p53 to suppress tumour growth.

The most commonly mutated gene in cancer, and also in our samples, is the tumour suppressor p53. TP53 is mutated in 65.5% of the CCLE breast cancer samples and 32.6% of TCGA breast cancer samples (TCGA, PanCancer Atlas) [43]. Not only p53 is well-known to be involved in apoptosis and growth arrest of cells [44], but it is also a regulator of the glycolysis pathway with mutations in p53 deregulating cell metabolism in a number of ways including increased GLUT expression [45,46]. Therefore, we studied the effect of p53 alterations on metabolic symbiosis of oxygenated and hypoxic cells in cancer by growing p53wt and p53- cells in isolated populations in our model. Fig 5 shows variations of cell numbers, cell metabolic pathway utilization and ATP production rates for p53wt and p53 knockout (p53-) simulations. Tumour growth is predicted to be accelerated under p53-, which is expected because cell apoptosis is stopped. However, our simulation proposes an additional mechanism for this because in p53- cells, the glucose transporter GLUT1 expression is increased, and therefore the higher glucose uptake contributes to the higher growth rate. In our results, the number of cells producing mitoATP (i.e mitochondrial ATP production through OXPHOS) is greater than the number of cells producing glycoATP (i.e glycolytic ATP production through glycolysis alone). This is a consequence of lactic acid excretion by the glycolytic cells and its utilization by oxygenated OXPHOS tumour cells (Fig 5A and 5B). By comparing OXPHOS/Glycolysis ratio, it is clear that p53- enhances glycolysis over time, partly because inhibiting p53 causes increased glucose uptake as mentioned above (Fig 5C). As expected, the cell ATP production rate is found to be independent from both p53 status and metabolic pathway because it was assumed that cells can adjust their nutrient consumption rates to maintain a similar ATP production rate for all metabolic pathways (Fig 5D and 5E). For example, ATP production rates of glycolysis and OXPHOS are similar while glucose consumption by glycolytic cells is much higher than OXPHOS cells.

**Fig 6:**
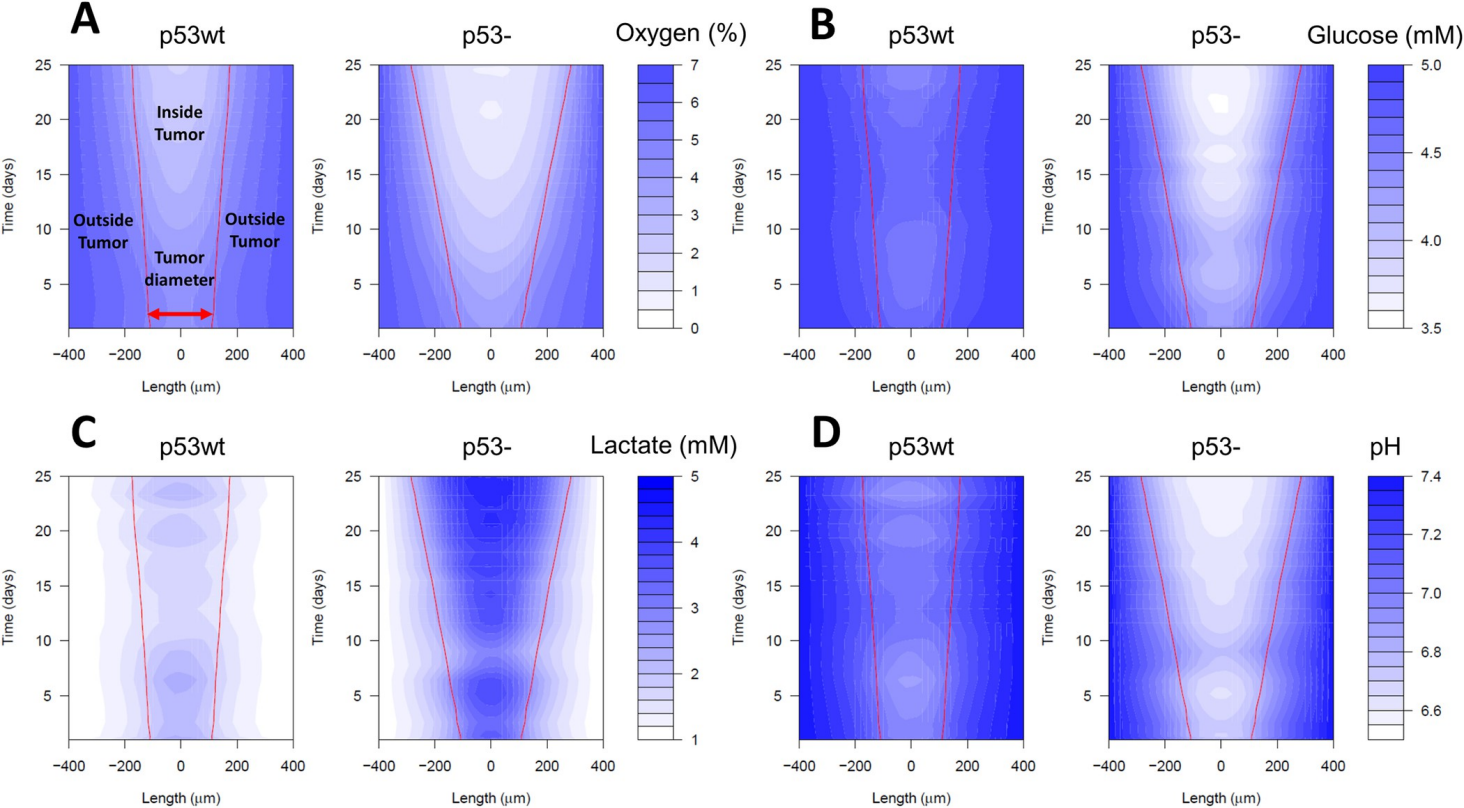
Distribution of tumour microenvironmental substances under wild type and mutated p53 status. (A-D). Heat maps show spatial-temporal variation of microenvironmental (A) oxygen, (B) glucose, (C) lactate, and (D) pH levels for p53wt and p53- tumour growth (here, the Length is the cross section of the tumour microenvironment through the center of the tumour). Red lines show the development of tumour boundary over time. The results show that p53 loss of function could make the tumour microenvironment more acidic during the period of time (i.e., 25 days) considered here. This is mainly because the p53- tumour grows faster and therefore more waste products are accumulated inside the tumour making it more acidic.

**Fig 7:**
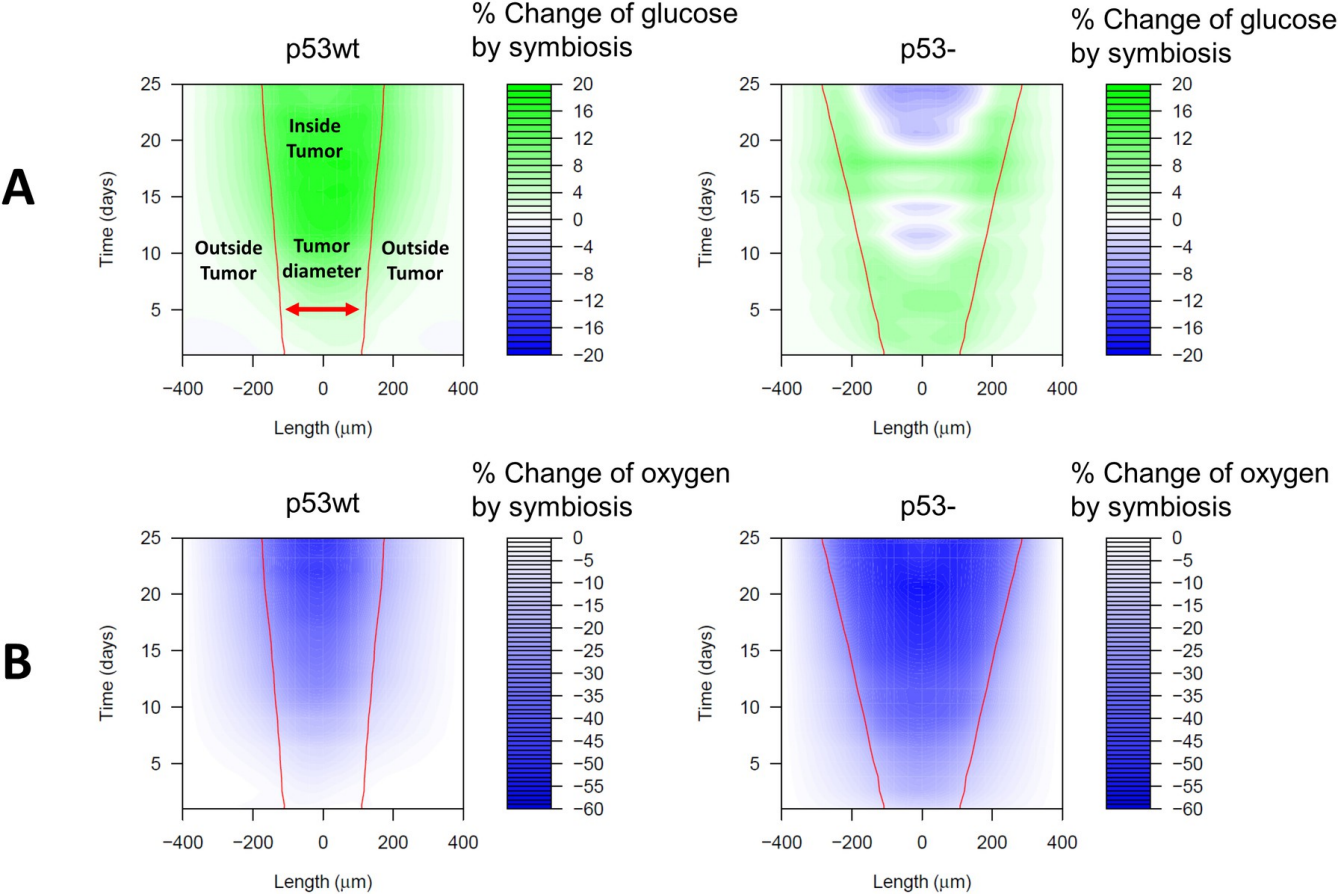
Symbiosis-induced percentage change of glucose and oxygen in the tumour microenvironment. **(A-B)**. Percentage change of (A) glucose and (B) oxygen in the tumour microenvironment over time for tumour growth with p53wt and p53- status. Length and red lines are as described in Fig 6. The model was run with MCT1wt and MCT1- status. The percentage change of glucose and oxygen in the tumour microenvironment was calculated over time. The heat maps suggest that symbiosis increases glucose level while decreasing oxygen level at the tumour boundary. More supplementary results are shown in S8 Fig.

Fig 6A–6D compare the oxygen, glucose, lactate and pH levels in the medium over time for p53wt and p53- simulations. It shows that oxygen and glucose are depleted at the center of the tumour because when tumour size increases less nutrients would diffuse to the center of the tumour, or they would be consumed at higher rates by the cells at the tumour rim. Lactate accumulates at the center of the tumour making it more acidic. For the p53- tumour, we can see that the tumour microenvironment becomes more acidic compared to the p53wt tumour. Fig 7A and 7B show percentage change of glucose and oxygen in the medium due to metabolic symbiosis for both p53wt and p53- tumours. In the p53wt case, the glucose at the center is increased by symbiosis, while in the p53- simulation, the glucose level at the tumour center is increased by symbiosis only at the initial phase of the tumour growth. As already shown in Fig 4B, the tumour gets larger when p53- cells grow with, rather than without, symbiosis, and hence more glucose is consumed by the symbiotic tumour (MCT1wt) than by the non-symbiotic tumour (MCT1-). This would be the reason we see a lower glucose concentration in the center of the tumour at the later stages of symbiotic p53- tumour growth. However, it is vital to note that the glucose level at the tumour boundary where most of the proliferative cells reside is always higher due to symbiosis (Fig 7A). With symbiosis occurring between oxygenated and hypoxic tumour cells, oxygenated cells would consume more oxygen for mitochondrial lactate metabolism and therefore symbiosis would result in less oxygen throughout the tumour compared to a non-symbiotic tumour (Fig 7B).

To get a clearer insight into how symbiosis affects the levels of extra-cellular environmental glucose and oxygen, it is necessary to compare symbiotic and non-symbiotic tumours of equal sizes. As tumours grow differently in the two conditions, this would be possible only at the initial stage. Therefore, we performed a sensitivity analysis by varying the initial sizes of the tumour, and compared the nutrient levels between symbiosis and non-symbiosis only at the initial stage of the tumour growth. This allowed us to simulate the contribution of symbiosis on nutrient levels for different sizes of the tumour. S8A Fig shows that both p53wt and p53- tumours have an increase in glucose level in the environment due to symbiosis. We observe that the glucose level increment for the largest p53- tumour (i.e., with 500 initial cells) is particularly strong, indicating that the glucose level at the center of the tumour would be increased by about 100% due to symbiosis. S8B Fig indicates that metabolic symbiosis would result in a low concentration of oxygen in the tumour microenvironment, and thus a higher degree of hypoxia in the tumour, as already shown in Fig 3.

In summary, our simulations illustrate the interaction between p53 status and metabolic symbiosis, and its effect on tumour growth and environmental oxygen and glucose levels. Specifically, symbiosis would increase glucose level (i.e., prevent glucose depletion due to glycolysis) while it would decrease oxygen level at the tumour boundary. These symbiosis-induced nutrient conditions (i.e., increased glucose and hypoxic conditions) of the microenvironment may contribute to further p53- selection or the modulation of other metabolic pathways [47]. It should be noted that although microenvironmental glucose level is increased by symbiosis, it is not increased beyond the glucose level set at the boundary of the simulation domain (i.e., 5 mM) in which this behaviour is similar to that of actual tumour glucose levels, which do not increase beyond blood glucose level.

### 3.4 Heterogeneity in the local microenvironment affects the extent of metabolic symbiosis

We asked to what extent changes in the gradient of diffusible substances and cell heterogeneity could impact on the observed metabolic symbiosis. To investigate this, the model was run with different nutrient conditions, with p53 wt/- and MCT1 wt/-, simulating heterogeneous environmental conditions. First, we varied the levels of glucose, oxygen and lactate, for p53wt or p53- cells. When the glucose and lactate levels are kept at a low value (1 mM), the p53wt tumour shrinks over time because dead cells are not replaced by new cells due to lack of nutrients (S9A and S9B Fig). However, in a p53- tumour, cell apoptosis is stopped and those cells can also use the initially provided lactate (5mM) and hence they can proliferate through lactate metabolism when there is enough oxygen. When lactate decreases as it is consumed by tumour cells, the cells are starved and hence OXPHOS cell population starts to decrease after 25 days (S9C and S9D Fig). When we maintain a constant lactate level (5mM) at low glucose level (1 mM), we can see that the tumour continues its growth through lactate metabolism when enough oxygen is available (S9A and S9C Fig). Then, when we maintain low oxygen (3% O_2_) and enough glucose (5 mM) levels at the boundary of the medium, we can see that cells use glycolysis for both p53wt and p53- tumours and the tumour growth continues through pure glycolysis (S9B and S9D Fig). S10 Fig further shows how the tumour responds to different combinations of oxygen and glucose levels. At low oxygen level (3% O_2_), glycolysis is the dominant pathway and at intermediate oxygen level (6% O2) we can see that the glycolysis and OXPHOS cell populations are comparable. Then, at the high oxygen level (9% O_2_), the dominant cell population is OXPHOS because there is sufficient oxygen for mitochondrial metabolism of either glucose or lactate.

The half-saturation coefficients (*i*.*e*., *K*_*O*2_, *K*_*G*_ *etc*.) quantify the sensitivity of nutrient uptake by tumour cells to the variation of environmental nutrient levels. S11 Fig shows that cell’s metabolic pathway would be more sensitive to the half-saturation coefficient of oxygen than that of glucose for the model.

Next, we simulated two p53- cell populations in direct competition; one population had metabolic symbiosis capacity (MCT1wt) and the other population did not (MCT1-).

**Fig 8:**
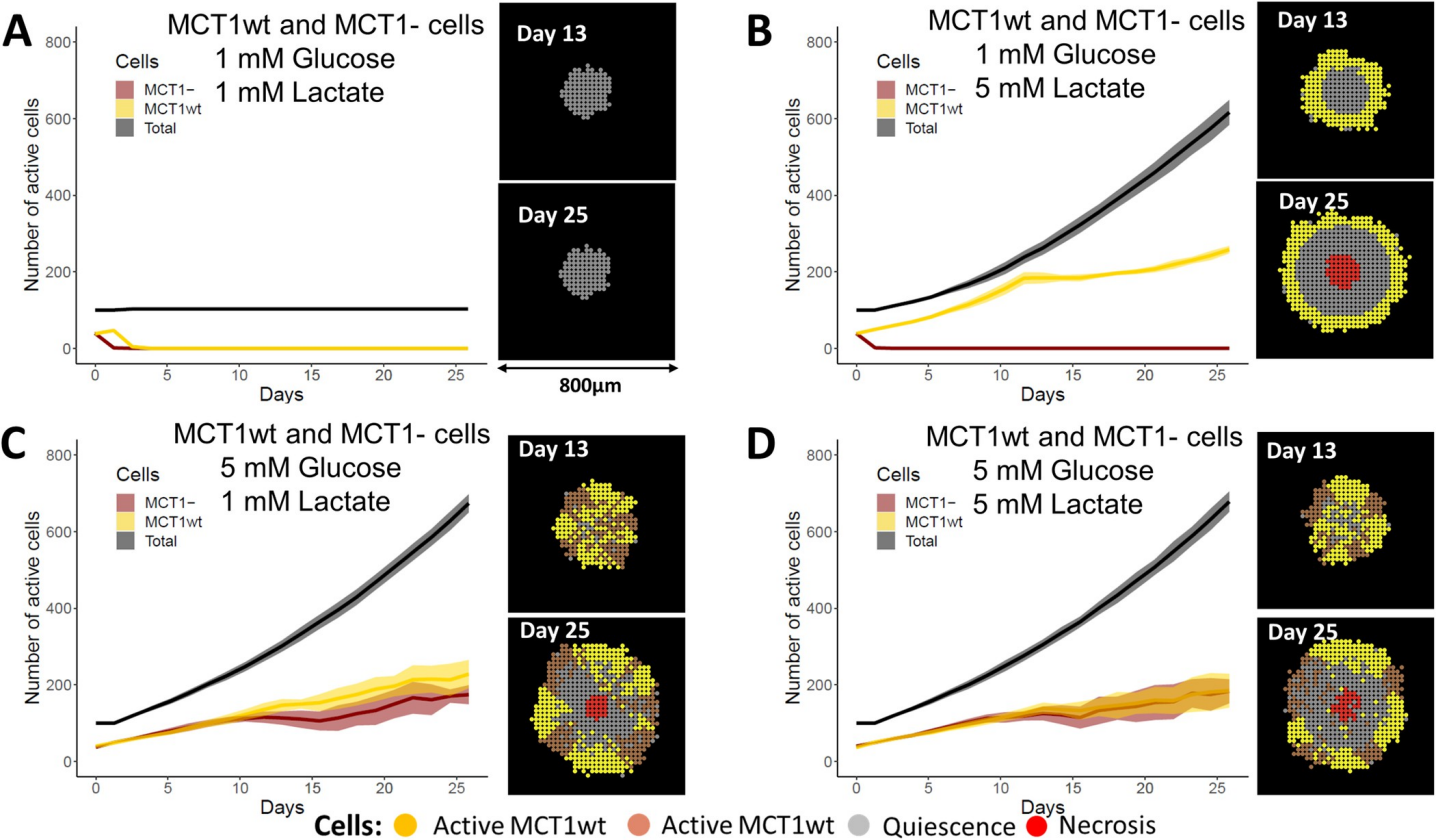
Competition between symbiotic and non-symbiotic tumour cells. Graphs depicting the number of active cells for two p53- cell populations with or without symbiotic capacity, with MCT1wt and MCT1-, respectively. The ratio of MCT1wt: MCT1- was 1:1 at the start of the simulations. Cells were allowed to compete at 6% of oxygen and varying levels of glucose and lactate maintained at the boundary of the computational domain. The number of total, active MCT1wt and active MCT1- cells are shown. **(A)**. 1mM glucose and 1 mM lactate, **(B)**. 1mM glucose and 5 mM lactate, **(C)**. 5 mM glucose and 1 mM lactate, **(D)**. 5 mM glucose and 5 mM lactate. The results show that symbiotic cells would not get a significant competitive advantage over non-symbiotic cells if there is sufficient glucose in the tumour microenvironment.

Initially, two cell populations were randomly mixed at a 1:1 ratio. The glucose and lactate levels were set to either 1 mM or 5 mM. When both glucose and lactate are at the lowest levels, we can see that there is no tumour growth (Fig 8A). However, when we increase lactate level to 5 mM at the lowest glucose level, MCT1wt cell population grows while MCT1- cell population is eliminated from the tumour because there is not sufficient glucose for the survival of MCT1- cells (Fig 8B). The MCT1wt cell population gets a smaller growth advantage over MCT1- population at 5 mM glucose and 1 mM lactate levels (Fig 8C). When both nutrients are at 5 mM, there is no clear difference between two cell populations because there would be enough glucose for MCT1- cells and enough lactate and glucose for MCT1wt cells for their growth (Fig 8D).

The above results show that the nutrient levels in tumour cells not only induce a switch to different metabolic pathways for tumour survival, but also would apply a selection pressure on the spatial competition between symbiotic and non-symbiotic cells.

### 3.5 GLUT and MCT1 as actionable targets to disturb metabolic symbiosis

Inhibitors of metabolic pathways have been investigated as potential cancer therapeutics and various proteins involved in glucose metabolism such as GLUT, HK and LDH have been considered [48,49]. Lactate transporter inhibitors block the Warburg and reverse Warburg effects and hence lactate-driven OXPHOS would be reduced [50–53]. However, blocking either glucose or lactate intake transporters would potentially force cancer cells to switch between glucose and lactate metabolic pathways and exhibit therapy resistance. We hypothesized that tumour cells would switch between glucose and lactate metabolic pathways when either pathway was blocked. Therefore, we simulated how the tumour would respond to GLUT1 and MCT1 inhibitors (denoted as GLUT1i and MCT1i) at varying concentration levels and investigated whether tumours would show therapy resistance against respective drugs. Both inhibitors were introduced at the beginning of simulations at a constant level at the boundary of the simulation domain. We assumed that these inhibitors would reach tumour cells by pure diffusion through the tumour microenvironment. It was assumed that the maximum inhibitory level of each drug is 85% and the drug would inhibit the respective membrane protein at a probability of $\frac{0.85*[Inhibitor]}{IC50+[Inhibitor]}$, where [*Inhibitor*] and *IC50* are the inhibitor concentration near the cell and half-maximum inhibitory concentration of the inhibitor, respectively.

**Fig 9:**
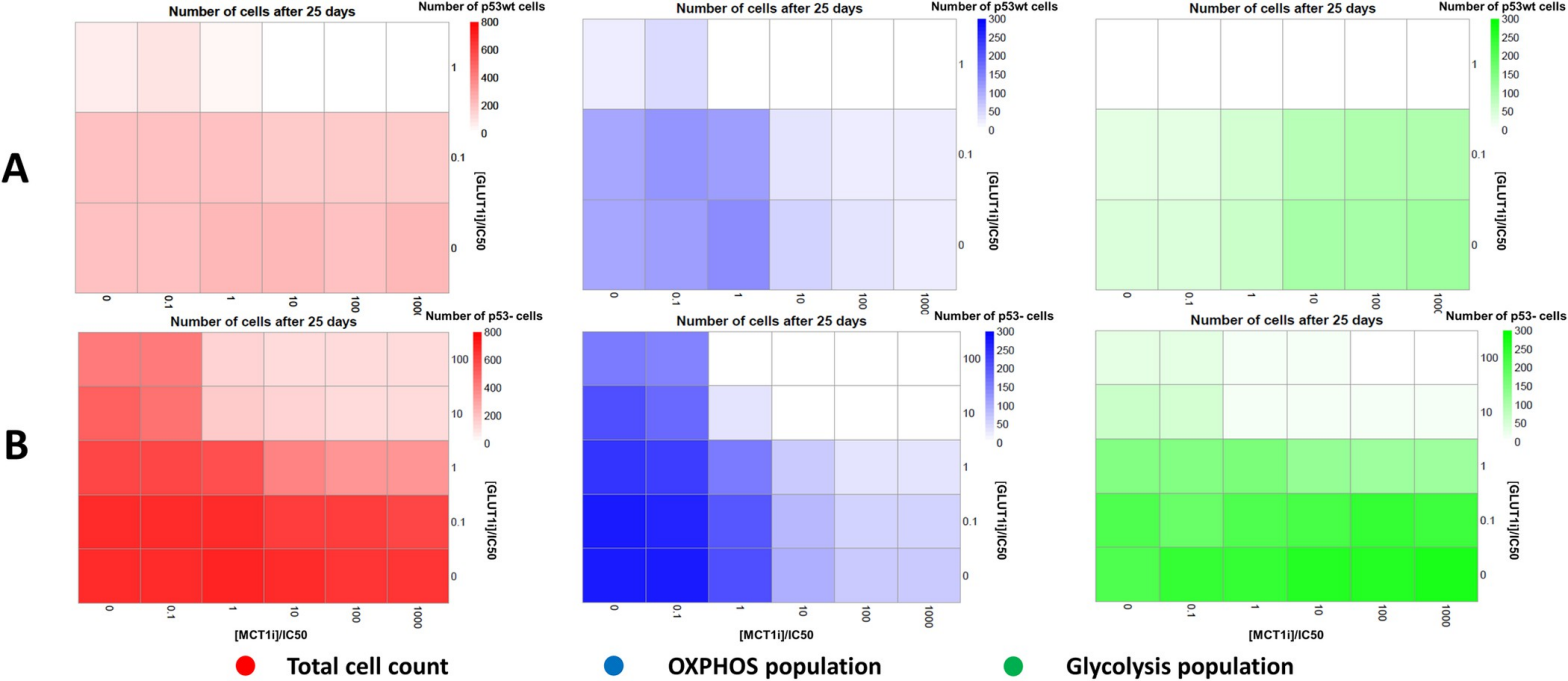
MCT1 and GLUT1 inhibition with continuous drug administration. Heatmaps depicting how MCT1 and GLUT1 inhibitors (denoted as MCT1i and GLUT1i) interfere with glycolysis, OXPHOS, and tumour growth. The MCT1i concentration, [MCT1i], was varied up to 1000 times of its half-maximal inhibitory concentration (IC50) value while GLUT1i concentration, [GLUT1i], was varied up to 100 times of its IC50 value. Inhibitor concentration was maintained at the boundary of the computational domain throughout the simulation time. **(A)**. Number of total cells, OXPHOS cell population and glycolytic cell population of p53wt tumour after 25 days of growth. **(B)**. Number of total cells, OXPHOS cell population and glycolytic cell population of p53- tumour after 25 days of growth. The initial tumour consisted of 100 cells, with either with p53wt or p53- status.

Fig 9A shows p53wt tumour cells at the end of the simulation under different combinations of MCT1i and GLUT1i levels. We can see that the OXPHOS cell population dominates in the tumour at low concentration of both drugs. As the MCT1i drug concentration is increased, the glycolytic cell population dominates over OXPHOS (S12A–S12C and S13A Figs). At GLUT1i concentration of 1×IC50, p53wt tumour growth is completely stopped at higher concentrations of MCT1i because glycolysis is disturbed, and hence apoptotic cells are not replaced by new cells. For p53- cells (Figs 9B, S12D–S12F, and S13B), we can see that we need a higher concentration of drugs to reduce tumour growth and it shows that p53- tumour may exhibit more resistance against these inhibitors. These results show that p53- tumour cells would exhibit some therapy resistance for GLUT1 inhibitors by using lactate metabolism. However, metabolically active cells (i.e., green and blue coloured cells) would decrease as the concentrations of both drugs are increased.

**Fig 10:**
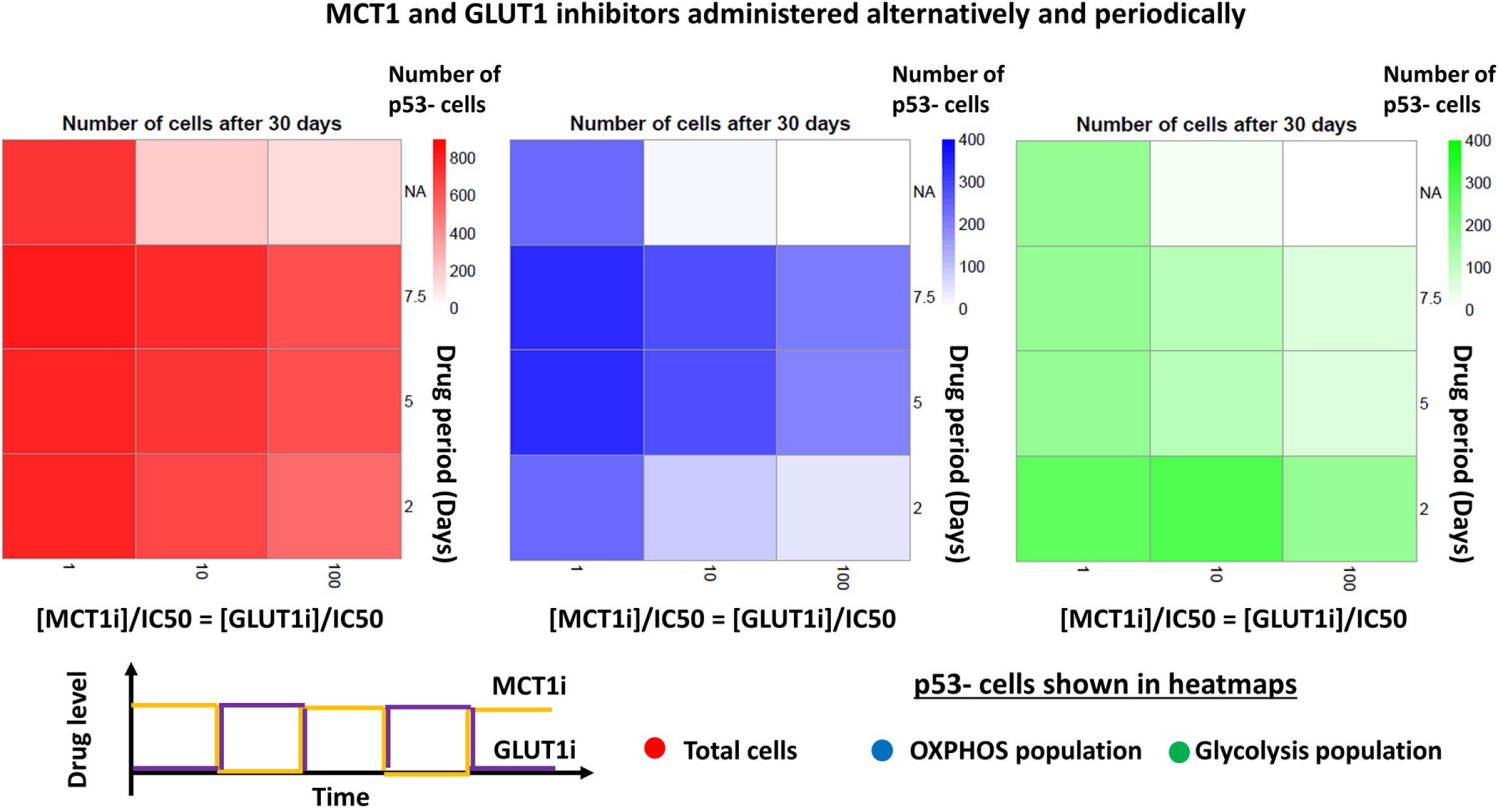
MCT1 and GLUT1 inhibition with alternative and periodic drug administration. Heatmaps depicting how MCT1 and GLUT1 inhibitors interfere with glycolysis, OXPHOS, and tumour growth when both drugs were given alternatively and periodically. Both MCT1i and GLUT1i concentrations were kept at same level, and it was maintained at 1, 10 and 100 of respective half-maximal inhibitory concentration (IC50) value. Inhibitor concentration was maintained at the boundary of the computational domain and varied periodically throughout the simulation time. The initial tumour consisted of 100 cells, with p53- status. Number of total cells, OXPHOS cell population and glycolytic cell population of p53- tumour after 30 days of growth are shown. The period “NA” represents the simulation with continuous supply of both drugs at half of the respective concentration.

**Fig 11:**
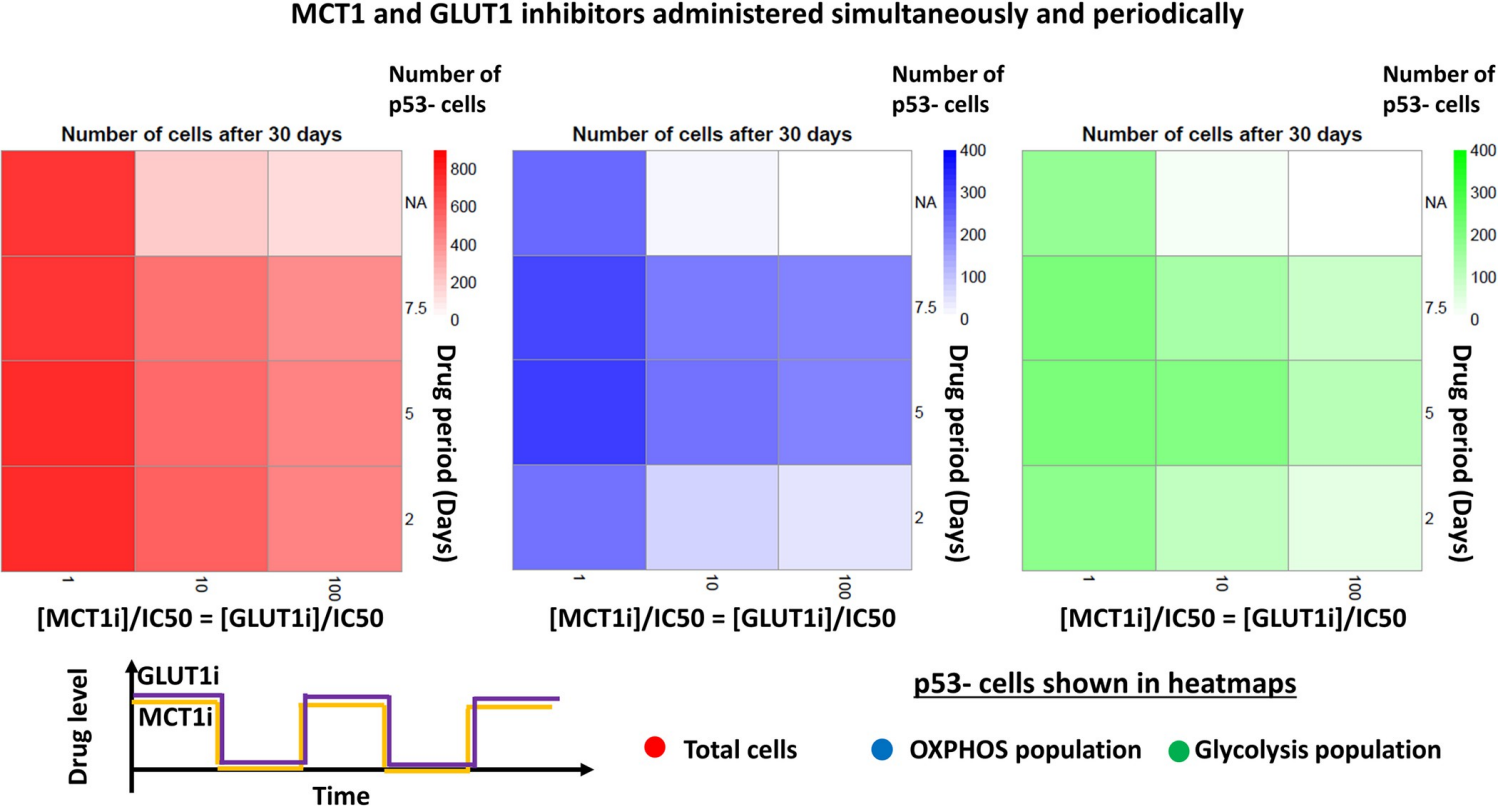
MCT1 and GLUT1 inhibition with simultaneous and periodic drug administration. Heatmaps depicting how MCT1 and GLUT1 inhibitors interfere with glycolysis, OXPHOS, and tumour growth when both drugs were given simultaneously and periodically. Other simulation conditions remained consistent with those in Fig 10. Number of total cells, OXPHOS cell population and glycolytic cell population of p53- tumour after 30 days of growth are shown. The period “NA” represents the simulation with continuous supply of both drugs at half of the respective concentration.

We also investigated the effect of different drug application strategies on tumour growth. In the first scenario, MCT1 inhibitor was initially administered, followed by alternative treatments of MCT1 and GLUT1 inhibitors, each for a fixed period. In the second scenario, both MCT1 and GLUT1 inhibitors were simultaneously applied for a fixed period, followed by a therapy-free interval of same length, and this cycle was repeated. Simulations were performed for three levels of drug concentrations (i.e., [MCT1i]/IC50 = [GLUT1i]/IC50 = 1, 10 and 100) and three levels of periods (i.e., 2, 5 and 7.5 days). A third scenario was used as the control in which both inhibitors at half of above drug concentration levels were continuously administered throughout the simulation time. The results show that scenario one is the least effective sequential strategy because tumour cells can switch between glycolysis and OXPHOS when drugs were applied alternatively (Figs 10 and S14A). In the second scenario, both OXPHOS and glycolytic cell populations increase during therapy free intervals and therefore tumour would grow at a reduced rate (Figs 11 and S14A). The results also show that continuous administration of higher concentration levels of inhibitors (i.e., 10, 100) can completely stop tumour growth (S14B Fig).

Looking at previous experimental work of use of MCT1 and GLUT inhibitors, **Guan and Morris** (51) have shown that MCT1 inhibitors AZD3965 and CHC can block lactate uptake and hence tumour growth of 4T1 murine mammary carcinoma cell line. **Guan, Rodriguez-Cruz** (50) have also shown that AR-C155858 and AZD3965 would reversibly inhibit MCT1 of 4T1 cells. As reported in [54], AR-C155858 is effective in reducing mammosphere formation of MCF7 and T47D cell lines. All these findings indicate that MCT1 would be a potential therapeutic target for breast cancer. However, our results suggest that blocking MCT1 would force tumour cells to switch to glycolysis and develop resistance against MCT1 inhibitors regardless of the p53 status. A recent clinical trial of the MCT1 inhibitor AZD3965 showed that some patients had slight increase in ^18^FDG uptake suggesting a lack of anti-tumour effect of the drug [55]. Yet, it should be noted that blocking MCT1 can disrupt metabolic symbiosis and therefore it would have unfavourable effect on tumour growth because glucose would be depleted faster when symbiosis is blocked as discussed earlier. Various GLUT inhibitors such as Glutor and Glupin seem to be effective in impairing glucose metabolism of tumour cells [56,57], but our results suggest that tumour cells may resist GLUT inhibitors if the microenvironment has enough lactate.

Our results show that even though metabolic symbiosis can be disturbed and hence tumour growth can be reduced by targeting MCT1 and GLUT1 transport proteins in concordance with previous experimental studies, the tumour cells can also exhibit some resistance against those inhibitors by switching between glucose and lactate metabolic pathways. However, the model also predicts that a combination of MCT1 and GLUT1 inhibitors would be synergistic and work against drug resistance and the tumour growth can be completely stopped if both inhibitors are applied at an appropriate concentration. Though tumour growth can be completely stopped at high inhibitor concentrations, it is important to note that inhibitor level and schedule need to be carefully selected in clinical trials with consideration of potential normal tissue toxicity.

### 3.6 Metabolic symbiotic dynamics would depend on the characteristics of cells and microenvironment

As many of the assumptions were taken from existing literature, we performed a sensitivity analysis based on Latin Hypercube Sampling (LHS) and partial rank correlation coefficients [38,58] to identify the parameters that more strongly affected our predictions. The model parameters related to diffusion and activation thresholds of substances (Table C in S1 Text) were varied within 20% from their baseline values. The total number of parameters was 20. To perform LHS, the range of each parameter (i.e., 80% - 120% of the baseline value) needs to be divided into an equal number of bins. The number of bins was chosen as 40 (for LHS, the number of bins needs to be more than 4×number of parameters/3) and then the LHS input parameter matrix was created according to the LHS sampling method. Then, our model was run for the parameter set in each bin and the model outputs were recorded against each parameter set. The values of each parameter and each output were ranked across bins from 1 to 40 and then the partial correlation coefficient was calculated between each parameter and each output.

S15 Fig shows the statistically significant (p <0.05) partial correlation coefficients between the model parameters and four model outputs. We can see that the model outputs are correlated with some model parameters, but in a time dependent manner. The results show that the number of total cells, OXPHOS, glycolytic, and necrotic cells are not significantly sensitive to most of the parameters. This could be because the tumour size would mainly be determined by the phenotypes of boundary cells rather than inner cells which have greater contact inhibition due to denser packing. The total number of cells is negatively correlated with the glucose activation threshold (i.e., minimum glucose level needed to activate Glucose_supply node of the network. See S1 Text for more details about activation threshold) and lactate diffusion coefficient. This behaviour is intuitive because as the glucose activation threshold is increased, a fewer number of cells would have glycolysis and when the lactate diffusion coefficient is increased more lactate would diffuse out from the tumour and hence less lactate would be available for OXPHOS. The oxygen diffusion and half-saturation coefficients have positive correlation with OXPHOS at early and later stages of tumour growth, respectively. These two parameters would increase the oxygen level in the medium and hence this dependency could be expected. The oxygen consumption and activation threshold are strongly and negatively correlated with OXPHOS at later stages of tumour growth because increase of these parameters would cause lack of oxygen for respiration.

Lactate diffusion coefficient and activation threshold also have strong negative correlation with OXHPOS at early stage of the growth because increase of these parameters would weaken lactate intake through MCT1 transporters. The glucose activation threshold and lactate diffusion coefficient negatively correlate with glycolysis at later stages of tumour growth. The strong positive correlation between the lactate activation threshold and glycolysis at the early stage of the growth would be due to lactate intake being disturbed by increase in the threshold and hence more tumour cells tend to use glycolysis rather than lactate-fuelled OXPHOS. The oxygen consumption rate and glucose deficient necrosis threshold have some positive correlations with necrosis because both parameters would enhance nutrient depleted conditions in the medium.

S16 Fig shows how symbiosis-induced tumour growth correlates with model parameters. The glucose activation threshold strongly and positively correlates with growth advantage gained by symbiosis. This is because an increased activation threshold could decrease glucose consumption and glycolysis and it would then force more tumour cells to maintain their growth through lactate metabolism. Therefore, as glucose activation threshold is increased, tumour cells which have symbiotic capacity would gain a growth advantage over non-symbiotic tumour cells.

Our results show that tumour has a symbiosis-induced growth advantage and domination of the mitochondrial ATP producing cell population over glycolytic cell population for the whole LHS parameter space (S17 Fig). These results suggest that a 20% perturbation of the baseline parameters would not change qualitative behaviour of the model output and therefore the conclusion of our work would remain the same throughout the LHS parameter space. However, if changes were beyond these levels the specific conditions would need to be set, and the simulations reassessed.

## 4. Conclusions

Agent-based modelling is a powerful simulation technique adopted in many fields of sociology, engineering, and biology to study complex emerging behaviours of heterogeneous systems. Here, we used and extended a multi-scale framework merging multi-agent modelling, diffusion-reaction and stoichiometric equations, and Boolean networks to understand how metabolic symbiosis between oxygenated and hypoxic tumour cells influence tumour growth. By integrating these diverse modelling approaches, including a Boolean network describing intercellular molecular interactions, we gained a deeper understanding of the complex interactions shaping the tumour microenvironment and influencing overall tumour progression. The discrete nature of Boolean networks allowed us to understand interactions between gene alterations and metabolic symbiosis that enhance or reduce tumour survival. Specifically, we show that:

- Glucose level in the tumour microenvironment can be maintained by metabolic symbiosis and that this symbiosis helps hypoxic tumour cells survive through glycolysis, using this extra-cellular glucose. We found that the oxygen level of the medium would be depleted faster due to symbiosis and then more tumour cells would switch to a hypoxic state. The nutrient conditions in the medium would vary over time as tumour evolves. It is well-known that hypoxic tumour cells can exhibit cancer therapy resistance particularly radiotherapy [59] and therefore, this metabolic symbiosis would indirectly increase therapy resistance. For instance, as it is clear in the literature [60,61], blocking lactate uptake would sensitize tumour xenografts to radiotherapy.
- Metabolic symbiosis would allow more glycolytic cells to switch to lactate-driven OXPHOS and hence more glucose would be available for the remaining glycolytic cells. Therefore, this metabolic cooperation would increase the active cell population in the tumour. The dependence on glucose may explain some of relationship of diabetes mellitus to cancer incidence and prognosis.
- As glucose and oxygen levels increase in the medium, the glycolytic and OXPHOS cell populations respectively increase. The outcome of the direct competition between symbiotic and non-symbiotic cells of the tumour would depend on the environmental nutrient levels. For instance, symbiotic cells would have a growth advantage in a glucose-limited lactate medium. The pathways in which alteration (i.e., under or over expressions) in the regulatory network would have beneficial or detrimental effects due to symbiosis on the tumour growth were identified.
- Some gene alterations (i.e., knockout or enrichment) can interact with metabolic symbiosis to decrease tumour growth. Furthermore, both p53wt and p53- cells show some resistance against MCT1 inhibitors. However, use of MCT1 inhibitors in combination with GLUT1 inhibitors may disrupt metabolic cooperation between oxygenated and hypoxic cell populations. The results also indicate that administering both inhibitors simultaneously is more effective than employing a sequential therapy that alternates between the two drugs.

Our sensitivity analysis shows that the model outputs are sensitive to some model parameters only at different stages of tumour growth. However, the results show that the qualitative behaviour of the model would remain the same in whole parameter space which is within ±20% from the baseline value of each parameter.

The present model is a relatively simplified model of the metabolic symbiosis between hypoxic and oxygenated tumour cells. The model could be used to understand how metabolic changes induced by therapy in different genetic backgrounds interact. The utilization of a Boolean network to describe intercellular molecular interactions greatly facilitated our study, enabling us to easily investigate the effects of a range of gene alterations on metabolic symbiosis. While our Boolean network effectively captures the binary state of gene activity-either active or inactive-it falls short in representing the continuous spectrum in gene expression. To address this limitation, a potential enhancement involves expanding the Boolean nodes with continuous or multiple levels, representing low, intermediate, and high gene expression states. This refinement would better align with the dynamic nature of gene expression, providing a more accurate representation of the underlying biological processes. Future work should also consider including pathways such as lipid metabolism signalling pathway, adding further microenvironmental features (fibroblast, ECM, immune cells, and angiogenesis etc.), tumour heterogeneity and therapy modelling. Ultimately, any simulation result would need to be confirmed in validation in pre-clinical setting, but our findings could help in guiding the experimental design of such studies.

## Supporting information

S1 Text Methodology and Parameters. Different spatial and temporal scales of the model and how these scales interact each other are described. More details about the cell regulatory network and model parameters are given.
(DOCX)

S1 Chart Breast Cancer Cell Lines Gene Expressions. Breast cancer cell line gene expression data under normoxic and hypoxic conditions are given. The unit is log2 (FPKM+2). The data were used to produce S4 Fig.
(CSV)

S1 File Cell Regulatory Network. This is the source file to open the network with GINsim software (http://ginsim.org) and all the Boolean logical conditions and respective supporting evidence can be seen there. Download GINsim software from http://ginsim.org and upload S1 File to visualise cell regulatory network and logical conditions.
(ZGINML)

S2 File Cell Regulatory Network. A high resolution image of the network.
(PNG)

S1 Fig Multi-scale modelling framework. The extra-cellular scale, an equation-based model, describes distributions of diffusible substances in the tumour microenvironment. The cellular scale, an agent-based model which is a cellular automaton model, describes cell-cell and cell-microenvironmental interactions. The intra-cellular scale, an agent-based model which is a Boolean network, describes subcellular molecular interactions. The three scales are coupled each other and information are shared between them.
(DOCX)

S2 Fig The cell regulatory network of our model (microC model). The modified MAPK network decides the cell phenotype based on inputs obtained from the microenvironment. Oxygen_supply, Glucose_supply, EGFR_stimulus, cMET_stimulus, FGFR_stimulus, TGFBR_stimulus, DNA_damage and Growth_inhibitor are inputs to the network. The inhibitor nodes (EGFRI, cMETI, FGFRI, GLUT1I, MCT1I, and MCT4I) are also inputs. The inhibitor nodes are activated by respective drugs (e.g. GLUT1I is activated by GLUT1D, MCT1I is activated by MCT1D and so on). The Boolean network is updated asynchronously, and cell phenotypical outputs are calculated. The outputs are Proliferation, Apoptosis, Necrosis and Growth_Arrest. The yellow-colored nodes are diffusible substances. The newly added interactions to the original MAPK network taken from [30] are shown in dotted lines. The solid and dotted green lines are positive interactions and solid and dotted red lines are negative interactions. More details about Boolean logical conditions at each node are given in [16,30] and Tables A and B in S1 Text. A high-resolution image of this network is available in S1 and S2 Files.
(DOCX)

S3 Fig Different time scales are used for different processes. The smallest time step is defined as the time for updating one node of the regulatory network (*T*_*Network*_). Cell phenotype and diffusion fields are updated at red (*T*_*Phynotypes*_) and green (*T*_*Difusion*_) ticks, respectively. A cell which is older than the cell division time (*T*_*Division*_) can divide if its phenotype is Proliferation.
(DOCX)

S4 Fig **(A)**. Expression of our network genes of some Triple Negative Breast Cancer (TNBC) and non-TNBC cell lines under normoxia (N) and hypoxia (H). The unit is log2 (FPKM+2). **(B)**. The gene expressions (EXP) at hypoxia are normalized by respective expressions at normoxia (log2 (EXP at hypoxia/ EXP at normoxia)). The data used to produce these Figs are given in S1 Chart.
(DOCX)

S5 Fig **(A)**. TCGA breast cancer samples with high mRNA expression of network genes relative to normal samples. Genes are ordered from high to low of percentage of high mRNA samples. **(B)**. TCGA breast cancer samples with low mRNA expression of network genes relative to normal samples. Genes are ordered from high to low of percentage of low mRNA samples. The horizontal axis shows samples with high (red) or low (blue) expressions of respective genes. We considered the gene as over-expressed when the standard deviation was above +3 with respect to normal samples and under-expressed as when it was below -3.
(DOCX)

S6 Fig sigQC metrics were calculated for our network genes in TCGA and CCLE breast cancer RNA Seq. datasets. **(A)**. Correlation coefficients between genes are shown for TCGA data. **(B)**. Correlation coefficients between genes are shown for CCLE data. **(C)**. Distribution of correlation coefficients and comparison of sigQC metrics calculated for genes using TCGA and CCLE datasets. The distribution of the metrics are fairly similar between two datasets.
(DOCX)

S7 Fig Metabolic symbiotic simulations with network gene alterations (Enriched (+) and Knockout (-) status) and wild type (WT) status. **(A)**. Temporal variation of symbiosis index at each gene enrichment status. **(B)**. Temporal variation of symbiosis index at each gene knockout status. **(C)**. Whether symbiosis-induced growth is greater than non-symbiotic tumour growth with each gene enrichment is shown. **(D)**. Whether symbiosis-induced growth is greater than non-symbiotic tumour growth with each gene knockout status is shown. The results show that clusters of gene alterations can be identified, which enhance symbiosis while some other gene alterations reduce symbiosis (A, B). Colors indicate symbiosis index (A, B) and p values (C, D). p values from 0 to 0.05 are shown in red to white color scale and p values ≥ 0.05 are shown in grey color. **(E)**. Genes are clustered based on p-value is less than 0.05 (red) or not (white) of the results shown in Fig 4C (top-left), Fig 4D (top-right), S7C Fig (bottom-left), and S7D Fig (bottom-right).
(DOCX)

S8 Fig Symbiosis-induced changes of glucose and oxygen of the microenvironment over time. Here, the Length is the cross section through the center of the tumour. The heat maps show the variation of the percentage change of oxygen and glucose due to metabolic symbiosis under p53wt and p53- status, and at different initial tumour sizes. **(A)**. The symbiosis would increase the glucose level in the medium. **(B)**. The symbiosis would decrease the oxygen level in the medium.
(DOCX)

S9 Fig Tumour growth under different environmental conditions (different levels of glucose, lactate and oxygen) are shown for p53wt and p53- cells. **(A)**. p53wt cells with 6% oxygen level. **(B)**. p53wt cells with 3% oxygen level. **(C)**. p53- cells with 6% oxygen level. **(D)**. p53- cells with 3% oxygen level. The results show that both glucose and lactate metabolism can fuel tumour growth when tumour is well-oxygenated.
(DOCX)

S10 Fig Tumour growth curves are shown for different combinations of oxygen and glucose. As the oxygen level is increased, more tumour cells switch to OXPHOS because they have both glucose and lactate as the energy source for mitochondrial ATP production. The oxygen level at the boundary of the simulation domain (square box) was maintained at 3%, 6%, and 9% O2 while the glucose level was at 1 mM (A), 5 mM (B) and 10 mM (C). Note that oxygen and glucose levels in the tumour were much lower than these boundary values.
(DOCX)

S11 Fig The effect of half-saturation coefficients of oxygen and glucose on the metabolic pathways is shown. The half-saturation coefficient of oxygen was kept at 0.045% oxygen (A), 0.45% oxygen (B) and 4.5% oxygen (C) while the half saturation coefficient of glucose was varied to 0.004 mM, 0.04 mM and 0.4 mM. The pathways seem more sensitive to the variation of the half-saturation coefficient of oxygen.
(DOCX)

S12 Fig Tumour growth over time at different combinations of GLUT1 and MCT1 inhibitors. [GLUT1i]/IC50 and [MCT1i]/IC50 were varied from 0 to 100 and 0 to 1000, respectively. **(A, B, C)**. p53wt tumour cells. Note that the tumour is completely disappeared at [GLUT1]/IC50 = 10. **(D, E, F)**. p53- tumour cells. Temporal variations of total cells (A, D), glycolytic cells (B, E), and OXPHOS cells (C, F) are shown.
(DOCX)

S13 Fig MCT1 and GLUT1 inhibition. Bar charts depicting how MCT1 and GLUT1 inhibitors (denoted as MCT1i and GLUT1i) interfere with glycolysis, OXPHOS, and tumour growth. The MCT1i concentration, [MCT1i], was varied up to 1000 times of its half-maximal inhibitory concentration (IC50) value while GLUT1i concentration, [GLUT1i], was varied up to 100 times of its IC50 value. Inhibitor concentration was maintained at the boundary of the computational domain throughout the simulation time. **(A)**. Number of glycolytic and mitochondrial ATP producing cells of p53wt tumour after 25 days of growth. **(B)**. Number of glycolytic and mitochondrial ATP producing cells of p53- tumour after 25 days of growth. The initial tumour consisted of 100 cells, either with p53wt or p53- status.
(DOCX)

S14 Fig Dynamics of total, glycolytic (glycoATP), and OXPHOS (mitoATP) cell populations over time under different application strategies of MCT1 and GLUT1 inhibitors. The simulations were initiated with a population of 100 p53- cells, and the inhibitor concentrations were set to [MCT1i]/IC50 = [GLUT1i]/IC50 = 10. **(A)**. In the first scenario, MCT1 inhibitor was initially administered, followed by alternating treatments of MCT1 and GLUT1 inhibitors for a constant period. In the second scenario, both MCT1 and GLUT1 inhibitors were simultaneously applied for a constant period of time, followed by a therapy-free interval, and this cycle repeated each for 5 days, until the total simulation time reached 30 days. The period was set to 2, 5 and 7,5 days. **(B)**. The control cases. The inhibitors were set to MCT1i]/IC50 = [GLUT1i]/IC50 = 5 and they were continuously applied (Left). No therapy was applied in this case (Right).
(DOCX)

S15 Fig Partial correlation coefficients between model parameters and outputs over time. The model outputs are total number of cells, OXPHOS cell, glycolytic cell, and necrotic cell populations. The outputs have significant correlations with some parameters in a time dependent manner.
(DOCX)

S16 Fig Partial correlation coefficients between symbiosis-induced growth increment of tumour and model parameters over time. Glucose activation threshold and oxygen consumption rate have strong positive correlations with metabolic symbiosis while glucose diffusion coefficient and oxygen activation threshold have negative correlations with symbiosis.
(DOCX)

S17 Fig **(A)**. Number of active tumour cells with symbiosis (MCT1wt) and without symbiosis (MCT1-) for each perturbed parameter set (PS1-40) and the baseline parameter set (PS-Base). **(B)**. Number of total cells, and mitochondrial and glycolytic ATP producing cells obtained at each parameter set.
(DOCX)

## References

[1] Fani R, Fondi M. Origin and evolution of metabolic pathways. Phys Life Rev. 2009;6(1):23–52. Epub 2009/01/08. doi: 10.1016/j.plrev.2008.12.003 . 20416849

[2] Martinez-Reyes I, Chandel NS. Cancer metabolism: looking forward. Nat Rev Cancer. 2021. Epub 2021/07/18. doi: 10.1038/s41568-021-00378-6 . 34272515

[3] Robertson-Tessi M, Gillies RJ, Gatenby RA, Anderson AR. Impact of metabolic heterogeneity on tumor growth, invasion, and treatment outcomes. Cancer Res. 2015;75(8):1567–79. Epub 2015/04/17. doi: 10.1158/0008-5472.CAN-14-1428 ; PubMed Central PMCID: PMC4421891. 25878146 PMC4421891

[4] Shamsi M, Saghafian M, Dejam M, Sanati-Nezhad A. Mathematical Modeling of the Function of Warburg Effect in Tumor Microenvironment. Sci Rep. 2018;8(1):8903. Epub 2018/06/13. doi: 10.1038/s41598-018-27303-6 ; PubMed Central PMCID: PMC5995918. 29891989 PMC5995918

[5] Cairns RA, Harris IS, Mak TW. Regulation of cancer cell metabolism. Nat Rev Cancer. 2011;11(2):85–95. Epub 2011/01/25. doi: 10.1038/nrc2981 . 21258394

[6] Semenza GL. Tumor metabolism: cancer cells give and take lactate. J Clin Invest. 2008;118(12):3835–7. Epub 2008/11/27. doi: 10.1172/JCI37373 ; PubMed Central PMCID: PMC2582934. 19033652 PMC2582934

[7] Yoshida GJ. Metabolic reprogramming: the emerging concept and associated therapeutic strategies. J Exp Clin Cancer Res. 2015;34:111. Epub 2015/10/09. doi: 10.1186/s13046-015-0221-y ; PubMed Central PMCID: PMC4595070. 26445347 PMC4595070

[8] de la Cruz-Lopez KG, Castro-Munoz LJ, Reyes-Hernandez DO, Garcia-Carranca A, Manzo-Merino J. Lactate in the Regulation of Tumor Microenvironment and Therapeutic Approaches. Front Oncol. 2019;9:1143. Epub 2019/11/19. doi: 10.3389/fonc.2019.01143 ; PubMed Central PMCID: PMC6839026. 31737570 PMC6839026

[9] Glancy B, Kane DA, Kavazis AN, Goodwin ML, Willis WT, Gladden LB. Mitochondrial lactate metabolism: history and implications for exercise and disease. J Physiol. 2021;599(3):863–88. Epub 2020/05/03. doi: 10.1113/JP278930 . 32358865 PMC8439166

[10] Sonveaux P, Vegran F, Schroeder T, Wergin MC, Verrax J, Rabbani ZN, et al. Targeting lactate-fueled respiration selectively kills hypoxic tumor cells in mice. J Clin Invest. 2008;118(12):3930–42. Epub 2008/11/27. doi: 10.1172/JCI36843 ; PubMed Central PMCID: PMC2582933. 19033663 PMC2582933

[11] Nakajima EC, Van Houten B. Metabolic symbiosis in cancer: refocusing the Warburg lens. Mol Carcinog. 2013;52(5):329–37. Epub 2012/01/10. doi: 10.1002/mc.21863 . 22228080 PMC9972501

[12] Kianercy A, Veltri R, Pienta KJ. Critical transitions in a game theoretic model of tumour metabolism. Interface Focus. 2014;4(4):20140014. Epub 2014/08/07. doi: 10.1098/rsfs.2014.0014 ; PubMed Central PMCID: PMC4071509. 25097747 PMC4071509

[13] Pisarsky L, Bill R, Fagiani E, Dimeloe S, Goosen RW, Hagmann J, et al. Targeting Metabolic Symbiosis to Overcome Resistance to Anti-angiogenic Therapy. Cell Rep. 2016;15(6):1161–74. Epub 2016/05/03. doi: 10.1016/j.celrep.2016.04.028 ; PubMed Central PMCID: PMC4870473. 27134168 PMC4870473

[14] Porporato PE, Dhup S, Dadhich RK, Copetti T, Sonveaux P. Anticancer targets in the glycolytic metabolism of tumors: a comprehensive review. Front Pharmacol. 2011;2:49. Epub 2011/09/10. doi: 10.3389/fphar.2011.00049 ; PubMed Central PMCID: PMC3161244. 21904528 PMC3161244

[15] Park S, Chang CY, Safi R, Liu X, Baldi R, Jasper JS, et al. ERRalpha-Regulated Lactate Metabolism Contributes to Resistance to Targeted Therapies in Breast Cancer. Cell Rep. 2016;15(2):323–35. Epub 2016/04/07. doi: 10.1016/j.celrep.2016.03.026 ; PubMed Central PMCID: PMC4833658. 27050525 PMC4833658

[16] Voukantsis D, Kahn K, Hadley M, Wilson R, Buffa FM. Modeling genotypes in their microenvironment to predict single- and multi-cellular behavior. Gigascience. 2019;8(3). Epub 2019/02/05. doi: 10.1093/gigascience/giz010 ; PubMed Central PMCID: PMC6423375. 30715320 PMC6423375

[17] Bull JA, Mech F, Quaiser T, Waters SL, Byrne HM. Mathematical modelling reveals cellular dynamics within tumour spheroids. PLoS Comput Biol. 2020;16(8):e1007961. Epub 2020/08/19. doi: 10.1371/journal.pcbi.1007961 ; PubMed Central PMCID: PMC7455028. 32810174 PMC7455028

[18] Ghaffarizadeh A, Heiland R, Friedman SH, Mumenthaler SM, Macklin P. PhysiCell: An open source physics-based cell simulator for 3-D multicellular systems. PLoS Comput Biol. 2018;14(2):e1005991. Epub 2018/02/24. doi: 10.1371/journal.pcbi.1005991 ; PubMed Central PMCID: PMC5841829. 29474446 PMC5841829

[19] Letort G, Montagud A, Stoll G, Heiland R, Barillot E, Macklin P, et al. PhysiBoSS: a multi-scale agent-based modelling framework integrating physical dimension and cell signalling. Bioinformatics. 2019;35(7):1188–96. Epub 2018/09/01. doi: 10.1093/bioinformatics/bty766 ; PubMed Central PMCID: PMC6449758. 30169736 PMC6449758

[20] Jiang Y, Pjesivac-Grbovic J, Cantrell C, Freyer JP. A multiscale model for avascular tumor growth. Biophys J. 2005;89(6):3884–94. Epub 2005/10/04. doi: 10.1529/biophysj.105.060640 ; PubMed Central PMCID: PMC1366955. 16199495 PMC1366955

[21] Sun X, Zhang L, Tan H, Bao J, Strouthos C, Zhou X. Multi-scale agent-based brain cancer modeling and prediction of TKI treatment response: incorporating EGFR signaling pathway and angiogenesis. BMC Bioinformatics. 2012;13:218. Epub 2012/09/01. doi: 10.1186/1471-2105-13-218 ; PubMed Central PMCID: PMC3487967. 22935054 PMC3487967

[22] Mao X, McManaway S, Jaiswal JK, Patel PB, Wilson WR, Hicks KO, et al. An agent-based model for drug-radiation interactions in the tumour microenvironment: Hypoxia-activated prodrug SN30000 in multicellular tumour spheroids. PLoS Comput Biol. 2018;14(10):e1006469. Epub 2018/10/26. doi: 10.1371/journal.pcbi.1006469 ; PubMed Central PMCID: PMC6218095. 30356233 PMC6218095

[23] Randles A, Wirsching HG, Dean JA, Cheng YK, Emerson S, Pattwell SS, et al. Computational modelling of perivascular-niche dynamics for the optimization of treatment schedules for glioblastoma. Nat Biomed Eng. 2021;5(4):346–59. Epub 2021/04/18. doi: 10.1038/s41551-021-00710-3 ; PubMed Central PMCID: PMC8054983. 33864039 PMC8054983

[24] Norton KA, Jin K, Popel AS. Modeling triple-negative breast cancer heterogeneity: Effects of stromal macrophages, fibroblasts and tumor vasculature. J Theor Biol. 2018;452:56–68. Epub 2018/05/12. doi: 10.1016/j.jtbi.2018.05.003 ; PubMed Central PMCID: PMC6127870. 29750999 PMC6127870

[25] Ghadiri M, Heidari M, Marashi SA, Mousavi SH. A multiscale agent-based framework integrated with a constraint-based metabolic network model of cancer for simulating avascular tumor growth. Mol Biosyst. 2017;13(9):1888–97. Epub 2017/07/25. doi: 10.1039/c7mb00050b . 28737788

[26] Roy M, Finley SD. Metabolic reprogramming dynamics in tumor spheroids: Insights from a multicellular, multiscale model. PLoS Comput Biol. 2019;15(6):e1007053. Epub 2019/06/12. doi: 10.1371/journal.pcbi.1007053 ; PubMed Central PMCID: PMC6588258. 31185009 PMC6588258

[27] Ibrahim-Hashim A, Robertson-Tessi M, Enriquez-Navas PM, Damaghi M, Balagurunathan Y, Wojtkowiak JW, et al. Defining Cancer Subpopulations by Adaptive Strategies Rather Than Molecular Properties Provides Novel Insights into Intratumoral Evolution. Cancer Res. 2017;77(9):2242–54. Epub 2017/03/03. doi: 10.1158/0008-5472.CAN-16-2844 ; PubMed Central PMCID: PMC6005351. 28249898 PMC6005351

[28] Yu JS, Bagheri N. Agent-Based Models Predict Emergent Behavior of Heterogeneous Cell Populations in Dynamic Microenvironments. Front Bioeng Biotechnol. 2020;8:249. Epub 2020/07/01. doi: 10.3389/fbioe.2020.00249 ; PubMed Central PMCID: PMC7301008. 32596213 PMC7301008

[29] Wilensky U. NetLogo. http://ccl.northwestern.edu/netlogo/. Center for Connected Learning and Computer-Based Modeling, Northwestern University, Evanston, IL. 1999.

[30] Grieco L, Calzone L, Bernard-Pierrot I, Radvanyi F, Kahn-Perles B, Thieffry D. Integrative modelling of the influence of MAPK network on cancer cell fate decision. PLoS Comput Biol. 2013;9(10):e1003286. Epub 2013/11/20. doi: 10.1371/journal.pcbi.1003286 ; PubMed Central PMCID: PMC3821540. 24250280 PMC3821540

[31] McGillen JB, Kelly CJ, Martinez-Gonzalez A, Martin NK, Gaffney EA, Maini PK, et al. Glucose-lactate metabolic cooperation in cancer: insights from a spatial mathematical model and implications for targeted therapy. J Theor Biol. 2014;361:190–203. Epub 2014/09/30. doi: 10.1016/j.jtbi.2014.09.018 . 25264268

[32] Mendoza-Juez B, Martinez-Gonzalez A, Calvo GF, Perez-Garcia VM. A mathematical model for the glucose-lactate metabolism of in vitro cancer cells. Bull Math Biol. 2012;74(5):1125–42. Epub 2011/12/23. doi: 10.1007/s11538-011-9711-z . 22190043

[33] Mendonsa AM, Na TY, Gumbiner BM. E-cadherin in contact inhibition and cancer. Oncogene. 2018;37(35):4769–80. Epub 2018/05/22. doi: 10.1038/s41388-018-0304-2 ; PubMed Central PMCID: PMC6119098. 29780167 PMC6119098

[34] Leontieva OV, Demidenko ZN, Blagosklonny MV. Contact inhibition and high cell density deactivate the mammalian target of rapamycin pathway, thus suppressing the senescence program. Proc Natl Acad Sci U S A. 2014;111(24):8832–7. Epub 2014/06/04. doi: 10.1073/pnas.1405723111 ; PubMed Central PMCID: PMC4066505. 24889617 PMC4066505

[35] Chen Y, Cairns R, Papandreou I, Koong A, Denko NC. Oxygen consumption can regulate the growth of tumors, a new perspective on the Warburg effect. PLoS One. 2009;4(9):e7033. Epub 2009/09/16. doi: 10.1371/journal.pone.0007033 ; PubMed Central PMCID: PMC2737639. 19753307 PMC2737639

[36] Romero N SPM, Kam Y, Rogers G., Dranka B Bioenergetic profiling and fuel dependencies of cancer cell lines: quantifying the impact of glycolytic and mitochondrial ATP production on cell proliferation. Agilent Technologies, Lexington, MA, 2018.

[37] Seaholm SK, Ackerman E, Wu SC. Latin hypercube sampling and the sensitivity analysis of a Monte Carlo epidemic model. Int J Biomed Comput. 1988;23(1–2):97–112. Epub 1988/10/01. doi: 10.1016/0020-7101(88)90067-0 . 3065249

[38] Marino S, Hogue IB, Ray CJ, Kirschner DE. A methodology for performing global uncertainty and sensitivity analysis in systems biology. J Theor Biol. 2008;254(1):178–96. Epub 2008/06/24. doi: 10.1016/j.jtbi.2008.04.011 ; PubMed Central PMCID: PMC2570191. 18572196 PMC2570191

[39] Vander Heiden MG, Cantley LC, Thompson CB. Understanding the Warburg effect: the metabolic requirements of cell proliferation. Science. 2009;324(5930):1029–33. Epub 2009/05/23. doi: 10.1126/science.1160809 ; PubMed Central PMCID: PMC2849637. 19460998 PMC2849637

[40] Damaghi M, West J, Robertson-Tessi M, Xu L, Ferrall-Fairbanks MC, Stewart PA, et al. The harsh microenvironment in early breast cancer selects for a Warburg phenotype. Proc Natl Acad Sci U S A. 2021;118(3). Epub 2021/01/17. doi: 10.1073/pnas.2011342118 ; PubMed Central PMCID: PMC7826394. 33452133 PMC7826394

[41] Goodwin ML, Gladden LB, Nijsten MW, Jones KB. Lactate and cancer: revisiting the warburg effect in an era of lactate shuttling. Front Nutr. 2014;1:27. Epub 2014/01/01. doi: 10.3389/fnut.2014.00027 ; PubMed Central PMCID: PMC4428352. 25988127 PMC4428352

[42] Dhawan A, Barberis A, Cheng WC, Domingo E, West C, Maughan T, et al. Guidelines for using sigQC for systematic evaluation of gene signatures. Nat Protoc. 2019;14(5):1377–400. Epub 2019/04/12. doi: 10.1038/s41596-019-0136-8 . 30971781

[43] Cerami E, Gao J, Dogrusoz U, Gross BE, Sumer SO, Aksoy BA, et al. The cBio cancer genomics portal: an open platform for exploring multidimensional cancer genomics data. Cancer Discov. 2012;2(5):401–4. Epub 2012/05/17. doi: 10.1158/2159-8290.CD-12-0095 ; PubMed Central PMCID: PMC3956037. 22588877 PMC3956037

[44] Levine AJ. p53: 800 million years of evolution and 40 years of discovery. Nat Rev Cancer. 2020;20(8):471–80. Epub 2020/05/15. doi: 10.1038/s41568-020-0262-1 . 32404993

[45] Vousden KH, Ryan KM. p53 and metabolism. Nat Rev Cancer. 2009;9(10):691–700. Epub 2009/09/18. doi: 10.1038/nrc2715 . 19759539

[46] Liu J, Zhang C, Hu W, Feng Z. Tumor suppressor p53 and metabolism. J Mol Cell Biol. 2019;11(4):284–92. Epub 2018/12/01. doi: 10.1093/jmcb/mjy070 ; PubMed Central PMCID: PMC6487777. 30500901 PMC6487777

[47] Sermeus A, Michiels C. Reciprocal influence of the p53 and the hypoxic pathways. Cell Death Dis. 2011;2:e164. Epub 2011/05/27. doi: 10.1038/cddis.2011.48 ; PubMed Central PMCID: PMC3122125. 21614094 PMC3122125

[48] Harris AL. Development of cancer metabolism as a therapeutic target: new pathways, patient studies, stratification and combination therapy. Br J Cancer. 2020;122(1):1–3. Epub 2019/12/11. doi: 10.1038/s41416-019-0666-4 ; PubMed Central PMCID: PMC6964699. 31819198 PMC6964699

[49] Lin X, Xiao Z, Chen T, Liang SH, Guo H. Glucose Metabolism on Tumor Plasticity, Diagnosis, and Treatment. Front Oncol. 2020;10:317. Epub 2020/03/27. doi: 10.3389/fonc.2020.00317 ; PubMed Central PMCID: PMC7069415. 32211335 PMC7069415

[50] Guan X, Rodriguez-Cruz V, Morris ME. Cellular Uptake of MCT1 Inhibitors AR-C155858 and AZD3965 and Their Effects on MCT-Mediated Transport of L-Lactate in Murine 4T1 Breast Tumor Cancer Cells. AAPS J. 2019;21(2):13. Epub 2019/01/09. doi: 10.1208/s12248-018-0279-5 ; PubMed Central PMCID: PMC6466617. 30617815 PMC6466617

[51] Guan X, Morris ME. In Vitro and In Vivo Efficacy of AZD3965 and Alpha-Cyano-4-Hydroxycinnamic Acid in the Murine 4T1 Breast Tumor Model. AAPS J. 2020;22(4):84. Epub 2020/06/13. doi: 10.1208/s12248-020-00466-9 ; PubMed Central PMCID: PMC8066402. 32529599 PMC8066402

[52] Beloueche-Babari M, Casals Galobart T, Delgado-Goni T, Wantuch S, Parkes HG, Tandy D, et al. Monocarboxylate transporter 1 blockade with AZD3965 inhibits lipid biosynthesis and increases tumour immune cell infiltration. Br J Cancer. 2020;122(6):895–903. Epub 2020/01/16. doi: 10.1038/s41416-019-0717-x ; PubMed Central PMCID: PMC7078321. 31937921 PMC7078321

[53] Beloueche-Babari M, Wantuch S, Casals Galobart T, Koniordou M, Parkes HG, Arunan V, et al. MCT1 Inhibitor AZD3965 Increases Mitochondrial Metabolism, Facilitating Combination Therapy and Noninvasive Magnetic Resonance Spectroscopy. Cancer Res. 2017;77(21):5913–24. Epub 2017/09/20. doi: 10.1158/0008-5472.CAN-16-2686 ; PubMed Central PMCID: PMC5669455. 28923861 PMC5669455

[54] Lamb R, Harrison H, Hulit J, Smith DL, Lisanti MP, Sotgia F. Mitochondria as new therapeutic targets for eradicating cancer stem cells: Quantitative proteomics and functional validation via MCT1/2 inhibition. Oncotarget. 2014;5(22):11029–37. Epub 2014/11/22. doi: 10.18632/oncotarget.2789 ; PubMed Central PMCID: PMC4294326. 25415228 PMC4294326

[55] Halford S, Veal GJ, Wedge SR, Payne GS, Bacon CM, Sloan P, et al. A Phase I Dose-escalation Study of AZD3965, an Oral Monocarboxylate Transporter 1 Inhibitor, in Patients with Advanced Cancer. Clin Cancer Res. 2023;29(8):1429–39. Epub 2023/01/19. doi: 10.1158/1078-0432.CCR-22-2263 ; PubMed Central PMCID: PMC7614436. 36652553 PMC7614436

[56] Reckzeh ES, Karageorgis G, Schwalfenberg M, Ceballos J, Nowacki J, Stroet MCM, et al. Inhibition of Glucose Transporters and Glutaminase Synergistically Impairs Tumor Cell Growth. Cell Chem Biol. 2019;26(9):1214–28 e25. Epub 2019/07/16. doi: 10.1016/j.chembiol.2019.06.005 . 31303578

[57] Reckzeh ES, Waldmann H. Small-Molecule Inhibition of Glucose Transporters GLUT-1-4. Chembiochem. 2020;21(1–2):45–52. Epub 2019/09/26. doi: 10.1002/cbic.201900544 ; PubMed Central PMCID: PMC7004114. 31553512 PMC7004114

[58] Collins A J., Seiler M J., Gangel M, Croll M. Applying Latin hypercube sampling to agent-based models. International Journal of Housing Markets and Analysis. 2013;6(4):422–37. doi: 10.1108/IJHMA-Jul-2012-0027

[59] Rockwell S, Dobrucki IT, Kim EY, Marrison ST, Vu VT. Hypoxia and radiation therapy: past history, ongoing research, and future promise. Curr Mol Med. 2009;9(4):442–58. Epub 2009/06/13. doi: 10.2174/156652409788167087 ; PubMed Central PMCID: PMC2752413. 19519402 PMC2752413

[60] Corbet C, Bastien E, Draoui N, Doix B, Mignion L, Jordan BF, et al. Interruption of lactate uptake by inhibiting mitochondrial pyruvate transport unravels direct antitumor and radiosensitizing effects. Nat Commun. 2018;9(1):1208. Epub 2018/03/25. doi: 10.1038/s41467-018-03525-0 ; PubMed Central PMCID: PMC5865202. 29572438 PMC5865202

[61] Bola BM, Chadwick AL, Michopoulos F, Blount KG, Telfer BA, Williams KJ, et al. Inhibition of monocarboxylate transporter-1 (MCT1) by AZD3965 enhances radiosensitivity by reducing lactate transport. Mol Cancer Ther. 2014;13(12):2805–16. Epub 2014/10/05. doi: 10.1158/1535-7163.MCT-13-1091 ; PubMed Central PMCID: PMC4258406. 25281618 PMC4258406

