# Supplementary material for: Metabolic symbiosis between oxygenated and hypoxic tumour cells: An agent-based modelling study
Source: PLoS Comput Biol. 2024 Mar 15;20(3):e1011944. doi: 10.1371/journal.pcbi.1011944 (PMC10971686; doi:10.1371/journal.pcbi.1011944)
Supplement: S1 Text — Different spatial and temporal scales of the model and how these scales interact each other are described. More details about the cell regulatory network and model parameters are given. (DOCX) [file pcbi.1011944.s001.docx]

**S1 Text. Methodology and Parameters**

**Metabolic Symbiosis between Oxygenated and Hypoxic Tumour Cells: An Agent-based Modelling Study**

Pahala Gedara Jayathilake^1,*^, Pedro Victori^1^, Clara E. Pavillet^2^, Chang Heon Lee^1^, Dimitrios Voukantsis^1^, Ana Miar^1^, Anjali Arora^1^, Adrian L. Harris^1^, Karl J. Morten^3^, Francesca M. Buffa^1,4,*^

1. Department of Oncology, Medical Sciences Division, University of Oxford, UK
2. MRC Weatherall Institute of Molecular Medicine, Radcliffe Department of Medicine, Medical Sciences Division, University of Oxford, UK
3. Nuffield Department of Women's and Reproductive Health, University of Oxford, UK
4. Department of Computing Sciences and Institute for Data Science and Analytics, Bocconi University, Milan, Italy

* [francesca.buffa@oncology.ox.ac.uk](mailto:francesca.buffa@oncology.ox.ac.uk) (FMB)

[Jayathilake.pahalagedara@oncology.ox.ac.uk](mailto:Jayathilake.pahalagedara@oncology.ox.ac.uk) (PGJ)

1. **Introduction**

The purpose of this supporting information is to provide more details about our multiscale agent-based mathematical model of tumour growth and model parameters.

**2. Overview of multi-scale agent-based mathematical model**

Our model has three different spatial scales to describe different biological processes and cell-cell and cell-microenvironment interactions. The three scales are the intra-cellular, cellular and extra-cellular scales and these scales are inter-connected each other as shown in **S1 Fig**. The intra-cellular scale describes cell-regulatory network and it decide cell phenotype based on signals it receives from the tumour microenvironment and intra-cellular molecular interactions. The cellular scale describes cell-cell and cell-micro environmental interactions and the extra-cellular scale describes spatial distribution of diffusible substances in the tumour microenvironment. Both intra-cellular and cellular scales are modelled as agent-based models while the extra-cellular scale is modelled as a continuum model. More details about each scale are given below.

- 1. **Model framework**
     1. **Intra-cellular scale: Boolean network**

The cell regulatory network is modelled at the intra-cellular scale by using an agent-based model. This is a Boolean network which contains a MAPK (Mitogen-Activated Protein Kinases) network together with glucose and lactate metabolic pathways as shown in **S2 Fig**. This is a mathematical representation of signalling and metabolic pathways involved in cellular responses to stimuli such as growth factors (e.g., Epidermal growth factor, Hepatocyte growth factor) and nutrients (e.g., Glucose, Lactate). The each node represents molecules (e.g., Proteins, Genes), cellular processes (e.g., Electron Transport Chain, Tricarboxylic Acid Cycle) and cell phenotypes (e.g. Proliferation, Apoptosis). In **S2 Fig**, the green and red lines describe positive and negative interactions between nodes, respectively. Each node has its own specific Boolean logical condition and if the condition is true the node is considered as active (i.e., 1) and otherwise inactive (i.e., 0). The vast majority of the network was taken from literature and logical conditions associated with those nodes can be found in [1, 2]. The newly added nodes to the network and associated logical conditions are explained in **Table A**. To read more about the network, our Boolean network contained in the source file, **S1 File,** can be opened in GINsim software (<http://ginsim.org>) and all the Boolean logical conditions and respective supporting evidence can be seen there. We have converted this network source file to an html file using Oxygen XML Editor to use with our multi-scale model.

This network is encapsulated inside each tumour cell and the network can obtain stimuli such as growth factors and nutrients from the extra-cellular environment through input nodes (i.e., rose coloured nodes of **S2 Fig**). An environmental stimulus can activate the respective input node if local stimulus concentration is above a certain threshold. For example, the input node, Glucose_supply, is activated if the local glucose level is above a certain threshold. Similarly, the input node, Oxygen_supply, is activated if the local oxygen level is above a certain threshold and so on (**Tables A** and **B**). The input nodes could be cell membrane receptors such as Epidermal Growth Factor Receptor – EGFR, membrane transport proteins such as Glucose Transporter – GLUT1 and stresses such as DNA damage. Upon receiving stimuli, the network can decide which metabolic pathway to use and that could be glycolysis, glucose-driven oxidative phosphorylation (OXPHOS) or lactate-driven OXPHOS. The network can also decide the cellular phenotype (i.e., blue coloured node of **S2 Fig**) which could be proliferation, growth arrest, apoptosis or necrosis.

- - 1. **Cellular scale: Cellular automaton method**

At cellular scale, individual cell behaviour is modelled using the Cellular Automaton (CA) method which is a lattice-based agent-based modelling approach. Each cell is represented by a spherical particle and the particle can move on a Cartesian grid according to CA rules [2]. This model describes cell-cell and cell-microenvironmental interactions.

A proliferate cell which has completed the cell division time can only divide if there is an empty space around the cell, otherwise it may remain in contact inhibition or quiescence state for a certain period of time before checking environmental conditions again to find its new phenotype. A cell turns to apoptosis would be removed from the simulation immediately. Growth arrest cells would consume nutrients at half of the normal rate. The necrotic cells would not consume or produce any substance other than exist in in the tumour. Cells can interact with the tumour microenvironment though consumption and production of diffusible substances such as nutrients and growth factors.

- - 1. **Extra-cellular scale: Tumour microenvironment**

At this scale, following diffusion reaction equation describes the distribution of diffusible substances in the tumour microenvironment.

$\frac{\partial C_{S}}{\partial t}=D_{S}\nabla^{2}C_{S}+R_{S}\rho_{cell,S}$ S1

where *C*, *D* and *R* are the substance concentration, its diffusion coefficient and its rate of consumption or production, respectively. $\rho_{cell,S}$represents the density of cells which consume or produce substance *S*. For example, the growth factor TGFA is produced by only hypoxic tumour cells as modelled in the cell regulatory network and therefore only the cells with active TGFA node of the network are included when calculating respective cell density. The diffusible substances include oxygen, glucose, lactate and growth factors. The growth factors are TGFA, HGF and FGF for activation of EGFR, cMET and FGFR, respectively. More details about the substance production and consumption rates, $R_{S}$ , have been discussed in the main text.

The diffusion-reaction equation is solved on a Cartesian grid by using Forward Time Centered Space (FTCS) finite difference method.

- 1. **Coupling between scales**

As shown in **S1 Fig**, three different spatial scales of this tumour model are described by three sub-models using an equation-based (i.e., diffusion-reaction equation) model and two agent-based models (i.e., Cellular Automaton model and Boolean network). Time scales of different processes have also been separated as shown in **S3 Fig**. At each *T_Network_*, one randomly selected node of the regulatory network will be updated based on existing Boolean conditions of neighbouring nodes (i.e., 0 or 1) and its own logical condition. The cell phenotype is updated at an interval of *T_Phenotype_* (red ticks) based on the outputs from the regulatory network for given stimuli. The distribution of diffusible substances in the tumour microenvironment are updated at an interval of *T_Diffusion_* (green ticks) by solving the diffusion-reaction equation for steady state at each green tick. A tumour cell would not divide until it completes its cell division time of *T_Division_*.

- 1. **Stochasticity of the model**

Stochasticity appears in the model in following processes.

1. Cell regulatory network update: The regulatory network is initialized randomly, which means each node is initialized with 0 or 1 randomly. Then at each time step, a randomly selected node of the regulatory network will be updated based on its Boolean logical condition.
2. Initial cell distribution: Different types of cancer cells such as different mutants as set by the user are placed in the tumour microenvironment randomly to make the initial tumour spheroid.
3. Cell division: When a cell is divided, the new cell is located next to the mother cell in a randomly selected direction.
   1. **Software and programming language**

The model has been originally implemented on Netlogo as reported in [2]. The present symbiosis model was implemented on the same modelling platform. The Netlogo model and its dependent files used for this study have been stored in GitHub at <https://github.com/CBigOxf/jayathilake2022> . The Netlogo model file can be run with dependent files given in our repository. More instructions about how to choose parameters are given at the top of the NetLogo code. The model output data were analysed and visualized using R.

1. **Model parameters, experimental methods, and clinical data**
   1. **Model parameters**

The model parameters are shown in **Table C**.

- 1. **Normoxia/hypoxia experiments for breast cancer cell lines**

Hypoxic and normoxic environments were created at 2% and 20% of oxygen, respectively (**S4 Fig**).

- 1. **cBioPortal**

We checked the mRNA expression levels of genes involved in our network model using the breast cancer samples of TCGA PanCancer Atlas (The Cancer Genome Atlas, <https://www.cancer.gov/tcga/>) using the cBioPortal online tool (https://www.cbioportal.org). The mRNA expression levels are given with relative to normal samples (**S5 Fig**).

- 1. **sigQC**

The R package sigQC allows us to check multiple gene signature sets across multiple gene expression datasets ([https://CRAN.R-project.org/package=sigQC](https://cran.r-project.org/package=sigQC)). We checked our signature genes in TCGA and CCLE breast cancer (Cancer Cell Line Encyclopedia, <https://sites.broadinstitute.org/ccle/>) datasets (**S6 Fig**).

**Table A.** Metabolic molecules and regulatory nodes implemented in regulatory network: In the Boolean expressions, &, |, and ! represent AND, OR, and NOT logical conditions, respectively. Only the newly added logical conditions are given here, and the rest of the conditions have been reported in [1]. S1 File can be opened in GINsim software (<http://ginsim.org>) and all the Boolean logical conditions and respective supporting evidence can be seen there.

| **Molecule (node)** | **Logical condition** | **Reference** |
| --- | --- | --- |
| GLUT1: Glucose Transporter 1 | (HIF1 \| !p53 \| MYC) & !GLUT1I | [3-7] |
| G6P: Glucose 6-Phosphate | Cell_Glucose & Glucose_supply | - |
| F6P: Fructose 6-Phosphate | G6P | [8] |
| F16BP: Fructose 1,6-Bisphosphate | F6P | [8] |
| GA3P: Glyceraldehyde 3-Phosphate | F16BP | [8] |
| BPG: 1,3-Bisphosphoglycerate | GA3P | [8] |
| PG3: 3-Phosphoglycerate | BPG | [8] |
| PG2: 2-Phosphoglycerate | PG3 | [8] |
| PEP: Phosphoenolpyruvate | PG2 | [8] |
| AcetylCoA: Acetyl Coenzyme A | Pyruvate & PDH | [8] |
| TCA: Tricarboxylic Acid Cycle | AcetylCoA | [9] |
| ETC: Electron Transport Chain | TCA & Oxygen_supply | [9] |
| mitoATP: mitochondrial ATP | ETC | [9] |
| LDHA: Lactate Dehydrogenase A | HIF1 & MYC | [6, 7] |
| LDHB: Lactate Dehydrogenase B | MCT1 | [10] |
| PDH: Pyruvate Dehydrogenas | !PDK1 \| p53 \| LDHB | [11-14] |
| glycoATP: glycolytic ATP | PEP & !LDHB | [10] |
| MCT1: Monocarboxylate transporter 1 | Oxygen_supply & MCT1_stimulus & !MCT1I | [10] |
| MCT4: Monocarboxylate transporter 4 | Cell_Lactate & !MCT1 | [10] |
| GLUT1I: GLUT1 Inhibitor | GLUT1 Drug > threshold | - |
| MCT1I: MCT1 Inhibitor | MCT1 Drug > threshold | - |
| cMET | cMET_stimulus & !cMETI | - |
| GRB2 | EGFR \| cMET \| FRS2 \| TGFBR | KEGG networks |
| PDK | PI3K \| HIF1 \| MYC | [6, 7] |

**Table B.** Input nodes and corresponding stimulus of the regulatory network: If the stimulus concentration around a tumour cell is beyond a certain threshold the respective input node is activated for that particular cell. These threshold values are given in Table C.

| **Input node** | **Stimulus** | **Used in the present study** |
| --- | --- | --- |
| Oxygen_supply | Oxygen | Yes |
| Glucose_supply | Glucose | Yes |
| EGFR_stimulus | TGFA | Yes |
| cMET_stimulus | HGF | Yes |
| FGFR_stimulus | FGF | No |
| TGFBR_stimulus | TGFB | No |
| MCT1_stimulus | Lactate | Yes |
| DNA_damage | Factors causing DNA damage | No |
| EGFRI | EGFRD (EGFR Drug) | No |
| cMETI | cMETD (cMET Drug) | No |
| FGFRI | FGFRD (FGFR Drug) | No |
| GLUT1I | GLUT1D (GLUT1 Drug) | Yes |
| MCT1I | MCT1D (MCT1 Drug) | Yes |
| MCT4I | MCT4D (MCT4 Drug) | No |
| Growth_inhibitor | GI (Growth Inhibiting factors) | No |

**Table C.** Model parameters: If there is no reference for a parameter, the value is chosen such that the model work smoothly and the value is also close to its typical order of magnitude.

| **Parameter** | | **Value** | **Reference** | **Remarks** |
| --- | --- | --- | --- | --- |
| Oxygen | Diffusion coefficient | 1.0 × 10^-9^ m^2^/s | [15] |  |
|  | Consumption rate | 3.00 × 10^-17^ mol/s/cell | [16] |  |
|  | Half-saturation coefficient | 0.005 mM | [17] |  |
|  | Boundary level | 0.07 mM | [18] |  |
|  | Necrosis level | 0.011 mM (1% P_O2_) | [19] | Pathological hypoxia |
|  | Activation threshold | 0.022 mM (2% P_O2_) | [19] | Physiological hypoxia |
| Glucose | Diffusion coefficient | 6.7 × 10^-11^ m^2^/s | [20] |  |
|  | Half-saturation coefficient | 0.04 mM | [17] |  |
|  | Boundary level | 5.0 mM | [18] |  |
|  | Necrosis level | 3.9 mM | Assumed | A wide range of values seen in the literature. 0.06 mM [21], 0.15 µM [22]. 2.27 mM [23], 8mM [20]. |
|  | Activation threshold | 4.0 mM | [24] | [24] has used 5 mM, but we reduce it to 4 mM such that we see tumour growth at 5mM which is commonly seen in literature. |
| Lactate | Diffusion coefficient | 6.7 × 10^-11^ m^2^/s | [21] |  |
|  | Half-saturation coefficient | 0.04 mM | Assumed | Assuming same as for glucose |
|  | Boundary level | 1 mM | Assumed | Low lactate level far from tumour |
|  | Activation threshold | 1.5 mM | [25] |  |
| TGFA  (*Growth factor of EGFR*) | Diffusion coefficient | 5.18 × 10^-11^ m^2^/s | [20, 26] |  |
|  | Consumption rate | 2.00 × 10^-17^ m^3^/s/cell | Assumed | Chosen such that reasonable TGFa amount is maintained in the medium. |
|  | Production rate | 2.00 × 10^-20^ mol/s/cell | Assumed | Chosen such that reasonable TGFa amount is produced which is in nM scale. 4.00 × 10^-25^ [20] |
|  | Activation threshold | 1.0 nM | Assumed | Typical TGFa concentration of tissues is in the order of nM [27] |
|  | Boundary level | 0.0 | [2] |  |
| HGF  (*Growth factor of*  *cMET*) | Diffusion coefficient | 8.50 × 10^-11^ m^2^/s | [28] |  |
|  | Consumption rate | 2.00 × 10^-18^ m^3^/s/cell | Assumed | Chosen such that we can see HGF-induced cMET activation in tumour. |
|  | Production rate | 0.0 |  | HGF is externally provided. |
|  | Activation threshold | 1 nM | Assumed | Typical HGF concentration is in order of nM [29] |
|  | Boundary level | 2 nM | Assumed | Chosen such that we can see HGF-induced cMET activation in tumour. |
| H+ | Diffusion coefficient | 1.0 × 10^-9^ m^2^/s | [17] |  |
|  | Boundary level | 40 nM | Assumed | Acidity decreases as moving away from tumour. |
| MCT1i  GLUT1i  (*MCT1 and GLUT1 inhibitors*) | Diffusion coefficient | 2.2 × 10^-10^ m^2^/s | Assumed | Assuming similar to 2.5 × 10^-10^ of Gefitinib/EMD [28] |
|  | Consumption rate | 4.00 × 10^-17^ m^3^/s/cell | Assumed | Assuming similar to TGFA |
|  | Activation threshold | IC50 (17 nM for MCT1i and 4nM for GLUT1i) |  | Typical values [30]  MCT1i=AZD3965 drug  GLUT1i=Glupin drug |
|  | Boundary level | Variable (See the text) |  |  |
| Spatial & temporal parameters | Computational domain | 0.8 × 0.8 mm^2^ | - | A reasonable size for computation |
|  | Cell diameter | 20 µm | [31] |  |
|  | Cell division time | 1000 steps = 1.3 days | [32] |  |
|  | Diffusion time | 250 steps | - |  |
|  | Cell phenotype time | 100 steps | [2] |  |

1. **Supplementary results**

**S4-S6 Figs** and **S7-S17 Figs** show our experimental/TCGA/CCLE data and simulation data, respectively.

**References**

1. Grieco L, Calzone L, Bernard-Pierrot I, Radvanyi F, Kahn-Perles B, Thieffry D. Integrative modelling of the influence of MAPK network on cancer cell fate decision. PLoS Comput Biol. 2013;9(10):e1003286. Epub 2013/11/20. doi: 10.1371/journal.pcbi.1003286. PubMed PMID: 24250280; PubMed Central PMCID: PMCPMC3821540.

2. Voukantsis D, Kahn K, Hadley M, Wilson R, Buffa FM. Modeling genotypes in their microenvironment to predict single- and multi-cellular behavior. Gigascience. 2019;8(3). Epub 2019/02/05. doi: 10.1093/gigascience/giz010. PubMed PMID: 30715320; PubMed Central PMCID: PMCPMC6423375.

3. Gatenby RA, Smallbone K, Maini PK, Rose F, Averill J, Nagle RB, et al. Cellular adaptations to hypoxia and acidosis during somatic evolution of breast cancer. Br J Cancer. 2007;97(5):646-53. Epub 2007/08/10. doi: 10.1038/sj.bjc.6603922. PubMed PMID: 17687336; PubMed Central PMCID: PMCPMC2360372.

4. Airley R, Loncaster J, Davidson S, Bromley M, Roberts S, Patterson A, et al. Glucose transporter glut-1 expression correlates with tumor hypoxia and predicts metastasis-free survival in advanced carcinoma of the cervix. Clin Cancer Res. 2001;7(4):928-34. Epub 2001/04/20. PubMed PMID: 11309343.

5. Liu J, Zhang C, Hu W, Feng Z. Tumor suppressor p53 and metabolism. J Mol Cell Biol. 2019;11(4):284-92. Epub 2018/12/01. doi: 10.1093/jmcb/mjy070. PubMed PMID: 30500901; PubMed Central PMCID: PMCPMC6487777.

6. Hsieh AL, Walton ZE, Altman BJ, Stine ZE, Dang CV. MYC and metabolism on the path to cancer. Semin Cell Dev Biol. 2015;43:11-21. Epub 2015/08/19. doi: 10.1016/j.semcdb.2015.08.003. PubMed PMID: 26277543; PubMed Central PMCID: PMCPMC4818970.

7. Dang CV, Kim JW, Gao P, Yustein J. The interplay between MYC and HIF in cancer. Nat Rev Cancer. 2008;8(1):51-6. Epub 2007/11/30. doi: 10.1038/nrc2274. PubMed PMID: 18046334.

8. Yeung SJ, Pan J, Lee MH. Roles of p53, MYC and HIF-1 in regulating glycolysis - the seventh hallmark of cancer. Cell Mol Life Sci. 2008;65(24):3981-99. Epub 2008/09/04. doi: 10.1007/s00018-008-8224-x. PubMed PMID: 18766298.

9. Denko NC. Hypoxia, HIF1 and glucose metabolism in the solid tumour. Nat Rev Cancer. 2008;8(9):705-13. Epub 2009/01/15. doi: 10.1038/nrc2468. PubMed PMID: 19143055.

10. Semenza GL. Tumor metabolism: cancer cells give and take lactate. J Clin Invest. 2008;118(12):3835-7. Epub 2008/11/27. doi: 10.1172/JCI37373. PubMed PMID: 19033652; PubMed Central PMCID: PMCPMC2582934.

11. Liang Y, Liu J, Feng Z. The regulation of cellular metabolism by tumor suppressor p53. Cell Biosci. 2013;3(1):9. Epub 2013/02/08. doi: 10.1186/2045-3701-3-9. PubMed PMID: 23388203; PubMed Central PMCID: PMCPMC3573943.

12. Hong SM, Lee YK, Park I, Kwon SM, Min S, Yoon G. Lactic acidosis caused by repressed lactate dehydrogenase subunit B expression down-regulates mitochondrial oxidative phosphorylation via the pyruvate dehydrogenase (PDH)-PDH kinase axis. J Biol Chem. 2019;294(19):7810-20. Epub 2019/03/30. doi: 10.1074/jbc.RA118.006095. PubMed PMID: 30923124; PubMed Central PMCID: PMCPMC6514637.

13. Sharma G, Wu CY, Wynn RM, Gui W, Malloy CR, Sherry AD, et al. Real-time hyperpolarized (13)C magnetic resonance detects increased pyruvate oxidation in pyruvate dehydrogenase kinase 2/4-double knockout mouse livers. Sci Rep. 2019;9(1):16480. Epub 2019/11/13. doi: 10.1038/s41598-019-52952-6. PubMed PMID: 31712597; PubMed Central PMCID: PMCPMC6848094.

14. Tennant DA, Duran RV, Gottlieb E. Targeting metabolic transformation for cancer therapy. Nat Rev Cancer. 2010;10(4):267-77. Epub 2010/03/20. doi: 10.1038/nrc2817. PubMed PMID: 20300106.

15. Popel AS. Theory of oxygen transport to tissue. Crit Rev Biomed Eng. 1989;17(3):257-321. Epub 1989/01/01. PubMed PMID: 2673661; PubMed Central PMCID: PMCPMC5445261.

16. Wagner BA, Venkataraman S, Buettner GR. The rate of oxygen utilization by cells. Free Radic Biol Med. 2011;51(3):700-12. Epub 2011/06/15. doi: 10.1016/j.freeradbiomed.2011.05.024. PubMed PMID: 21664270; PubMed Central PMCID: PMCPMC3147247.

17. Shamsi M, Saghafian M, Dejam M, Sanati-Nezhad A. Mathematical Modeling of the Function of Warburg Effect in Tumor Microenvironment. Sci Rep. 2018;8(1):8903. Epub 2018/06/13. doi: 10.1038/s41598-018-27303-6. PubMed PMID: 29891989; PubMed Central PMCID: PMCPMC5995918.

18. Ibrahim-Hashim A, Robertson-Tessi M, Enriquez-Navas PM, Damaghi M, Balagurunathan Y, Wojtkowiak JW, et al. Defining Cancer Subpopulations by Adaptive Strategies Rather Than Molecular Properties Provides Novel Insights into Intratumoral Evolution. Cancer Res. 2017;77(9):2242-54. Epub 2017/03/03. doi: 10.1158/0008-5472.CAN-16-2844. PubMed PMID: 28249898; PubMed Central PMCID: PMCPMC6005351.

19. McKeown SR. Defining normoxia, physoxia and hypoxia in tumours-implications for treatment response. Br J Radiol. 2014;87(1035):20130676. Epub 2014/03/05. doi: 10.1259/bjr.20130676. PubMed PMID: 24588669; PubMed Central PMCID: PMCPMC4064601.

20. Sun X, Zhang L, Tan H, Bao J, Strouthos C, Zhou X. Multi-scale agent-based brain cancer modeling and prediction of TKI treatment response: incorporating EGFR signaling pathway and angiogenesis. BMC Bioinformatics. 2012;13:218. Epub 2012/09/01. doi: 10.1186/1471-2105-13-218. PubMed PMID: 22935054; PubMed Central PMCID: PMCPMC3487967.

21. Jiang Y, Pjesivac-Grbovic J, Cantrell C, Freyer JP. A multiscale model for avascular tumor growth. Biophys J. 2005;89(6):3884-94. Epub 2005/10/04. doi: 10.1529/biophysj.105.060640. PubMed PMID: 16199495; PubMed Central PMCID: PMCPMC1366955.

22. Mao X, McManaway S, Jaiswal JK, Patel PB, Wilson WR, Hicks KO, et al. An agent-based model for drug-radiation interactions in the tumour microenvironment: Hypoxia-activated prodrug SN30000 in multicellular tumour spheroids. PLoS Comput Biol. 2018;14(10):e1006469. Epub 2018/10/26. doi: 10.1371/journal.pcbi.1006469. PubMed PMID: 30356233; PubMed Central PMCID: PMCPMC6218095.

23. Schaller G, Meyer-Hermann M. Multicellular tumor spheroid in an off-lattice Voronoi-Delaunay cell model. Phys Rev E Stat Nonlin Soft Matter Phys. 2005;71(5 Pt 1):051910. Epub 2005/08/11. doi: 10.1103/PhysRevE.71.051910. PubMed PMID: 16089574.

24. Cleri F. Agent-based model of multicellular tumor spheroid evolution including cell metabolism. Eur Phys J E Soft Matter. 2019;42(8):112. Epub 2019/08/29. doi: 10.1140/epje/i2019-11878-7. PubMed PMID: 31456065.

25. Mendoza-Juez B, Martinez-Gonzalez A, Calvo GF, Perez-Garcia VM. A mathematical model for the glucose-lactate metabolism of in vitro cancer cells. Bull Math Biol. 2012;74(5):1125-42. Epub 2011/12/23. doi: 10.1007/s11538-011-9711-z. PubMed PMID: 22190043.

26. Zangooei MH, Habibi J. Hybrid multiscale modeling and prediction of cancer cell behavior. PLoS One. 2017;12(8):e0183810. Epub 2017/08/29. doi: 10.1371/journal.pone.0183810. PubMed PMID: 28846712; PubMed Central PMCID: PMCPMC5573302.

27. Bouchnita A, Hellander S, Hellander A. A 3D Multiscale Model to Explore the Role of EGFR Overexpression in Tumourigenesis. Bull Math Biol. 2019;81(7):2323-44. Epub 2019/04/25. doi: 10.1007/s11538-019-00607-y. PubMed PMID: 31016574; PubMed Central PMCID: PMCPMC6612322.

28. Son KJ, Gheibi P, Stybayeva G, Rahimian A, Revzin A. Detecting cell-secreted growth factors in microfluidic devices using bead-based biosensors. Microsyst Nanoeng. 2017;3. Epub 2017/01/01. doi: 10.1038/micronano.2017.25. PubMed PMID: 29963323; PubMed Central PMCID: PMCPMC6023413.

29. Mueller KL, Madden JM, Zoratti GL, Kuperwasser C, List K, Boerner JL. Fibroblast-secreted hepatocyte growth factor mediates epidermal growth factor receptor tyrosine kinase inhibitor resistance in triple-negative breast cancers through paracrine activation of Met. Breast Cancer Res. 2012;14(4):R104. Epub 2012/07/14. doi: 10.1186/bcr3224. PubMed PMID: 22788954; PubMed Central PMCID: PMCPMC3680928.

30. Lamb R, Harrison H, Hulit J, Smith DL, Lisanti MP, Sotgia F. Mitochondria as new therapeutic targets for eradicating cancer stem cells: Quantitative proteomics and functional validation via MCT1/2 inhibition. Oncotarget. 2014;5(22):11029-37. Epub 2014/11/22. doi: 10.18632/oncotarget.2789. PubMed PMID: 25415228; PubMed Central PMCID: PMCPMC4294326.

31. Norton KA, Jin K, Popel AS. Modeling triple-negative breast cancer heterogeneity: Effects of stromal macrophages, fibroblasts and tumor vasculature. J Theor Biol. 2018;452:56-68. Epub 2018/05/12. doi: 10.1016/j.jtbi.2018.05.003. PubMed PMID: 29750999; PubMed Central PMCID: PMCPMC6127870.

32. Sweeney KJ, Swarbrick A, Sutherland RL, Musgrove EA. Lack of relationship between CDK activity and G1 cyclin expression in breast cancer cells. Oncogene. 1998;16(22):2865-78. Epub 1998/07/22. doi: 10.1038/sj.onc.1201814. PubMed PMID: 9671407.
