# Supplementary figures and images for: Metabolic symbiosis between oxygenated and hypoxic tumour cells: An agent-based modelling study
Source: PLoS Comput Biol. 2024 Mar 15;20(3):e1011944. doi: 10.1371/journal.pcbi.1011944 (PMC10971686; doi:10.1371/journal.pcbi.1011944)

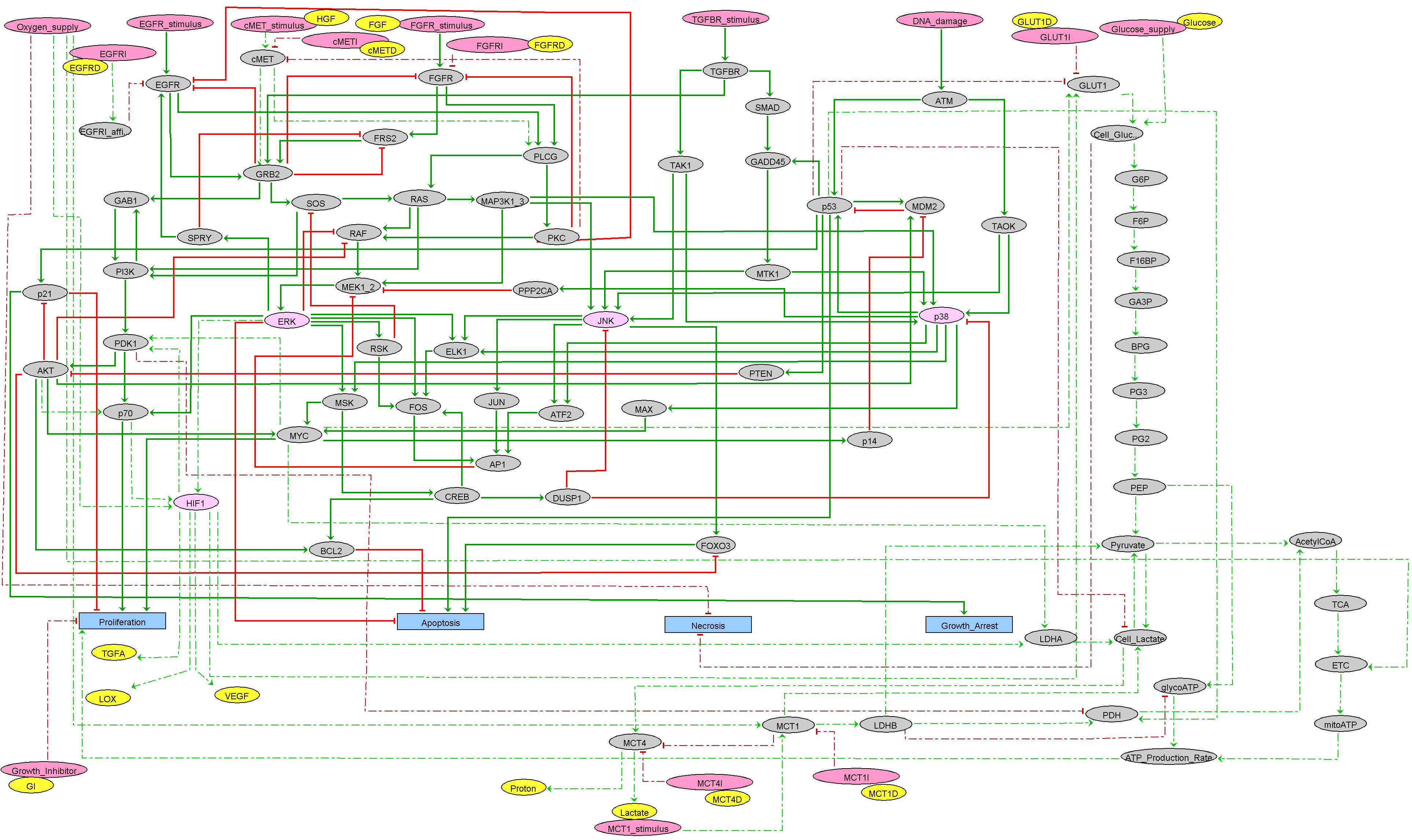

Supplement: S2 File — A high resolution image of the network. (PNG) [file pcbi.1011944.s004.png]
