# Supplementary material for: Metabolic symbiosis between oxygenated and hypoxic tumour cells: An agent-based modelling study
Source: PLoS Comput Biol. 2024 Mar 15;20(3):e1011944. doi: 10.1371/journal.pcbi.1011944 (PMC10971686; doi:10.1371/journal.pcbi.1011944)
Supplement: S1 Fig — The extra-cellular scale, an equation-based model, describes distributions of diffusible substances in the tumour microenvironment. The cellular scale, an agent-based model which is a cellular automaton model, describes cell-cell and cell-microenvironmental interactions. The intra-cellular scale, an agent-based model which is a Boolean network, describes subcellular molecular interactions. The three scales are coupled each other and information are shared between them. (DOCX) [file pcbi.1011944.s005.docx]

# **S1 Fig**


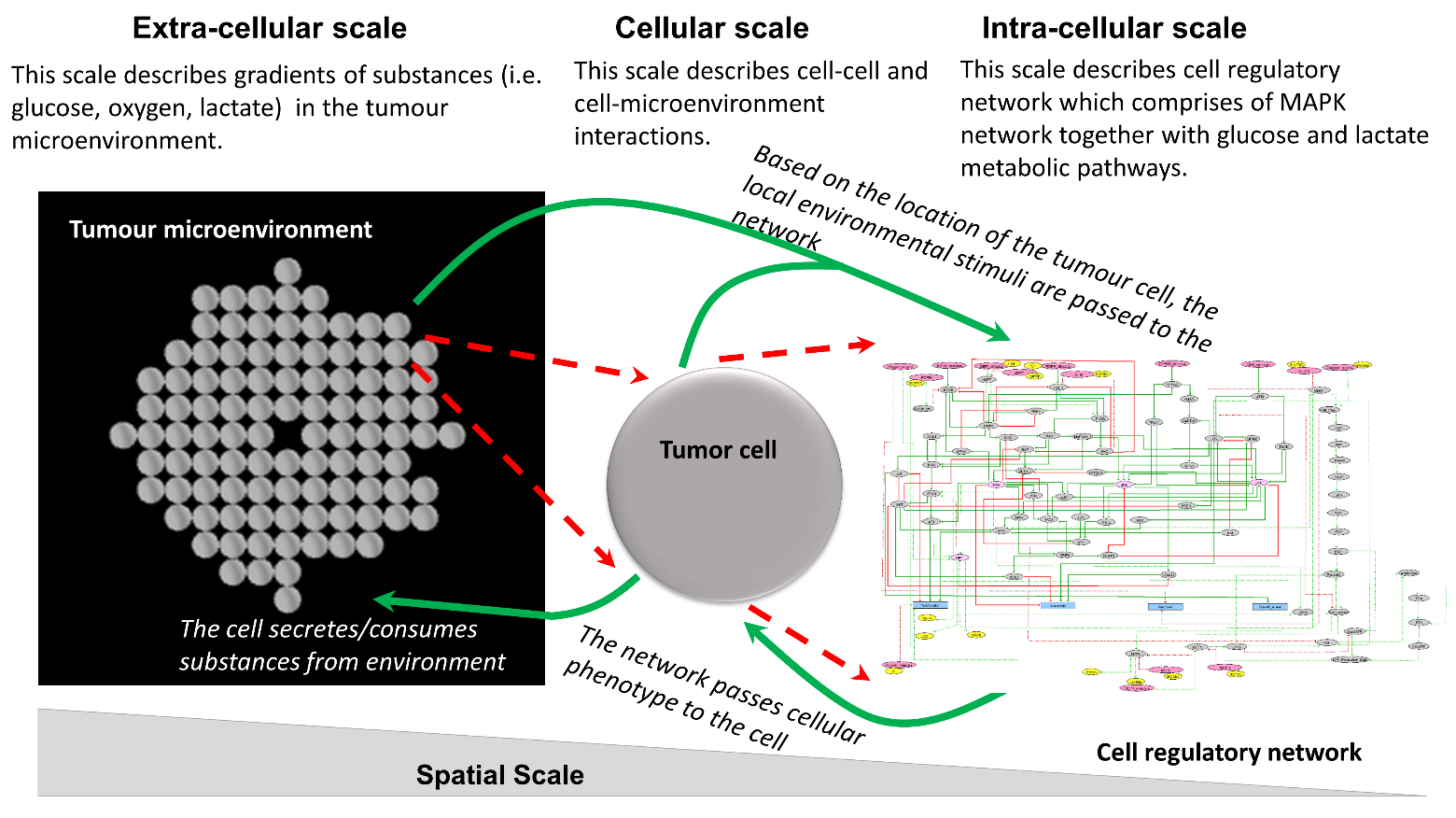


**S1 Fig. Multi-scale modelling framework:** The extra-cellular scale, an equation-based model, describes distributions of diffusible substances in the tumour microenvironment. The cellular scale, an agent-based model which is a cellular automaton model, describes cell-cell and cell-microenvironmental interactions. The intra-cellular scale, an agent-based model which is a Boolean network, describes subcellular molecular interactions. The three scales are coupled each other and information are shared between them.
