# Supplementary material for: Metabolic symbiosis between oxygenated and hypoxic tumour cells: An agent-based modelling study
Source: PLoS Comput Biol. 2024 Mar 15;20(3):e1011944. doi: 10.1371/journal.pcbi.1011944 (PMC10971686; doi:10.1371/journal.pcbi.1011944)
Supplement: S2 Fig — The modified MAPK network decides the cell phenotype based on inputs obtained from the microenvironment. Oxygen_supply, Glucose_supply, EGFR_stimulus, cMET_stimulus, FGFR_stimulus, TGFBR_stimulus, DNA_damage and Growth_inhibitor are inputs to the network. The inhibitor nodes (EGFRI, cMETI, FGFRI, GLUT1I, MCT1I, and MCT4I) are also inputs. The inhibitor nodes are activated by respective drugs (e.g. GLUT1I is activated by GLUT1D, MCT1I is activated by MCT1D and so on). The Boolean network is updated asynchronously, and cell phenotypical outputs are calculated. The outputs are Proliferation, Apoptosis, Necrosis and Growth_Arrest. The yellow-colored nodes are diffusible substances. The newly added interactions to the original MAPK network taken from [30] are shown in dotted lines. The solid and dotted green lines are positive interactions and solid and dotted red lines are negative interactions. More details about Boolean logical conditions at each node are given in [16,30] and Tables A and B in S1 Text. A high-resolution image of this network is available in S1 and S2 Files. (DOCX) [file pcbi.1011944.s006.docx]

# **S2 Fig**


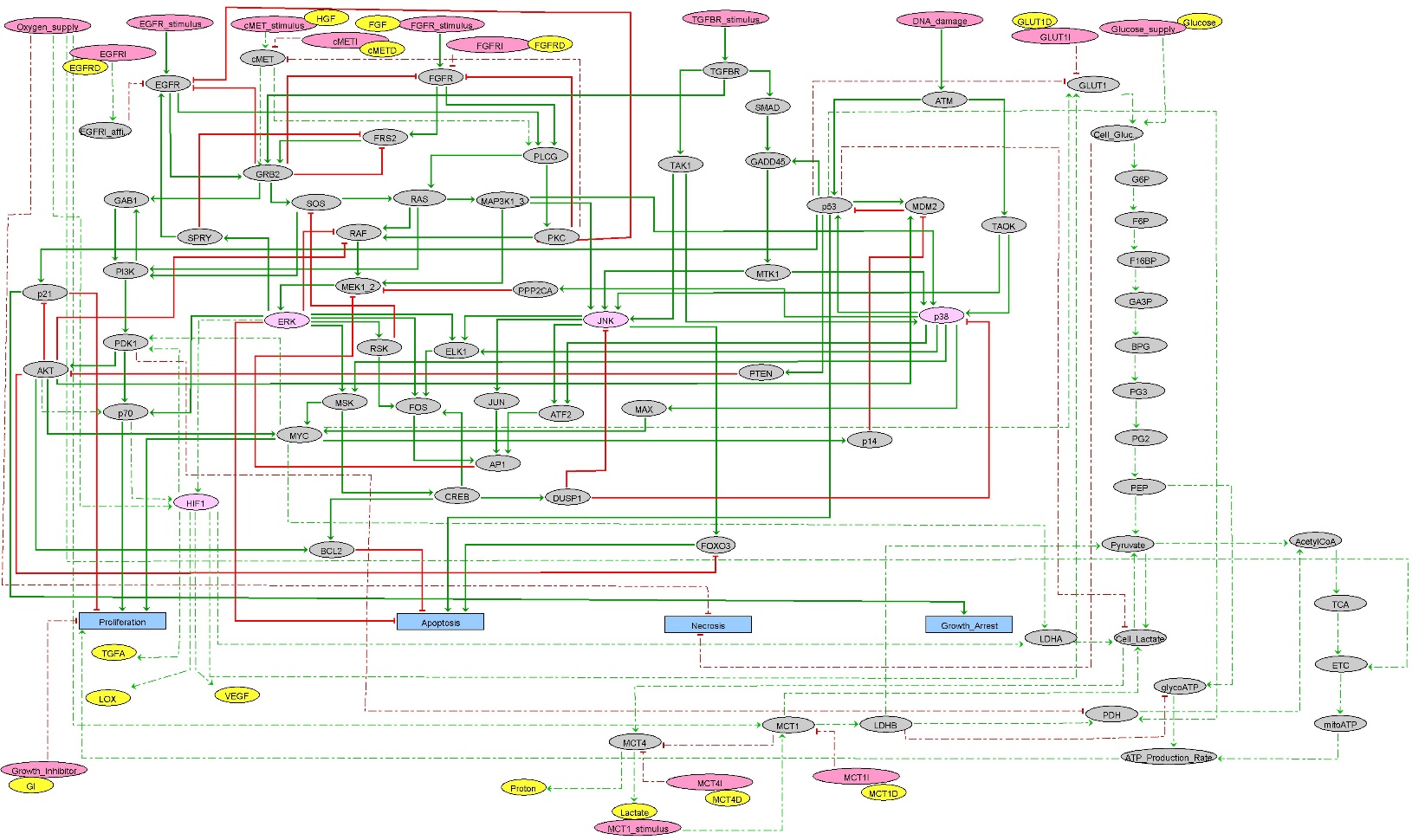


**S2 Fig. The cell regulatory network of our model (microC model):** The modified MAPK network decides the cell phenotype based on inputs obtained from the microenvironment. Oxygen_supply, Glucose_supply, EGFR_stimulus, cMET_stimulus, FGFR_stimulus, TGFBR_stimulus, DNA_damage and Growth_inhibitor are inputs to the network. The inhibitor nodes (EGFRI, cMETI, FGFRI, GLUT1I, MCT1I, and MCT4I) are also inputs. The inhibitor nodes are activated by respective drugs (e.g. GLUT1I is activated by GLUT1D, MCT1I is activated by MCT1D and so on). The Boolean network is updated asynchronously, and cell phenotypical outputs are calculated. The outputs are Proliferation, Apoptosis, Necrosis and Growth_Arrest. The yellow-colored nodes are diffusible substances. The newly added interactions to the original MAPK network taken from the literature (Ref [1] in S1 Text) are shown in dotted lines. The solid and dotted green lines are positive interactions and solid and dotted red lines are negative interactions. More details about Boolean logical conditions at each node are given in Ref [1, 2] in S1 Text and Tables A-B. A high-resolution image of this network is available in S1 and S2 Files.
