# Supplementary material for: Metabolic symbiosis between oxygenated and hypoxic tumour cells: An agent-based modelling study
Source: PLoS Comput Biol. 2024 Mar 15;20(3):e1011944. doi: 10.1371/journal.pcbi.1011944 (PMC10971686; doi:10.1371/journal.pcbi.1011944)
Supplement: S3 Fig — The smallest time step is defined as the time for updating one node of the regulatory network (TNetwork). Cell phenotype and diffusion fields are updated at red (TPhynotypes) and green (TDifusion) ticks, respectively. A cell which is older than the cell division time (TDivision) can divide if its phenotype is Proliferation. (DOCX) [file pcbi.1011944.s007.docx]

# **S3 Fig**


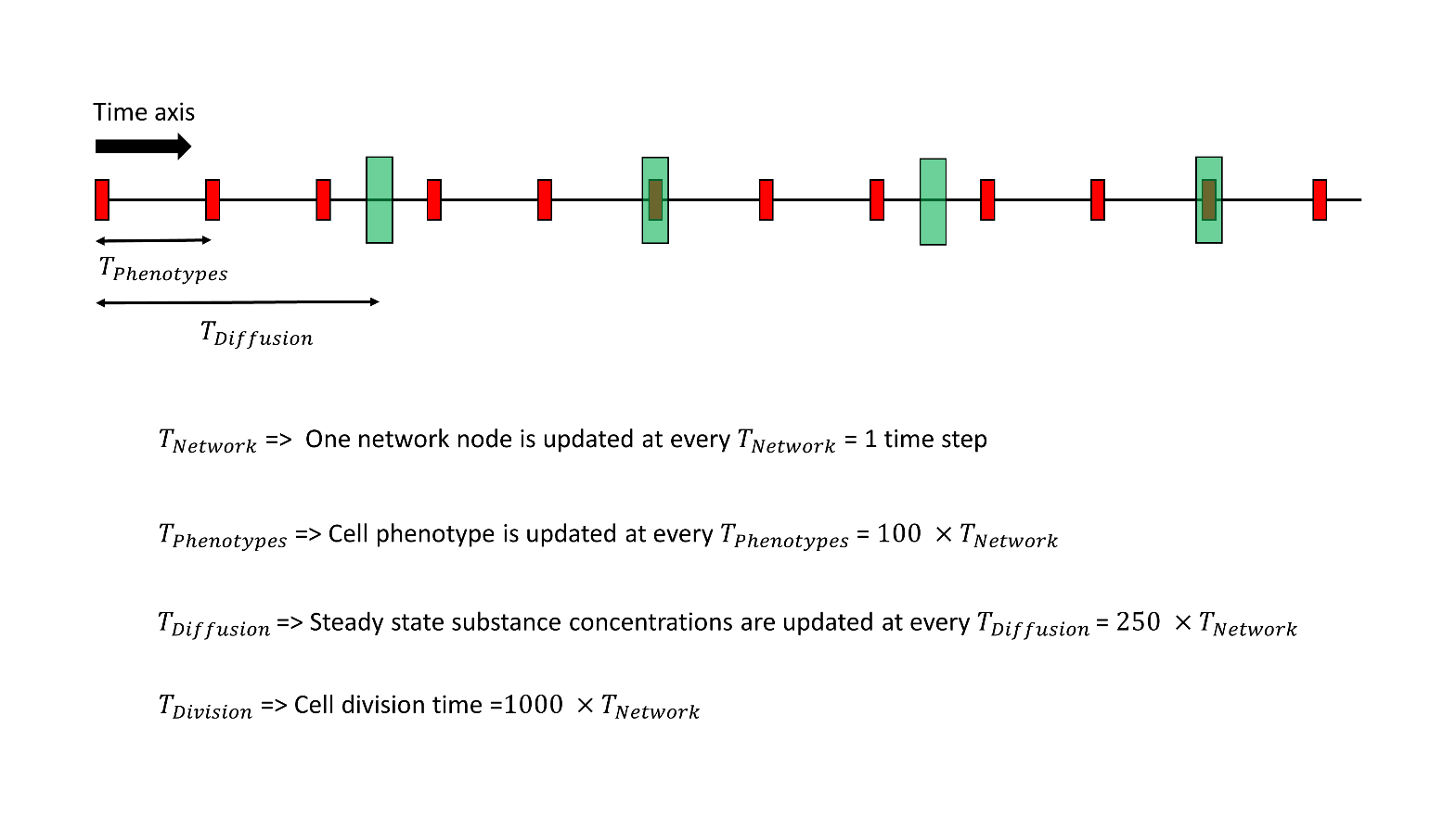


**S3 Fig. Different time scales are used for different processes:** The smallest time step is defined as the time for updating one node of the regulatory network (*T_Network_*). Cell phenotype and diffusion fields are updated at red (*T_Phynotypes_*) and green (*T_Difusion_*) ticks, respectively. A cell which is older than the cell division time (*T_Division_*) can divide if its phenotype is Proliferation.
