# Supplementary material for: Metabolic symbiosis between oxygenated and hypoxic tumour cells: An agent-based modelling study
Source: PLoS Comput Biol. 2024 Mar 15;20(3):e1011944. doi: 10.1371/journal.pcbi.1011944 (PMC10971686; doi:10.1371/journal.pcbi.1011944)
Supplement: S4 Fig — (A). Expression of our network genes of some Triple Negative Breast Cancer (TNBC) and non-TNBC cell lines under normoxia (N) and hypoxia (H). The unit is log2 (FPKM+2). (B). The gene expressions (EXP) at hypoxia are normalized by respective expressions at normoxia (log2 (EXP at hypoxia/ EXP at normoxia)). The data used to produce these Figs are given in S1 Chart. (DOCX) [file pcbi.1011944.s008.docx]

# **S4 Fig**

**A**

**B**

**S4 Fig:** **(A)**. Expression of our network genes of some Triple Negative Breast Cancer (TNBC) and non-TNBC cell lines under normoxia (N) and hypoxia (H). The unit is log2 (FPKM+2). **(B)**. The gene expressions (EXP) at hypoxia are normalized by respective expressions at normoxia (log2 (EXP at hypoxia/ EXP at normoxia)). The data used to produce these Figs are given in S1 Chart.
