# Supplementary material for: Metabolic symbiosis between oxygenated and hypoxic tumour cells: An agent-based modelling study
Source: PLoS Comput Biol. 2024 Mar 15;20(3):e1011944. doi: 10.1371/journal.pcbi.1011944 (PMC10971686; doi:10.1371/journal.pcbi.1011944)
Supplement: S5 Fig — (A). TCGA breast cancer samples with high mRNA expression of network genes relative to normal samples. Genes are ordered from high to low of percentage of high mRNA samples. (B). TCGA breast cancer samples with low mRNA expression of network genes relative to normal samples. Genes are ordered from high to low of percentage of low mRNA samples. The horizontal axis shows samples with high (red) or low (blue) expressions of respective genes. We considered the gene as over-expressed when the standard deviation was above +3 with respect to normal samples and under-expressed as when it was below -3. (DOCX) [file pcbi.1011944.s009.docx]

# **S5 Fig**

**A**
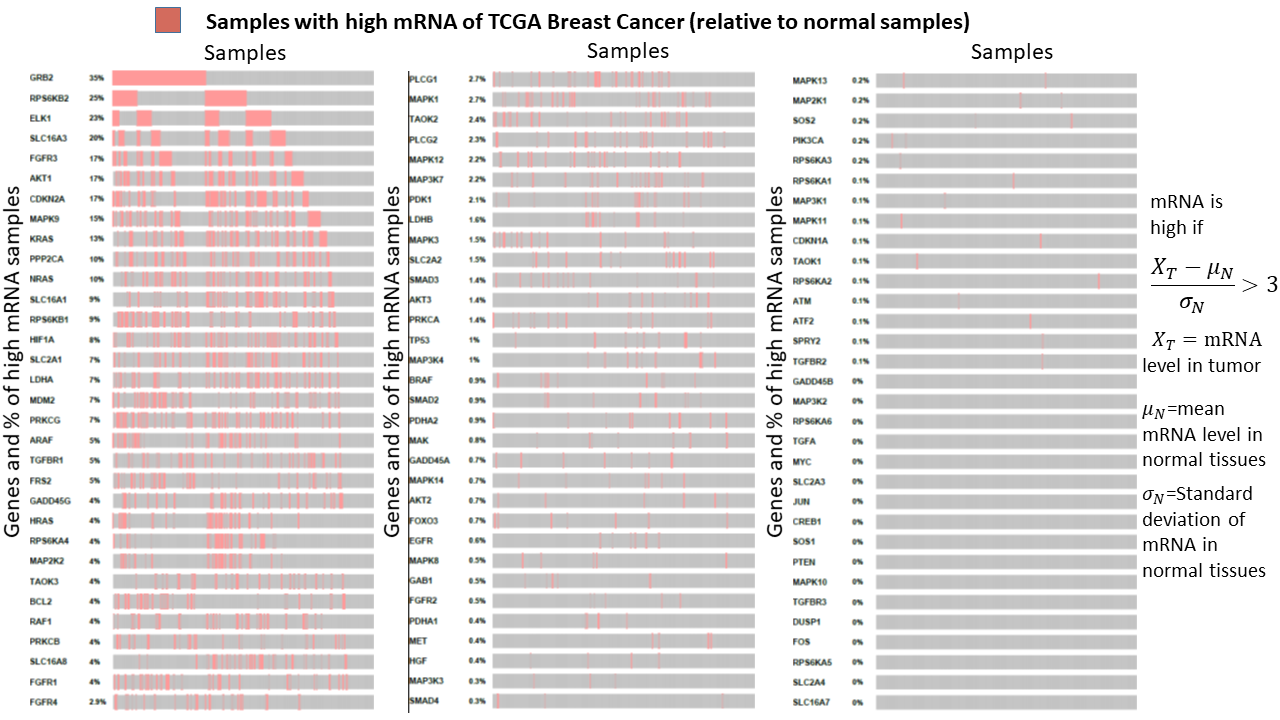


**B**
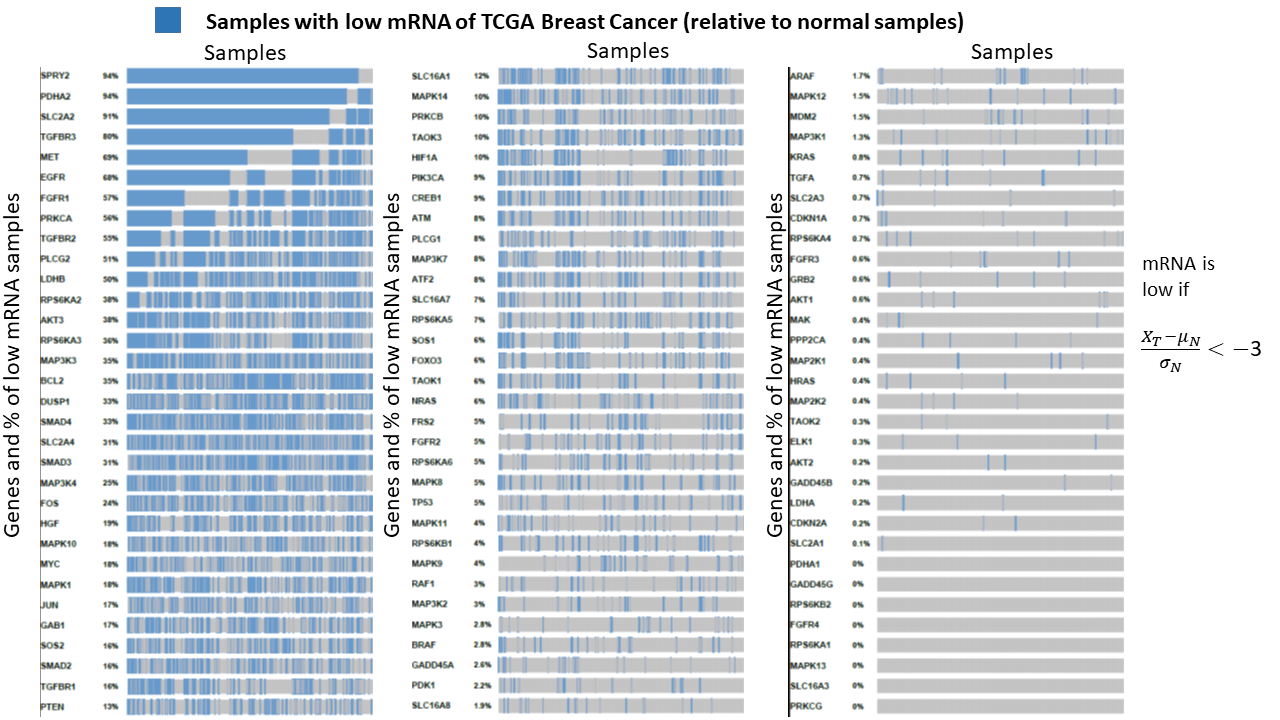


**S5 Fig:** **(A)**. TCGA breast cancer samples with high mRNA expression of network genes relative to normal samples. Genes are ordered from high to low of percentage of high mRNA samples. **(B)**. TCGA breast cancer samples with low mRNA expression of network genes relative to normal samples. Genes are ordered from high to low of percentage of low mRNA samples. The horizontal axis shows samples with high (red) or low (blue) expressions of respective genes. We considered the gene as over-expressed when the standard deviation was above +3 with respect to normal samples and under-expressed as when it was below -3.
