# Supplementary material for: Metabolic symbiosis between oxygenated and hypoxic tumour cells: An agent-based modelling study
Source: PLoS Comput Biol. 2024 Mar 15;20(3):e1011944. doi: 10.1371/journal.pcbi.1011944 (PMC10971686; doi:10.1371/journal.pcbi.1011944)
Supplement: S6 Fig — (A). Correlation coefficients between genes are shown for TCGA data. (B). Correlation coefficients between genes are shown for CCLE data. (C). Distribution of correlation coefficients and comparison of sigQC metrics calculated for genes using TCGA and CCLE datasets. The distribution of the metrics are fairly similar between two datasets. (DOCX) [file pcbi.1011944.s010.docx]

# **S6 Fig**

**A**

Correlation coefficient for TCGA breast cancer mRNA data

**B**

Correlation coefficient for CCLE breast cancer mRNA data

**C**


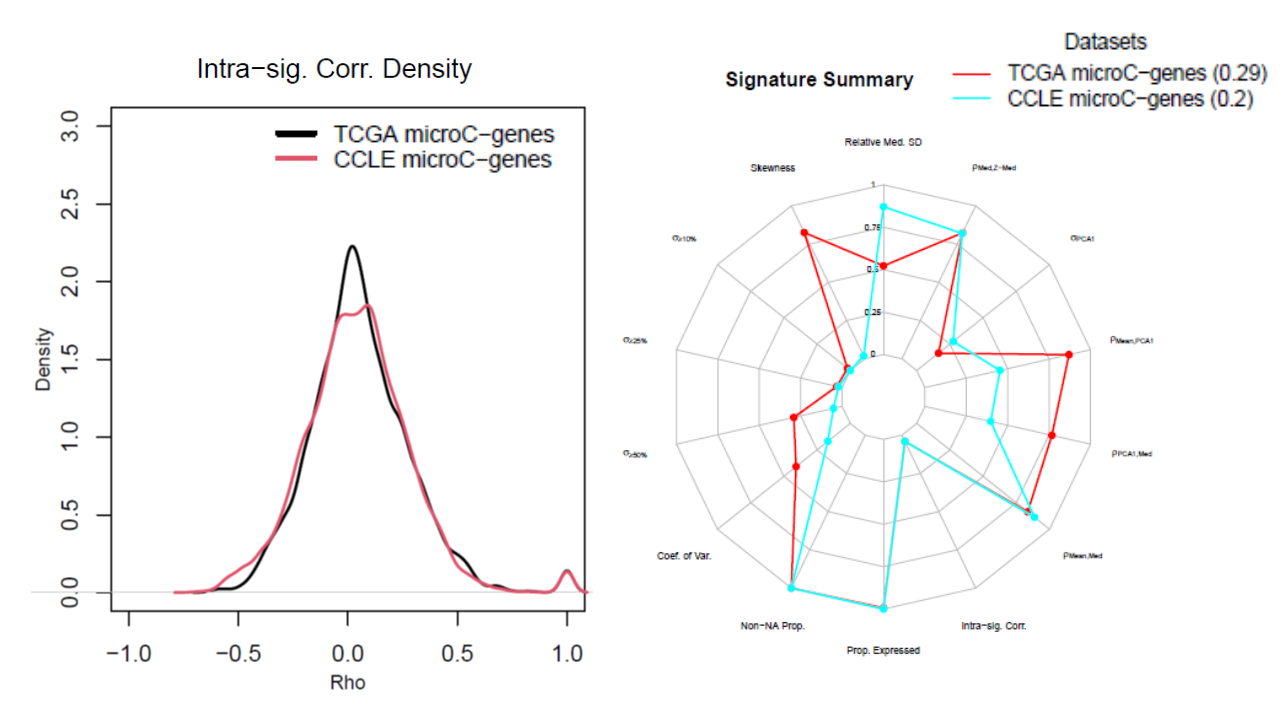


**S6 Fig. sigQC metrics were calculated for our network genes in TCGA and CCLE breast cancer RNA Seq. datasets:** **(A)**. Correlation coefficients between genes are shown for TCGA data. **(B)**. Correlation coefficients between genes are shown for CCLE data. **(C)**. Distribution of correlation coefficients and comparison of sigQC metrics calculated for genes using TCGA and CCLE datasets. The distribution of the metrics are fairly similar between two datasets.
