# Supplementary material for: Metabolic symbiosis between oxygenated and hypoxic tumour cells: An agent-based modelling study
Source: PLoS Comput Biol. 2024 Mar 15;20(3):e1011944. doi: 10.1371/journal.pcbi.1011944 (PMC10971686; doi:10.1371/journal.pcbi.1011944)
Supplement: S7 Fig — (A). Temporal variation of symbiosis index at each gene enrichment status. (B). Temporal variation of symbiosis index at each gene knockout status. (C). Whether symbiosis-induced growth is greater than non-symbiotic tumour growth with each gene enrichment is shown. (D). Whether symbiosis-induced growth is greater than non-symbiotic tumour growth with each gene knockout status is shown. The results show that clusters of gene alterations can be identified, which enhance symbiosis while some other gene alterations reduce symbiosis (A, B). Colors indicate symbiosis index (A, B) and p values (C, D). p values from 0 to 0.05 are shown in red to white color scale and p values ≥ 0.05 are shown in grey color. (E). Genes are clustered based on p-value is less than 0.05 (red) or not (white) of the results shown in Fig 4C (top-left), Fig 4D (top-right), S7C Fig (bottom-left), and S7D Fig (bottom-right). (DOCX) [file pcbi.1011944.s011.docx]

# **S7 Fig**

**A**
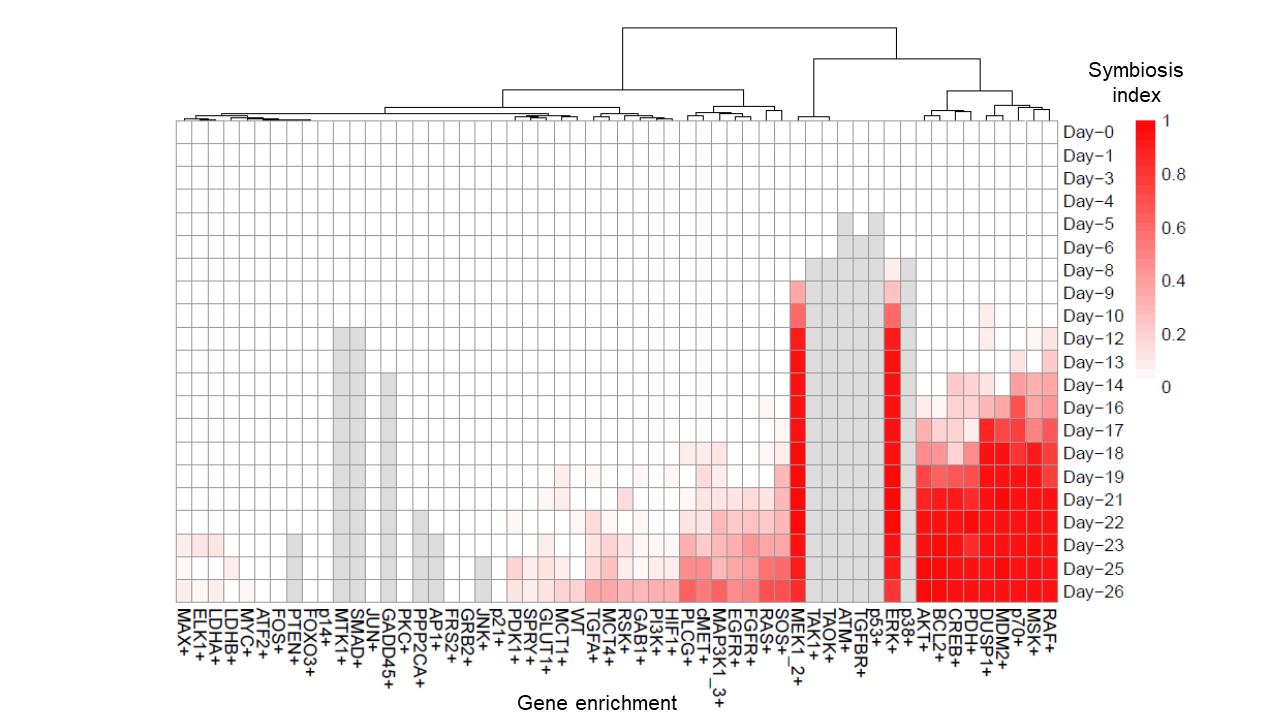


**B**

**
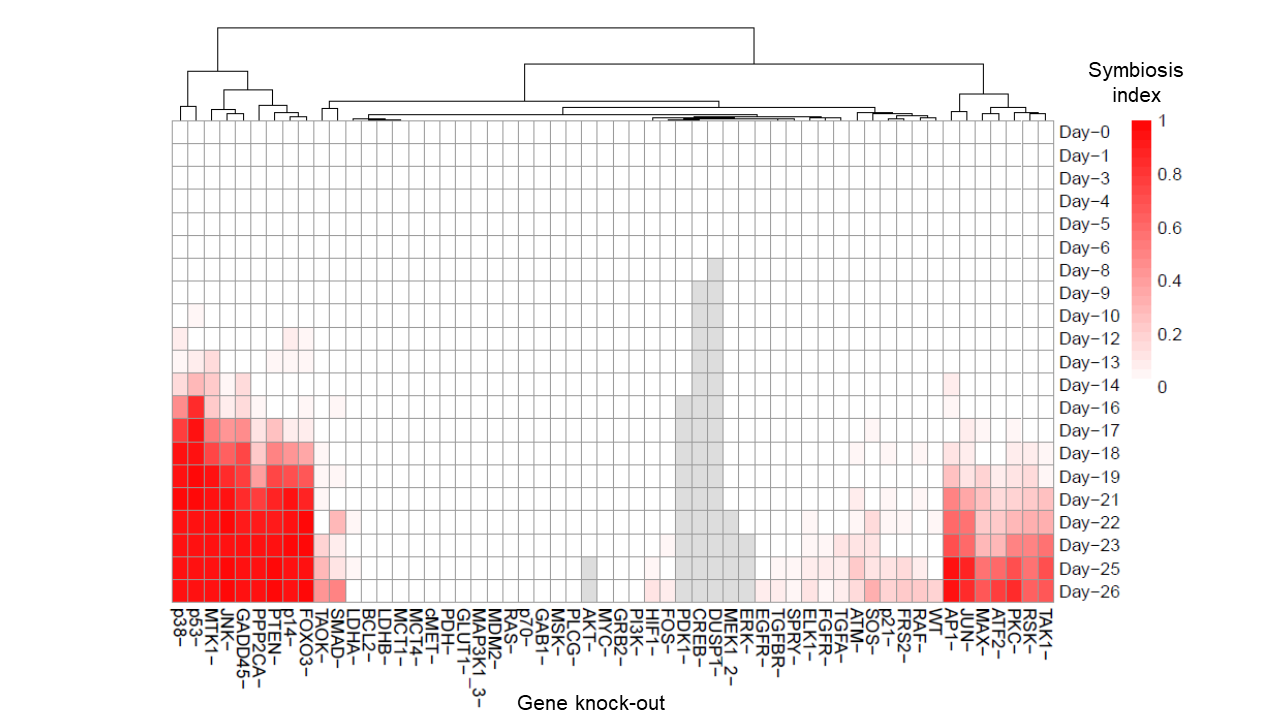
**

**C**

**
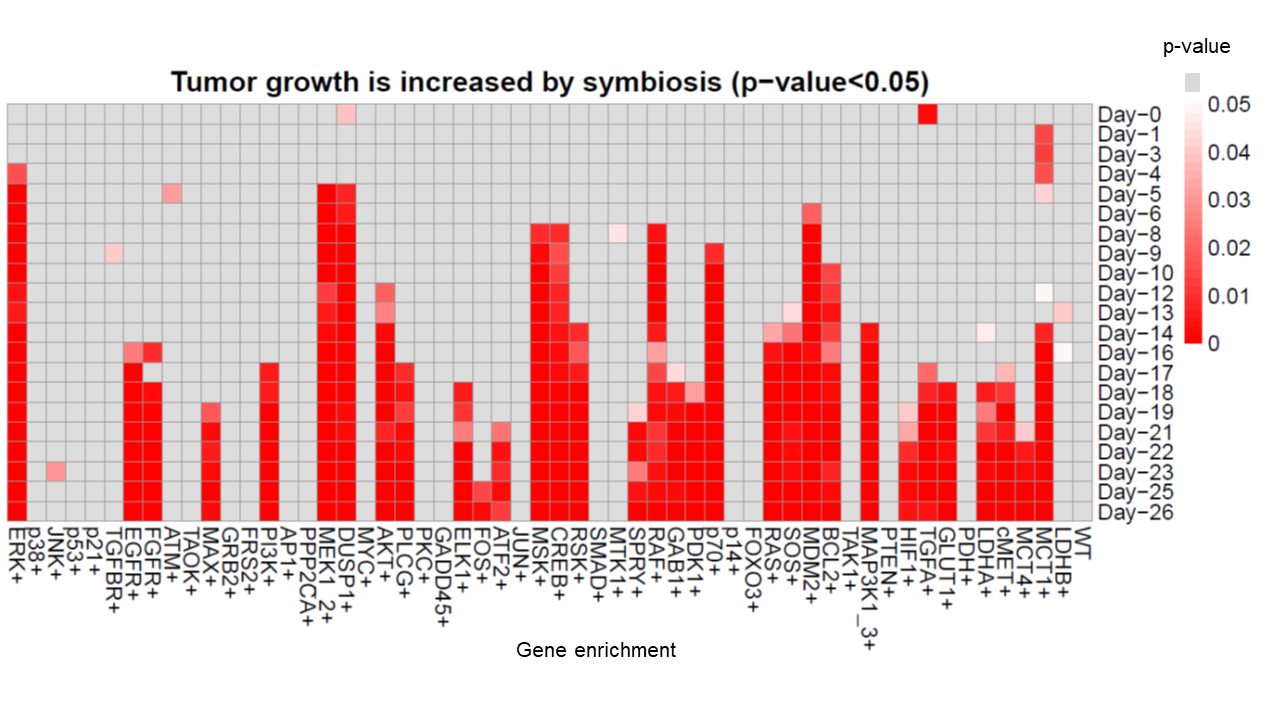
**

**D
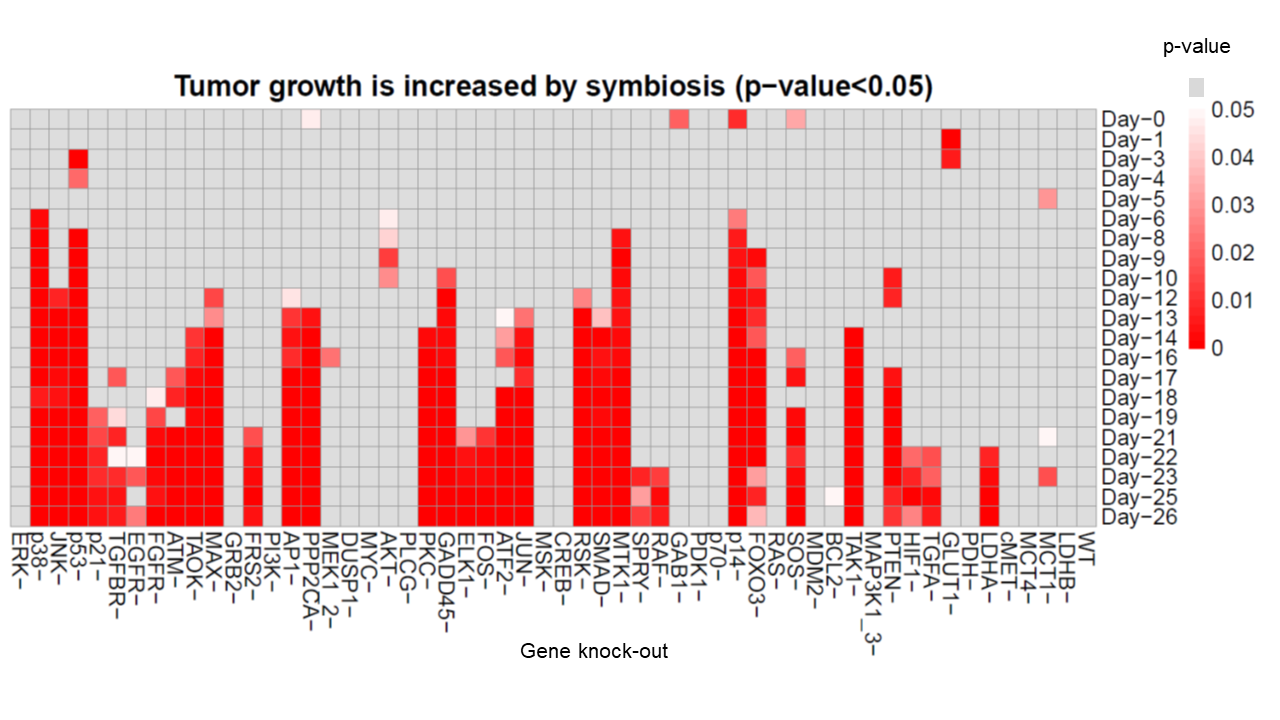
**

**E**

**
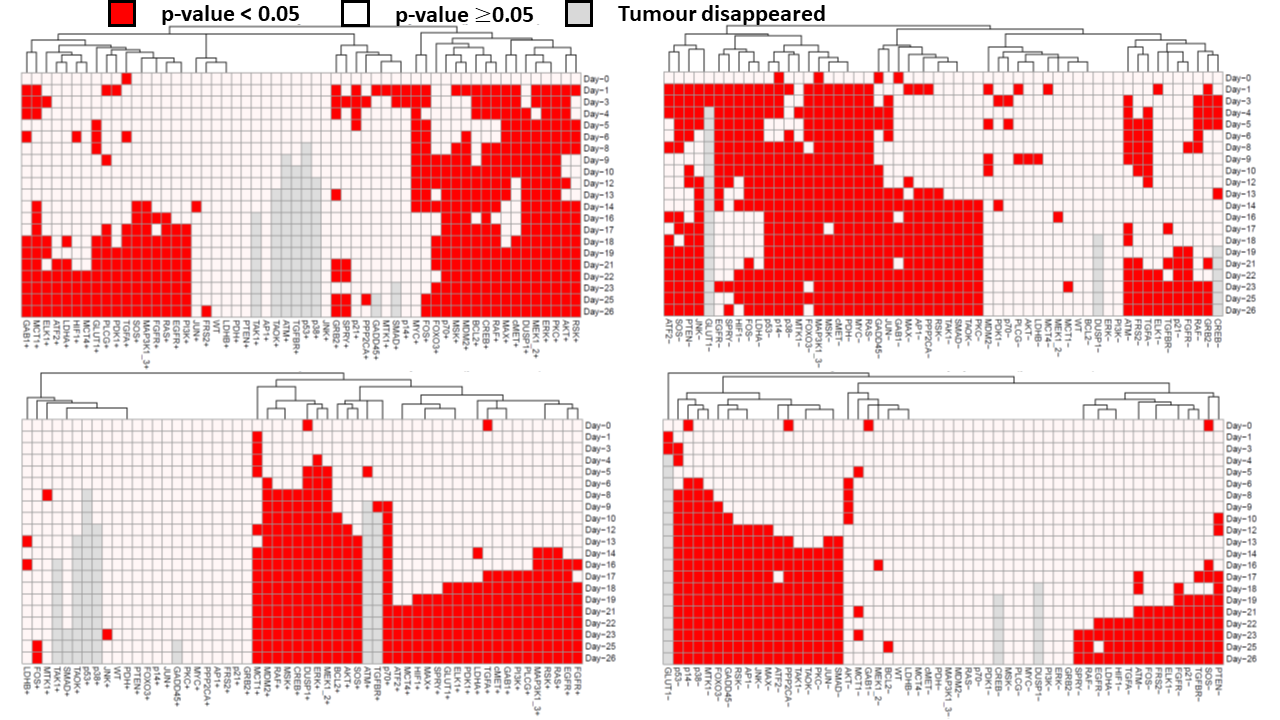
**

**S7 Fig. Metabolic symbiotic simulations with network gene alterations (Enriched (+) and Knockout (-) status) and wild type (WT) status:** **(A)**. Temporal variation of symbiosis index at each gene enrichment status. **(B)**. Temporal variation of symbiosis index at each gene knockout status. **(C)**. Whether symbiosis-induced growth is greater than non-symbiotic tumour growth with each gene enrichment is shown. **(D)**. Whether symbiosis-induced growth is greater than non-symbiotic tumour growth with each gene knockout status is shown. The results show that clusters of gene alterations can be identified, which enhance symbiosis while some other gene alterations reduce symbiosis (A, B). Colors indicate symbiosis index (A, B) and p values (C, D). p values from 0 to 0.05 are shown in red to white color scale and p values $\geq$ 0.05 are shown in grey color. **(E)**. Genes are clustered based on p-value is less than 0.05 (red) or not (white) of the results shown in Fig 4C (top-left), Fig 4D (top-right), S7C Fig (bottom-left), and S7D Fig (bottom-right).
