# Supplementary material for: Metabolic symbiosis between oxygenated and hypoxic tumour cells: An agent-based modelling study
Source: PLoS Comput Biol. 2024 Mar 15;20(3):e1011944. doi: 10.1371/journal.pcbi.1011944 (PMC10971686; doi:10.1371/journal.pcbi.1011944)
Supplement: S8 Fig — Here, the Length is the cross section through the center of the tumour. The heat maps show the variation of the percentage change of oxygen and glucose due to metabolic symbiosis under p53wt and p53- status, and at different initial tumour sizes. (A). The symbiosis would increase the glucose level in the medium. (B). The symbiosis would decrease the oxygen level in the medium. (DOCX) [file pcbi.1011944.s012.docx]

# **S8 Fig**

**A**

**
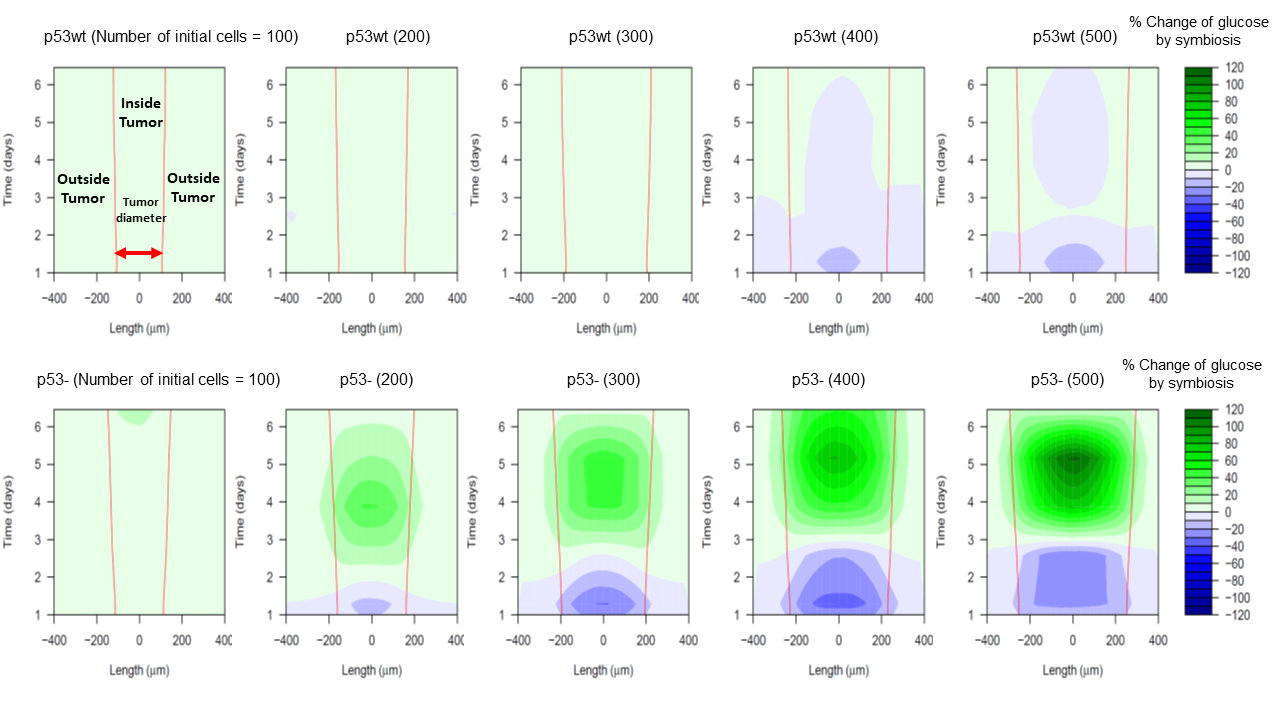
**

**B**

**
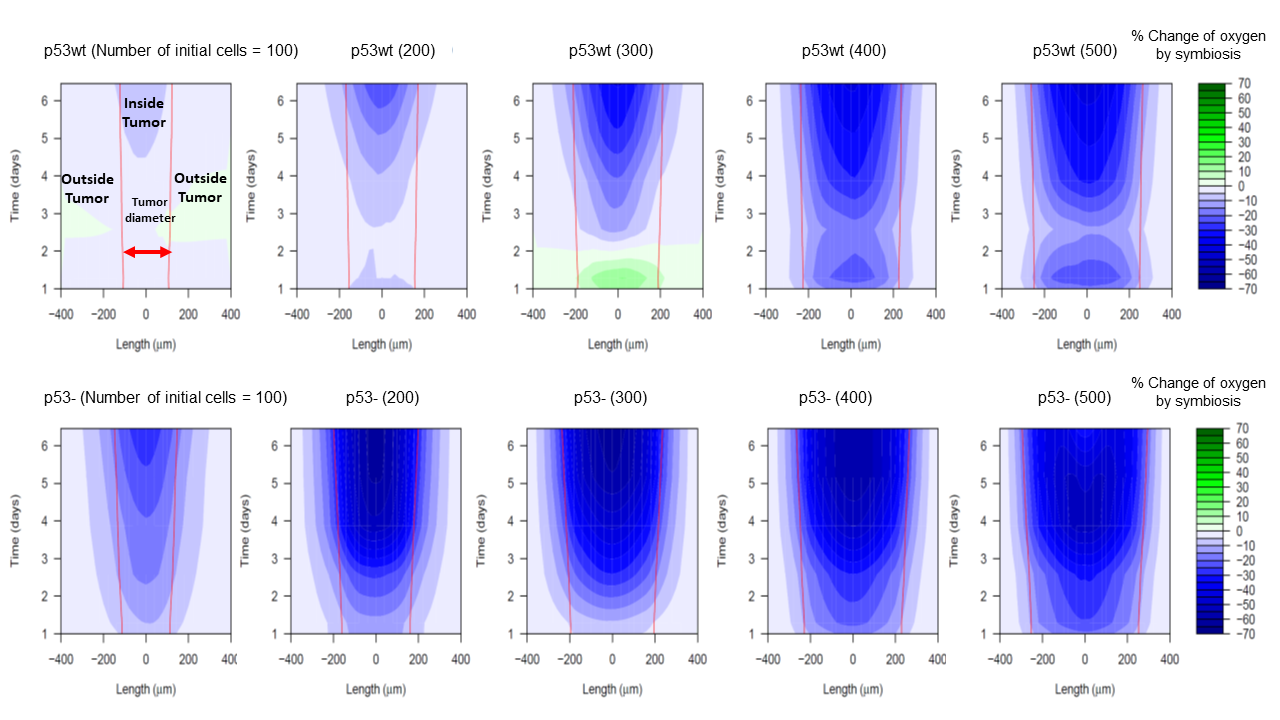
**

**S8 Fig. Symbiosis-induced changes of glucose and oxygen of the microenvironment over time:** Here, the Length is the cross section through the center of the tumour. The heat maps show the variation of the percentage change of oxygen and glucose due to metabolic symbiosis under p53wt and p53- status, and at different initial tumour sizes. **(A)**. The symbiosis would increase the glucose level in the medium. **(B)**. The symbiosis would decrease the oxygen level in the medium.
