# Supplementary material for: Metabolic symbiosis between oxygenated and hypoxic tumour cells: An agent-based modelling study
Source: PLoS Comput Biol. 2024 Mar 15;20(3):e1011944. doi: 10.1371/journal.pcbi.1011944 (PMC10971686; doi:10.1371/journal.pcbi.1011944)
Supplement: S9 Fig — (A). p53wt cells with 6% oxygen level. (B). p53wt cells with 3% oxygen level. (C). p53- cells with 6% oxygen level. (D). p53- cells with 3% oxygen level. The results show that both glucose and lactate metabolism can fuel tumour growth when tumour is well-oxygenated. (DOCX) [file pcbi.1011944.s013.docx]

# **S9 Fig**

**A**


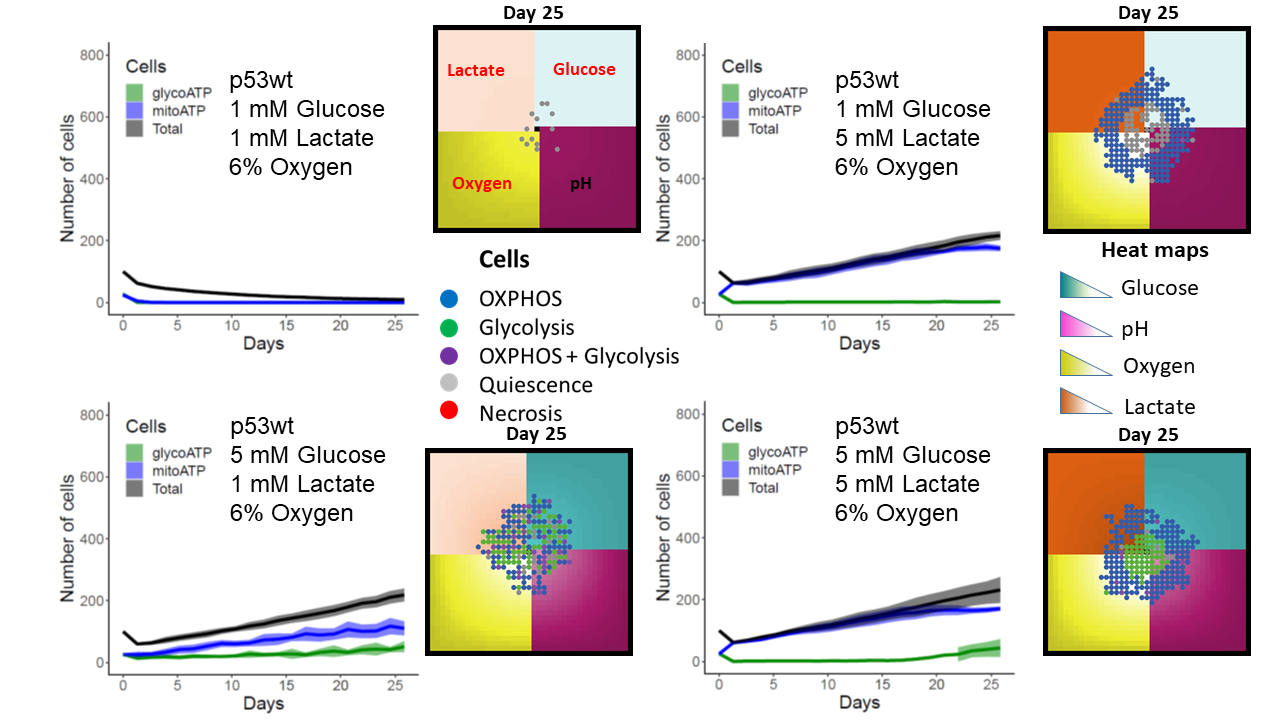


**B**


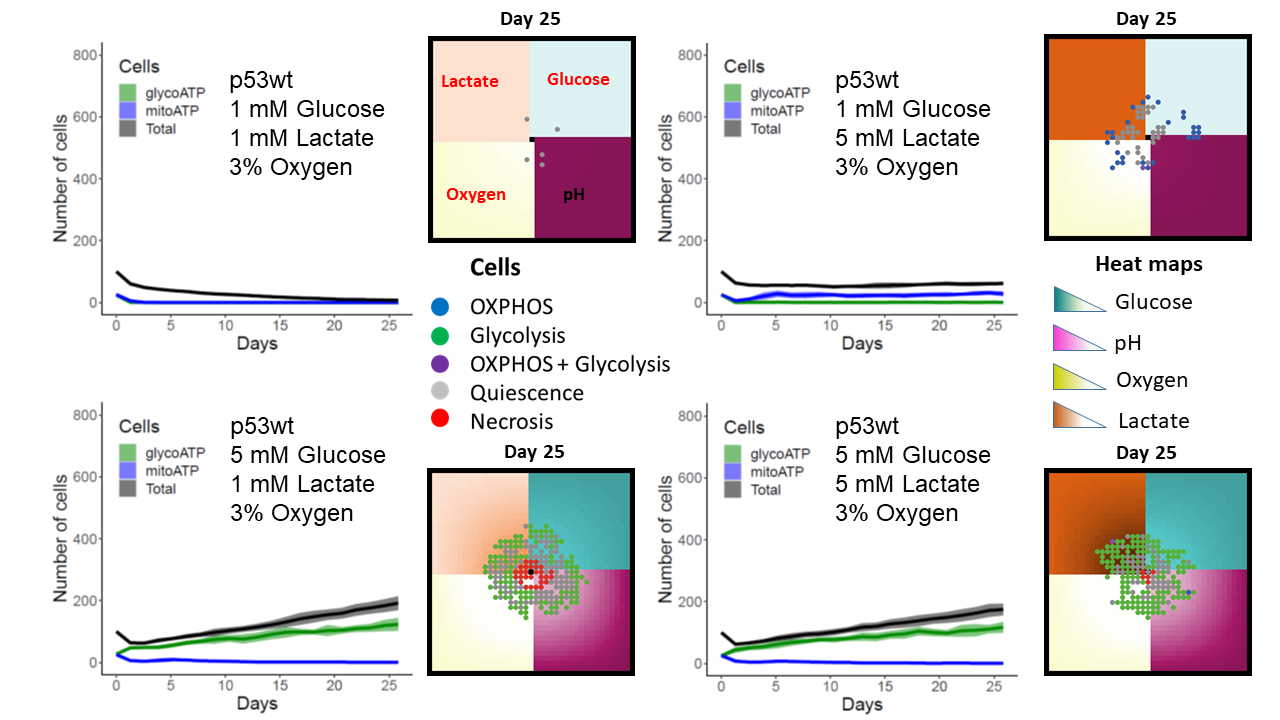


**C**


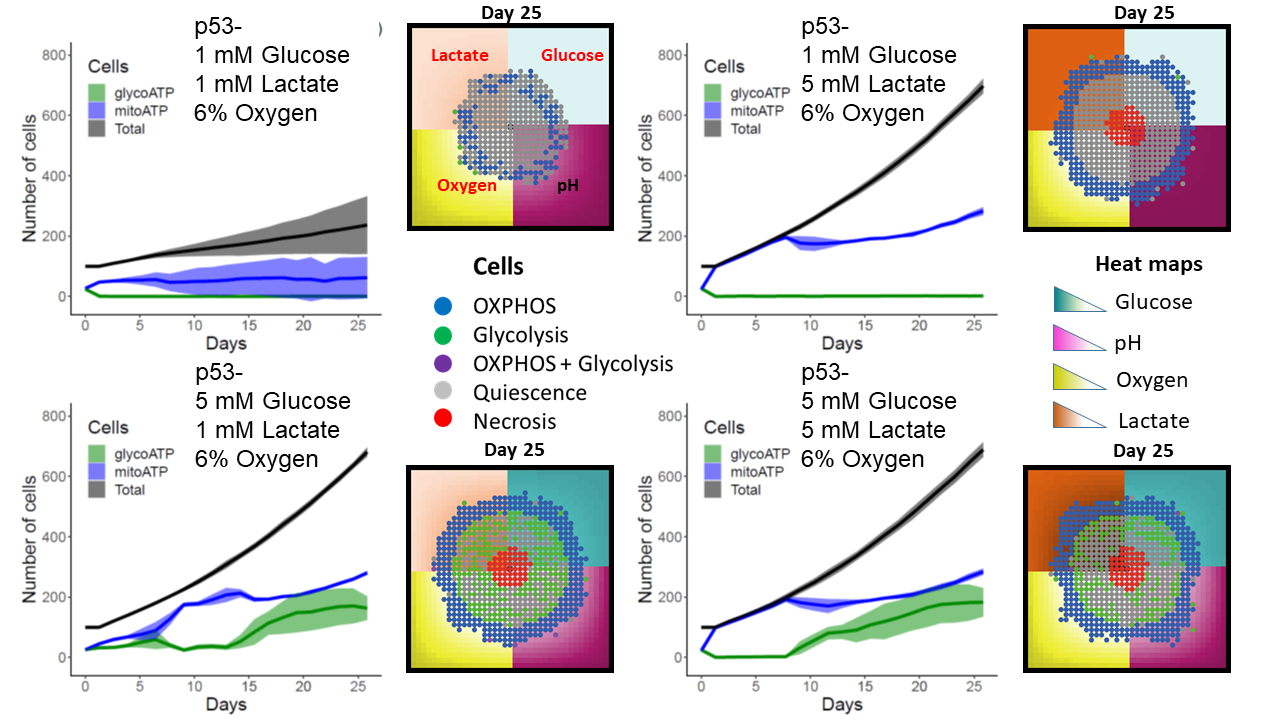


**D**


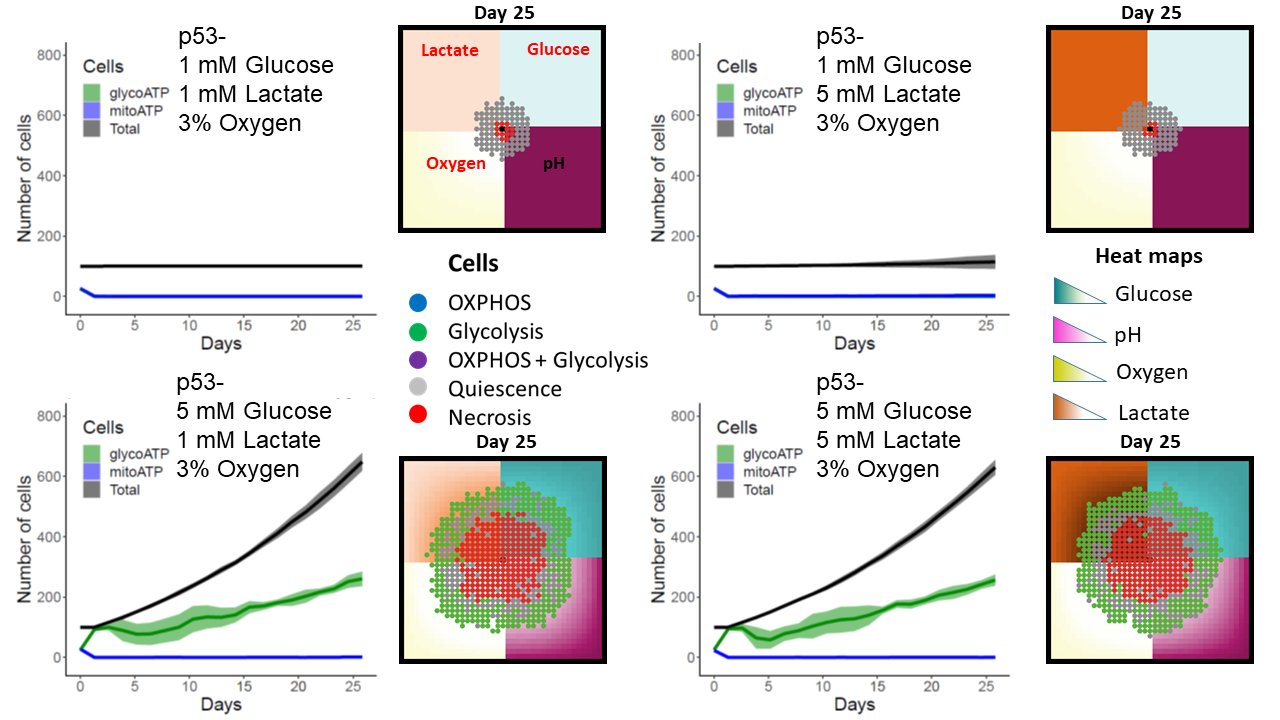


**S9 Fig. Tumour growth under different environmental conditions (different levels of glucose, lactate and oxygen) are shown for p53wt and p53- cells:** **(A)**. p53wt cells with 6% oxygen level. **(B)**. p53wt cells with 3% oxygen level. **(C)**. p53- cells with 6% oxygen level. **(D)**. p53- cells with 3% oxygen level. The results show that both glucose and lactate metabolism can fuel tumour growth when tumour is well-oxygenated.
