# Supplementary material for: Metabolic symbiosis between oxygenated and hypoxic tumour cells: An agent-based modelling study
Source: PLoS Comput Biol. 2024 Mar 15;20(3):e1011944. doi: 10.1371/journal.pcbi.1011944 (PMC10971686; doi:10.1371/journal.pcbi.1011944)
Supplement: S10 Fig — As the oxygen level is increased, more tumour cells switch to OXPHOS because they have both glucose and lactate as the energy source for mitochondrial ATP production. The oxygen level at the boundary of the simulation domain (square box) was maintained at 3%, 6%, and 9% O2 while the glucose level was at 1 mM (A), 5 mM (B) and 10 mM (C). Note that oxygen and glucose levels in the tumour were much lower than these boundary values. (DOCX) [file pcbi.1011944.s014.docx]

# **S10 Fig**

**A**

**
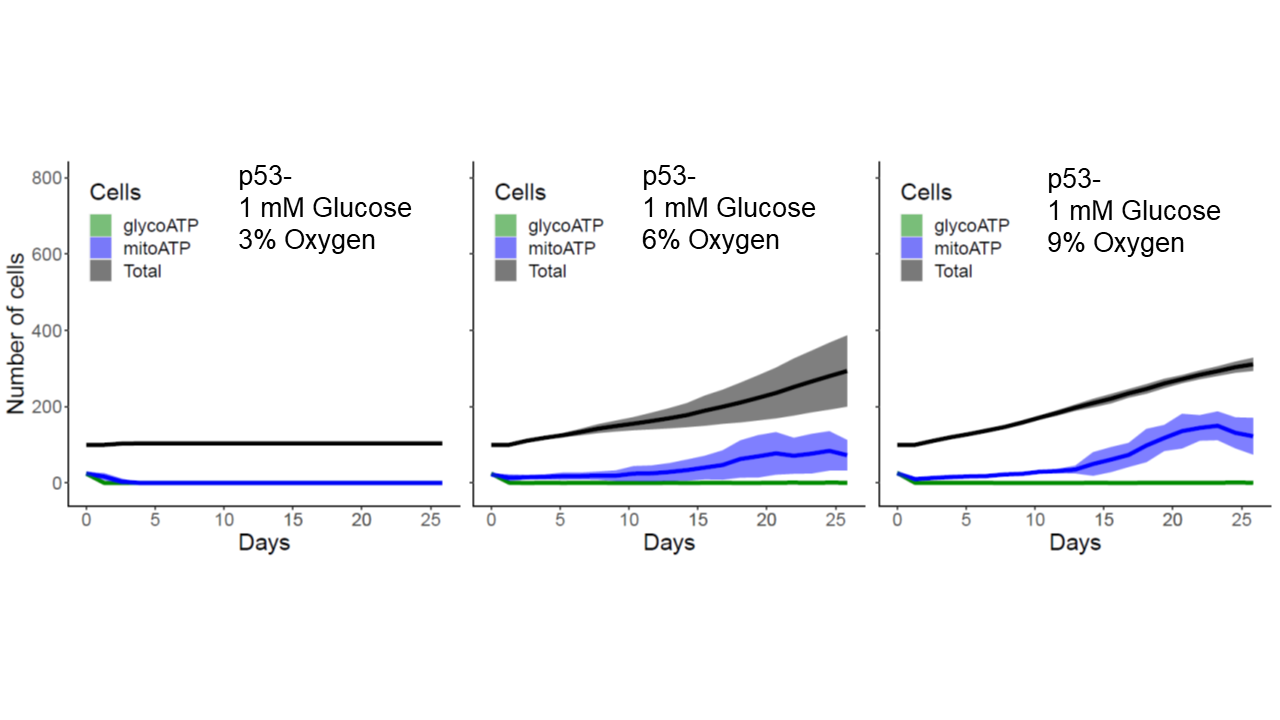
**

**B**


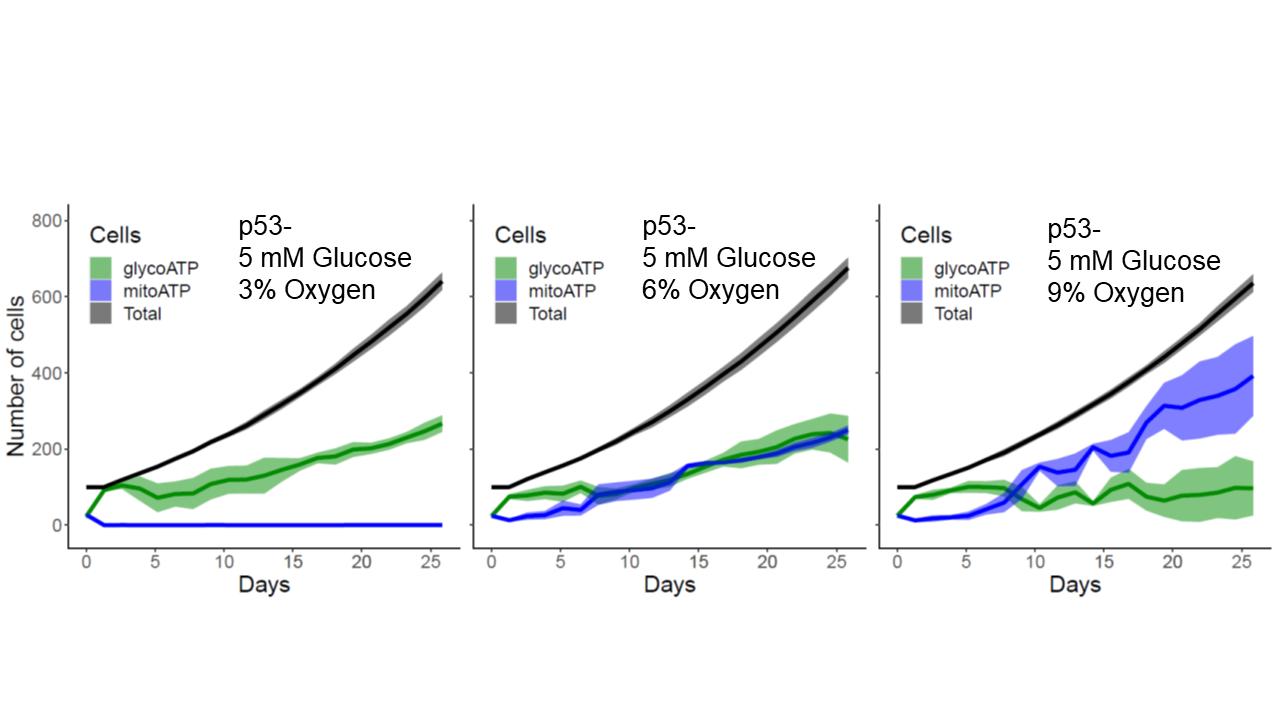


**C**


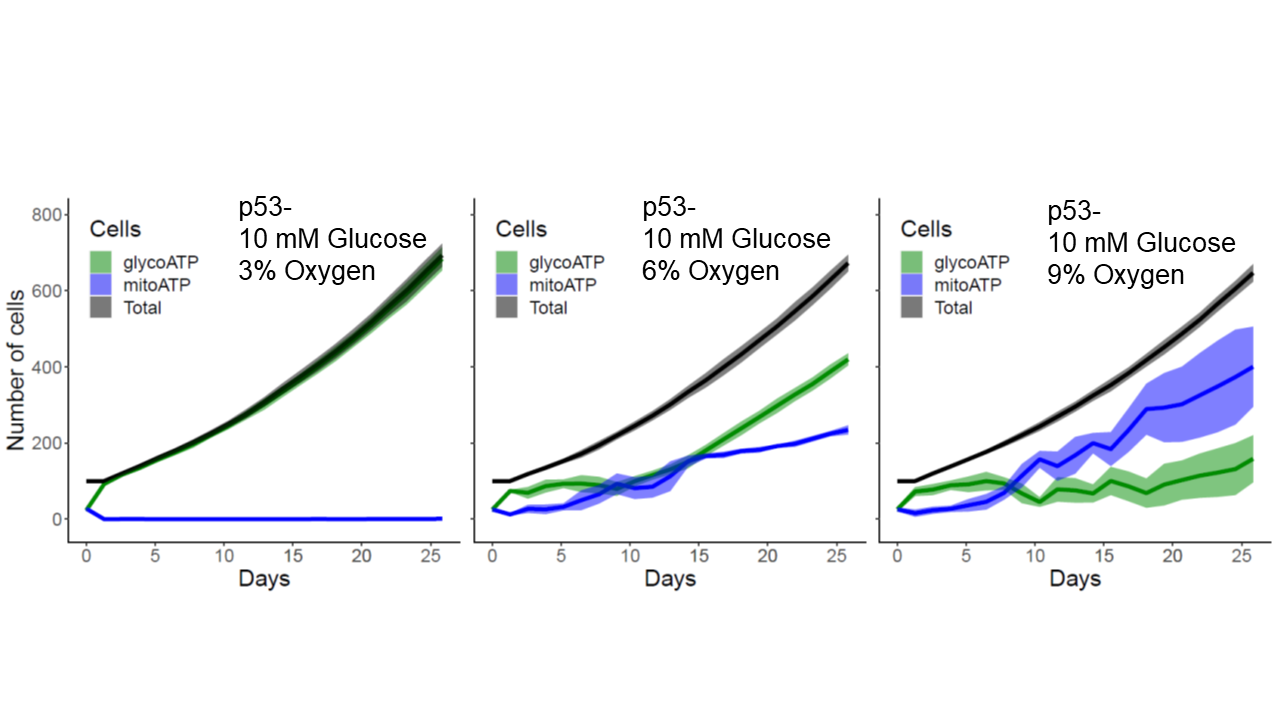


**S10 Fig. Tumour growth curves are shown for different combinations of oxygen and glucose:** As the oxygen level is increased, more tumour cells switch to OXPHOS because they have both glucose and lactate as the energy source for mitochondrial ATP production. The oxygen level at the boundary of the simulation domain (square box) was maintained at 3%, 6%, and 9% O2 while the glucose level was at 1 mM (A), 5 mM (B) and 10 mM (C). Note that oxygen and glucose levels in the tumour were much lower than these boundary values.
