# Supplementary material for: Metabolic symbiosis between oxygenated and hypoxic tumour cells: An agent-based modelling study
Source: PLoS Comput Biol. 2024 Mar 15;20(3):e1011944. doi: 10.1371/journal.pcbi.1011944 (PMC10971686; doi:10.1371/journal.pcbi.1011944)
Supplement: S11 Fig — The half-saturation coefficient of oxygen was kept at 0.045% oxygen (A), 0.45% oxygen (B) and 4.5% oxygen (C) while the half saturation coefficient of glucose was varied to 0.004 mM, 0.04 mM and 0.4 mM. The pathways seem more sensitive to the variation of the half-saturation coefficient of oxygen. (DOCX) [file pcbi.1011944.s015.docx]

# **S11 Fig**

**A**


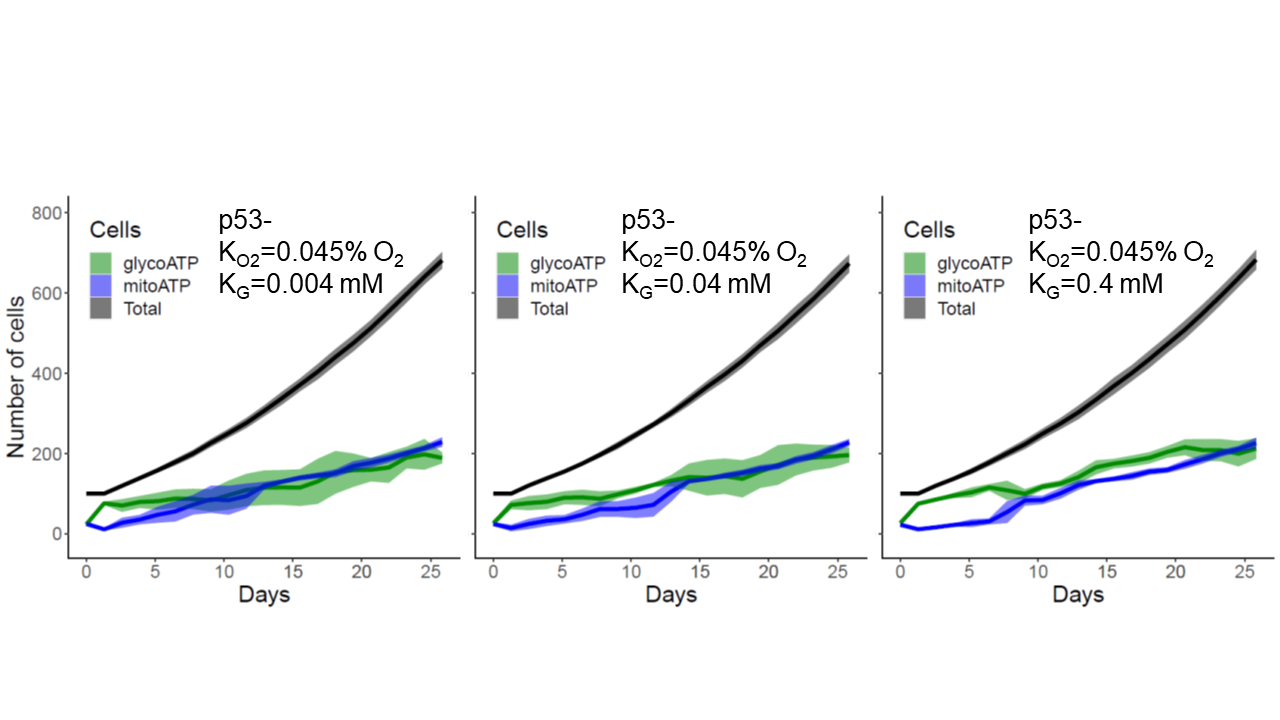


**B**


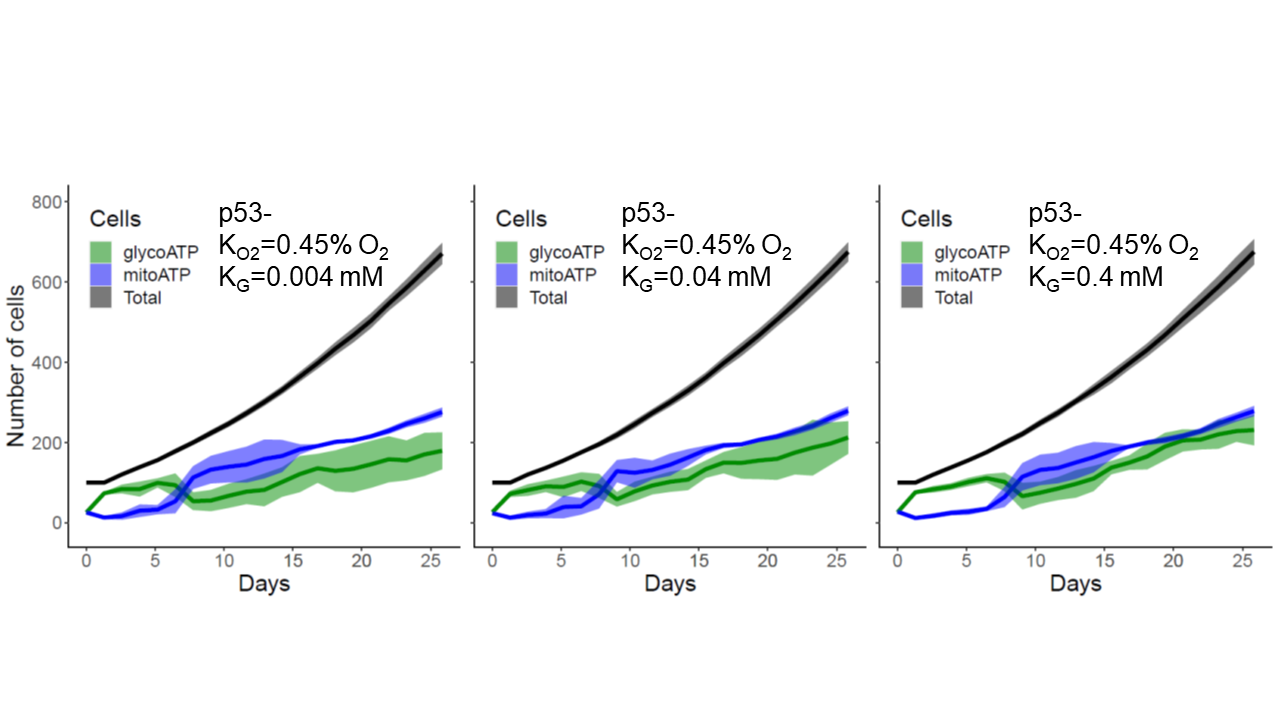


**C**


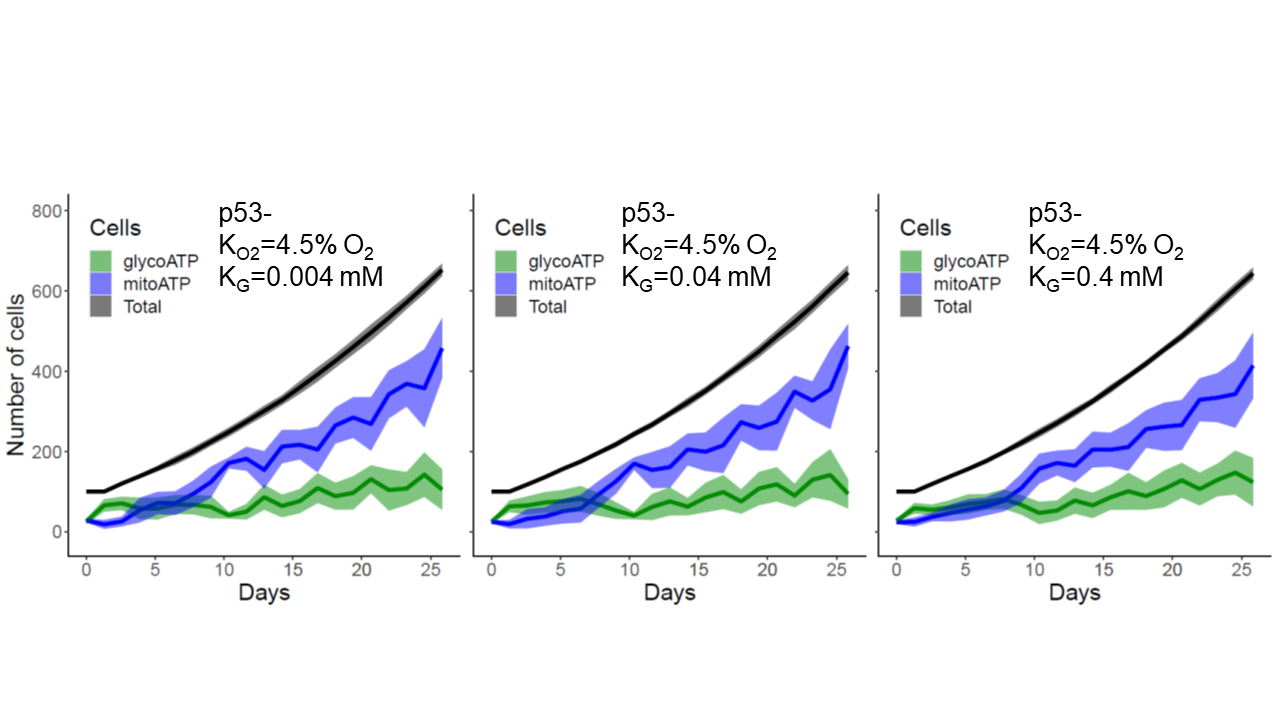


**S11 Fig. The effect of half-saturation coefficients of oxygen and glucose on the metabolic pathways is shown:** The half-saturation coefficient of oxygen was kept at 0.045% oxygen (A), 0.45% oxygen (B) and 4.5% oxygen (C) while the half saturation coefficient of glucose was varied to 0.004 mM, 0.04 mM and 0.4 mM. The pathways seem more sensitive to the variation of the half-saturation coefficient of oxygen.
