# Supplementary material for: Metabolic symbiosis between oxygenated and hypoxic tumour cells: An agent-based modelling study
Source: PLoS Comput Biol. 2024 Mar 15;20(3):e1011944. doi: 10.1371/journal.pcbi.1011944 (PMC10971686; doi:10.1371/journal.pcbi.1011944)
Supplement: S12 Fig — [GLUT1i]/IC50 and [MCT1i]/IC50 were varied from 0 to 100 and 0 to 1000, respectively. (A, B, C). p53wt tumour cells. Note that the tumour is completely disappeared at [GLUT1]/IC50 = 10. (D, E, F). p53- tumour cells. Temporal variations of total cells (A, D), glycolytic cells (B, E), and OXPHOS cells (C, F) are shown. (DOCX) [file pcbi.1011944.s016.docx]

# **S12 Fig**

**A**

**
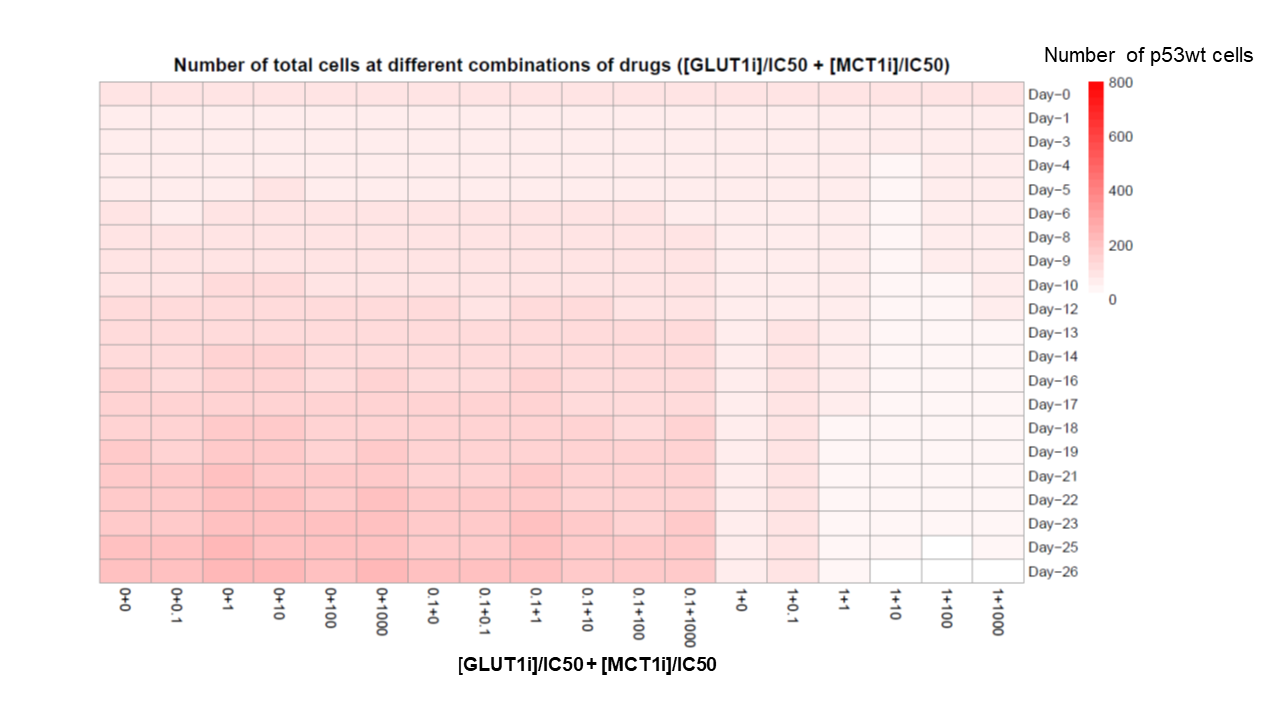
**

**B**


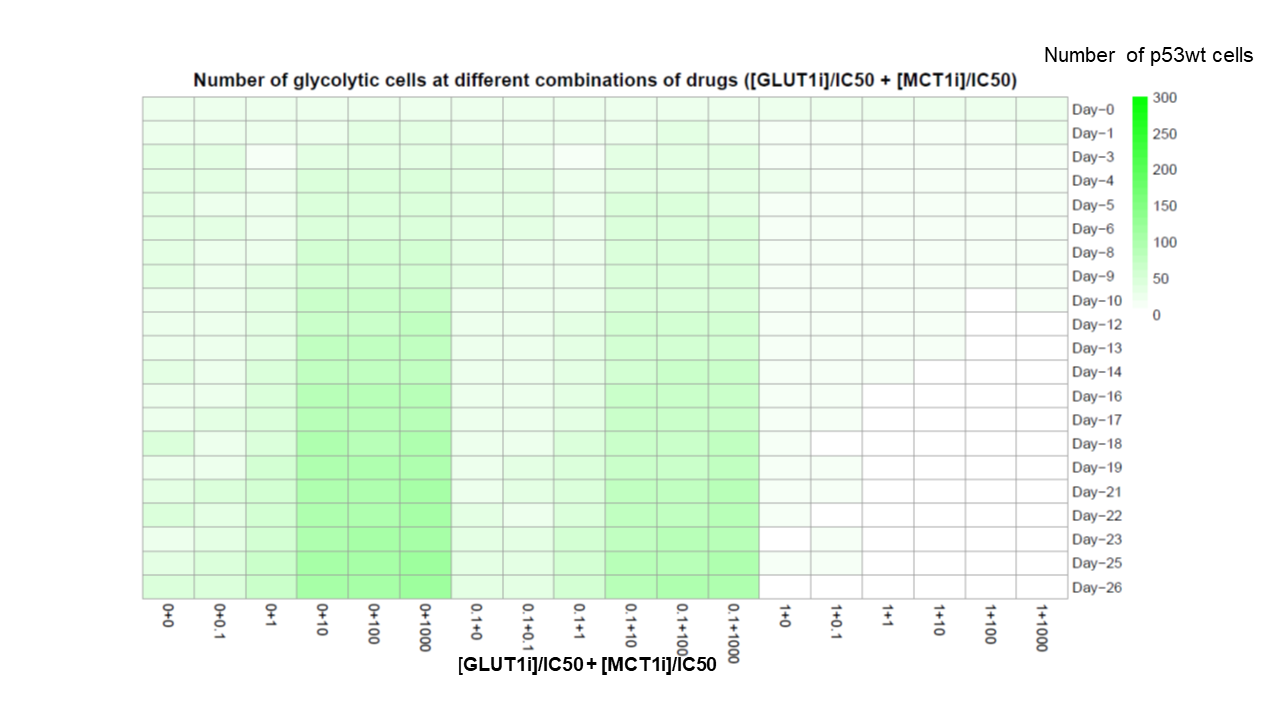


**C**

**
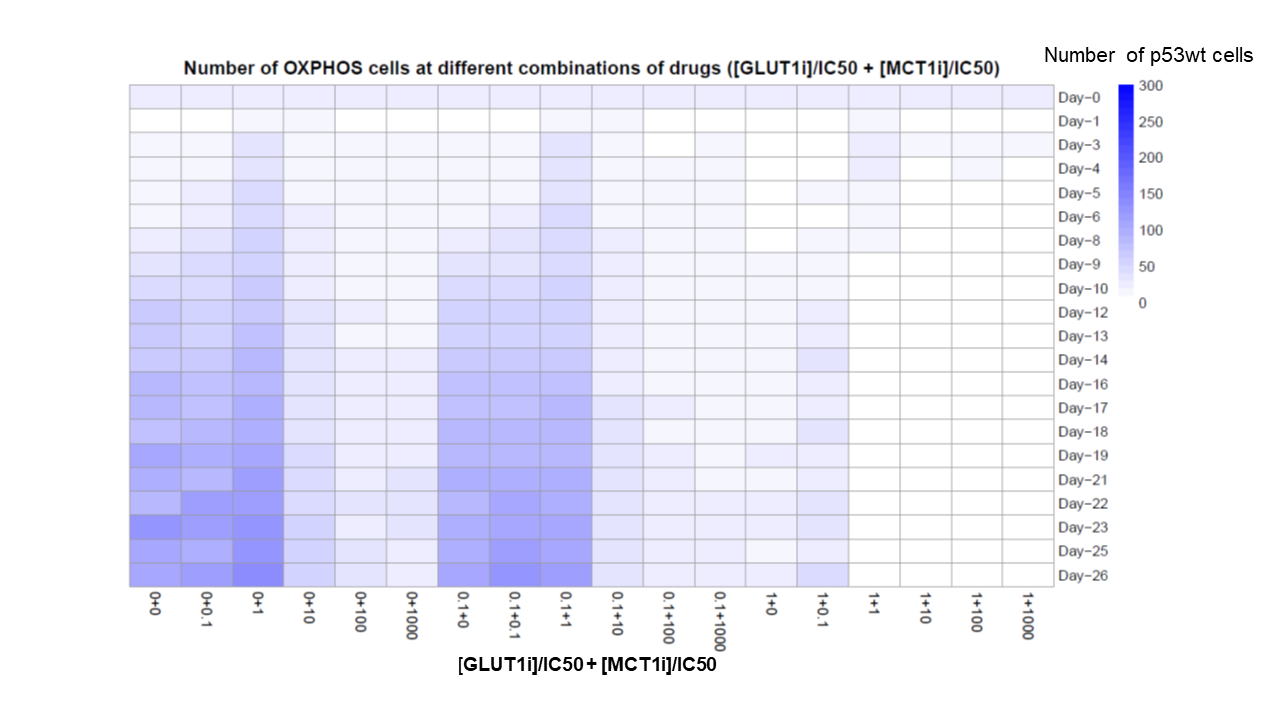
**

**D**

**
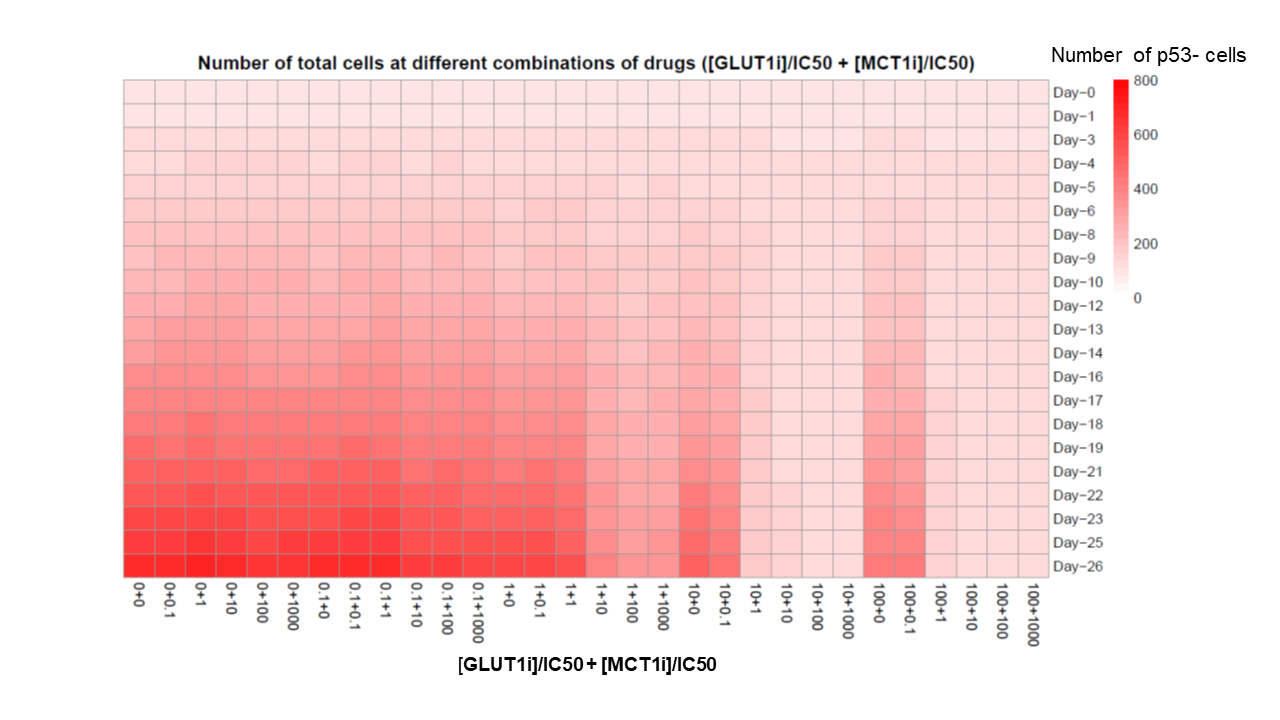
**

**E**


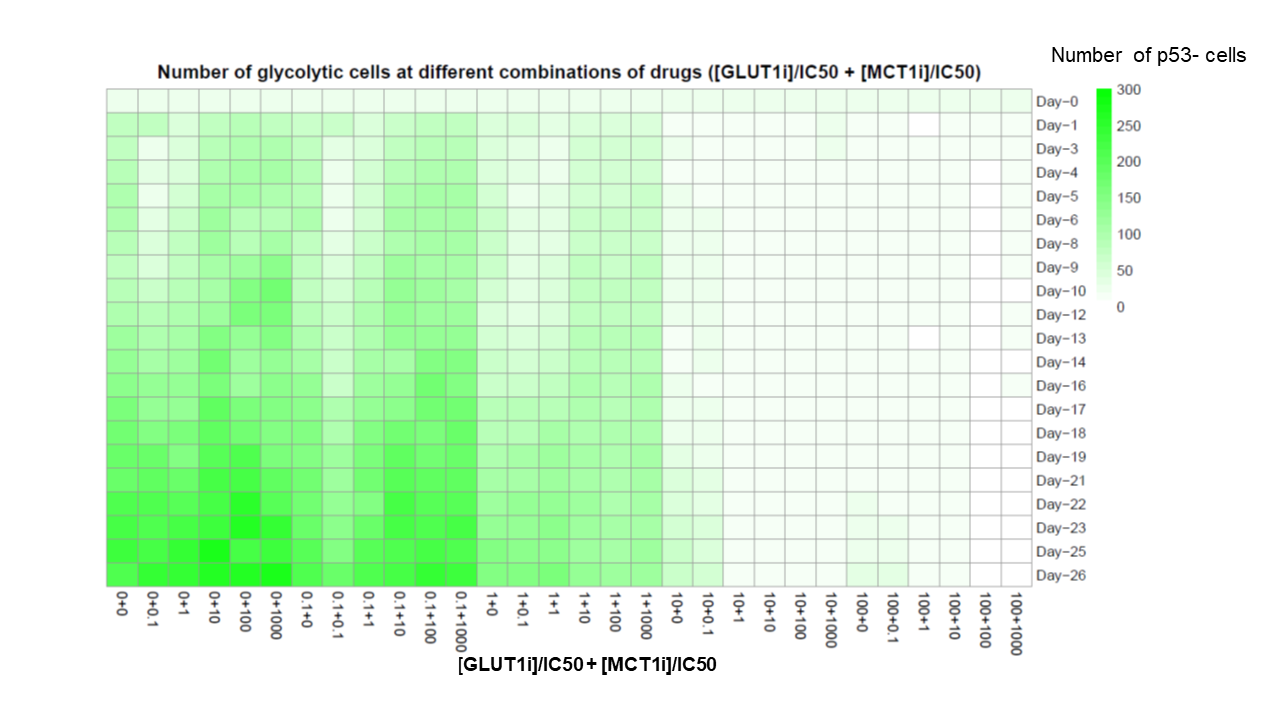


**F**


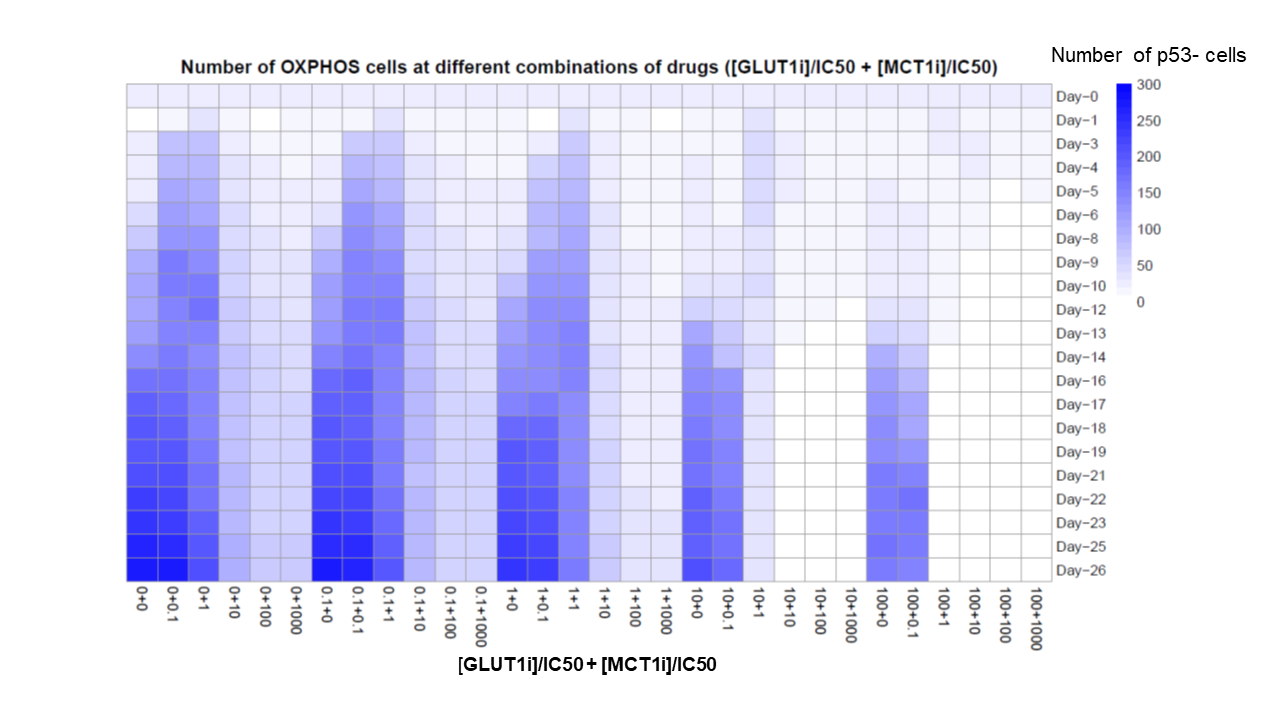


**S12 Fig. Tumour growth over time at different combinations of GLUT1 and MCT1 inhibitors:** [GLUT1i]/IC50 and [MCT1i]/IC50 were varied from 0 to 100 and 0 to 1000, respectively. **(A, B, C)**. p53wt tumour cells. Note that the tumour is completely disappeared at [GLUT1]/IC50 = 10. **(D, E, F)**. p53- tumour cells. Temporal variations of total cells (A, D), glycolytic cells (B, E), and OXPHOS cells (C, F) are shown.
