# Supplementary material for: Metabolic symbiosis between oxygenated and hypoxic tumour cells: An agent-based modelling study
Source: PLoS Comput Biol. 2024 Mar 15;20(3):e1011944. doi: 10.1371/journal.pcbi.1011944 (PMC10971686; doi:10.1371/journal.pcbi.1011944)
Supplement: S13 Fig — Bar charts depicting how MCT1 and GLUT1 inhibitors (denoted as MCT1i and GLUT1i) interfere with glycolysis, OXPHOS, and tumour growth. The MCT1i concentration, [MCT1i], was varied up to 1000 times of its half-maximal inhibitory concentration (IC50) value while GLUT1i concentration, [GLUT1i], was varied up to 100 times of its IC50 value. Inhibitor concentration was maintained at the boundary of the computational domain throughout the simulation time. (A). Number of glycolytic and mitochondrial ATP producing cells of p53wt tumour after 25 days of growth. (B). Number of glycolytic and mitochondrial ATP producing cells of p53- tumour after 25 days of growth. The initial tumour consisted of 100 cells, either with p53wt or p53- status. (DOCX) [file pcbi.1011944.s017.docx]

# **S13 Fig**

**A**


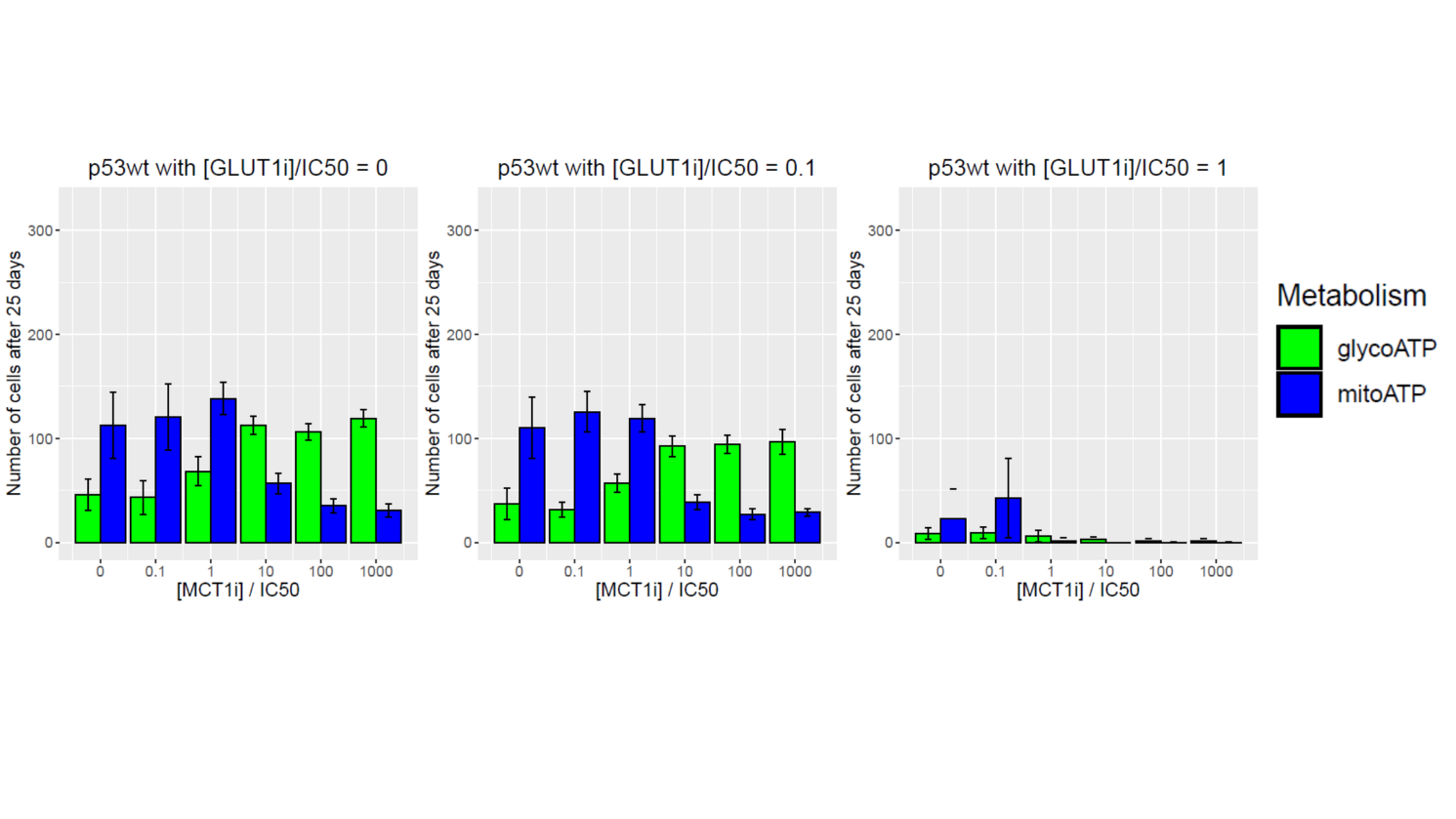


**B**


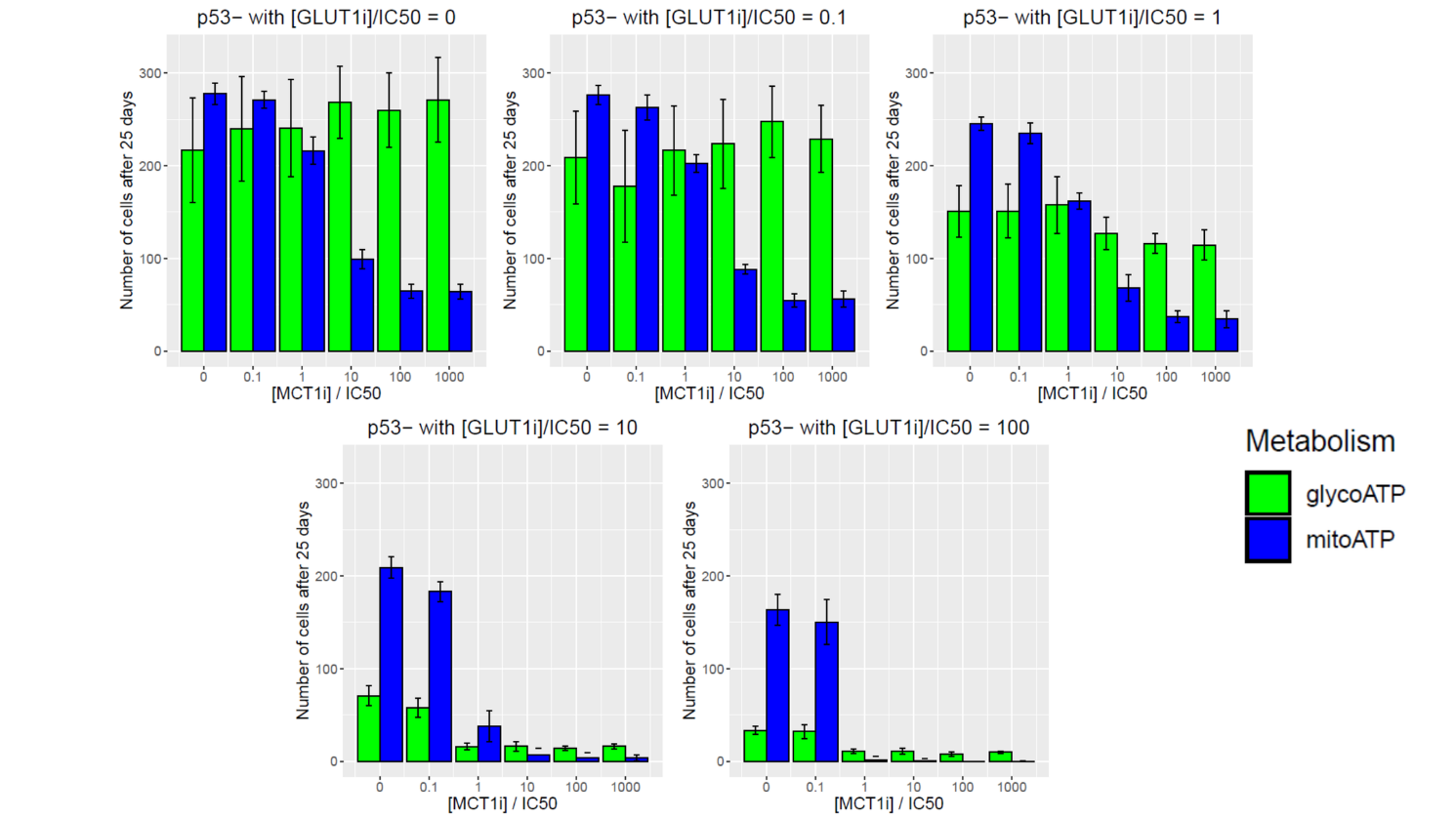


**S13 Fig. MCT1 and GLUT1 inhibition:** Bar charts depicting how MCT1 and GLUT1 inhibitors (denoted as MCT1i and GLUT1i) interfere with glycolysis, OXPHOS, and tumour growth. The MCT1i concentration, [MCT1i], was varied up to 1000 times of its half-maximal inhibitory concentration (IC50) value while GLUT1i concentration, [GLUT1i], was varied up to 100 times of its IC50 value. Inhibitor concentration was maintained at the boundary of the computational domain throughout the simulation time. **(A)**. Number of glycolytic and mitochondrial ATP producing cells of p53wt tumour after 25 days of growth. **(B)**. Number of glycolytic and mitochondrial ATP producing cells of p53- tumour after 25 days of growth. The initial tumour consisted of 100 cells, either with p53wt or p53- status.
