# Supplementary material for: Metabolic symbiosis between oxygenated and hypoxic tumour cells: An agent-based modelling study
Source: PLoS Comput Biol. 2024 Mar 15;20(3):e1011944. doi: 10.1371/journal.pcbi.1011944 (PMC10971686; doi:10.1371/journal.pcbi.1011944)
Supplement: S14 Fig — The simulations were initiated with a population of 100 p53- cells, and the inhibitor concentrations were set to [MCT1i]/IC50 = [GLUT1i]/IC50 = 10. (A). In the first scenario, MCT1 inhibitor was initially administered, followed by alternating treatments of MCT1 and GLUT1 inhibitors for a constant period. In the second scenario, both MCT1 and GLUT1 inhibitors were simultaneously applied for a constant period of time, followed by a therapy-free interval, and this cycle repeated each for 5 days, until the total simulation time reached 30 days. The period was set to 2, 5 and 7,5 days. (B). The control cases. The inhibitors were set to MCT1i]/IC50 = [GLUT1i]/IC50 = 5 and they were continuously applied (Left). No therapy was applied in this case (Right). (DOCX) [file pcbi.1011944.s018.docx]

# **S14 Fig**

**A**


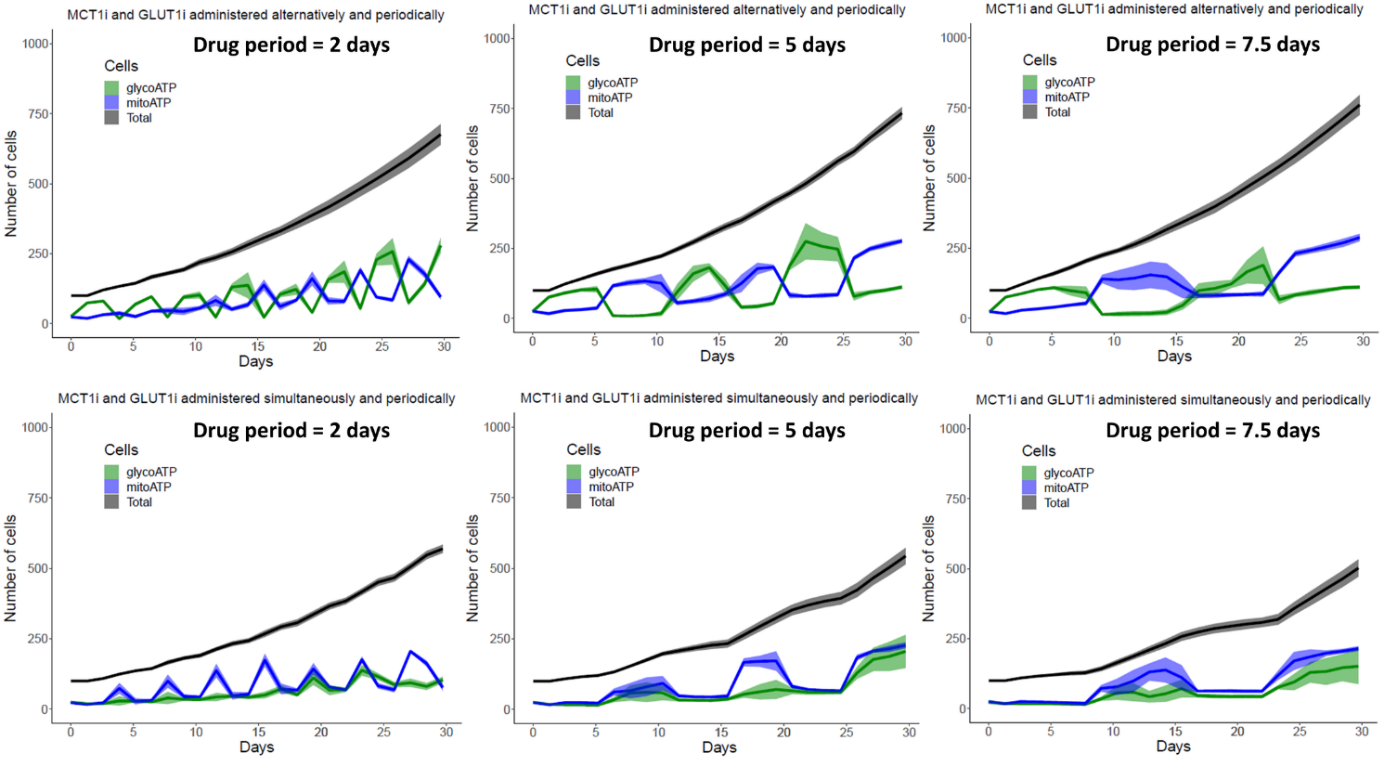


**B**


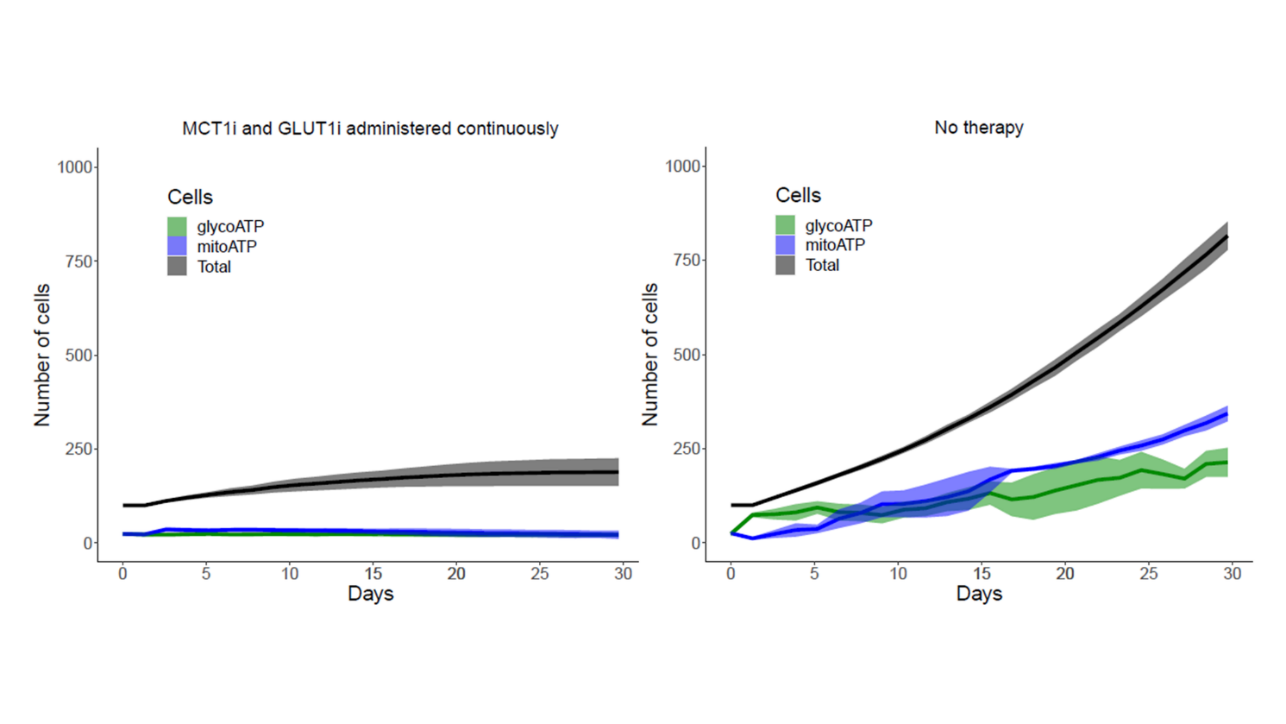


**S14 Fig. Dynamics of total, glycolytic (glycoATP), and OXPHOS (mitoATP) cell populations over time under different application strategies of MCT1 and GLUT1 inhibitors:** The simulations were initiated with a population of 100 p53- cells, and the inhibitor concentrations were set to [MCT1i]/IC50 = [GLUT1i]/IC50 = 10. **(A)**. In the first scenario, MCT1 inhibitor was initially administered, followed by alternating treatments of MCT1 and GLUT1 inhibitors for a constant period. In the second scenario, both MCT1 and GLUT1 inhibitors were simultaneously applied for a constant period of time, followed by a therapy-free interval, and this cycle repeated each for 5 days, until the total simulation time reached 30 days. The period was set to 2, 5 and 7,5 days. **(B)**. The control cases. The inhibitors were set to MCT1i]/IC50 = [GLUT1i]/IC50 = 5 and they were continuously applied (Left). No therapy was applied in this case (Right).
