# Supplementary material for: Metabolic symbiosis between oxygenated and hypoxic tumour cells: An agent-based modelling study
Source: PLoS Comput Biol. 2024 Mar 15;20(3):e1011944. doi: 10.1371/journal.pcbi.1011944 (PMC10971686; doi:10.1371/journal.pcbi.1011944)
Supplement: S15 Fig — The model outputs are total number of cells, OXPHOS cell, glycolytic cell, and necrotic cell populations. The outputs have significant correlations with some parameters in a time dependent manner. (DOCX) [file pcbi.1011944.s019.docx]

# **S15 Fig**


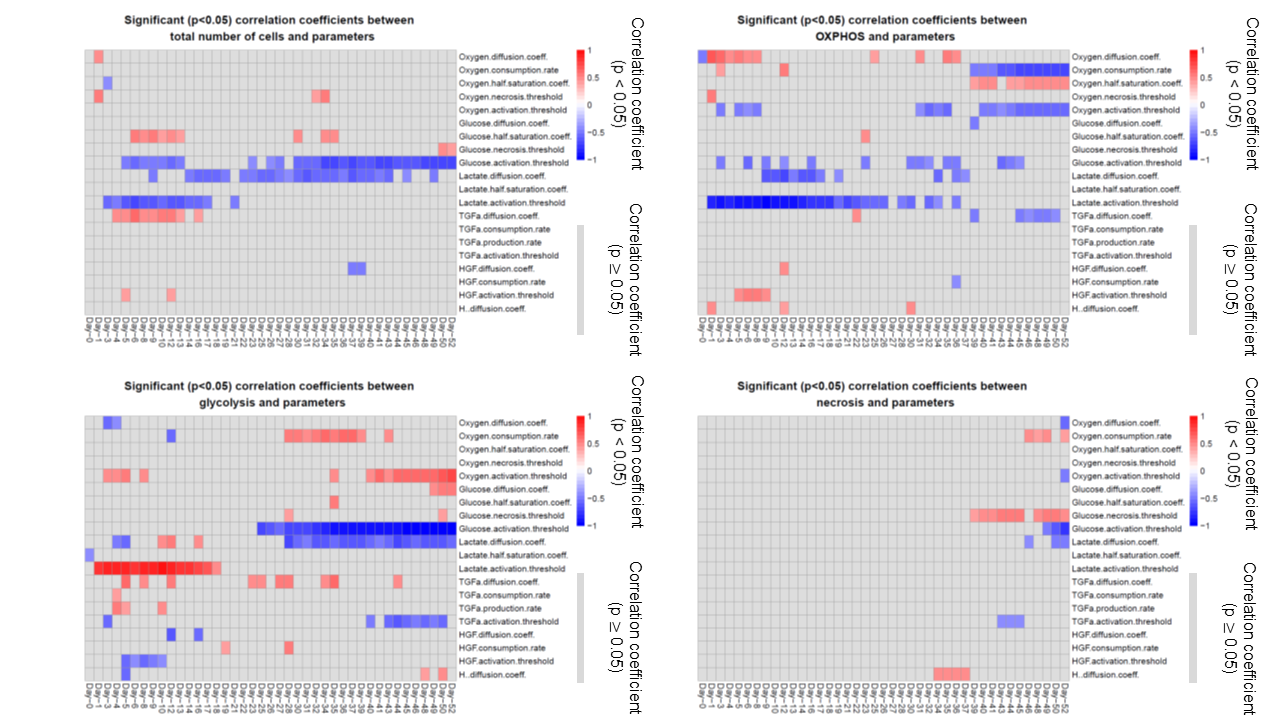


**S15 Fig. Partial correlation coefficients between model parameters and outputs over time:** The model outputs are total number of cells, OXPHOS cell, glycolytic cell, and necrotic cell populations. The outputs have significant correlations with some parameters in a time dependent manner.
