# Supplementary material for: Metabolic symbiosis between oxygenated and hypoxic tumour cells: An agent-based modelling study
Source: PLoS Comput Biol. 2024 Mar 15;20(3):e1011944. doi: 10.1371/journal.pcbi.1011944 (PMC10971686; doi:10.1371/journal.pcbi.1011944)
Supplement: S16 Fig — Glucose activation threshold and oxygen consumption rate have strong positive correlations with metabolic symbiosis while glucose diffusion coefficient and oxygen activation threshold have negative correlations with symbiosis. (DOCX) [file pcbi.1011944.s020.docx]

# **S16 Fig**


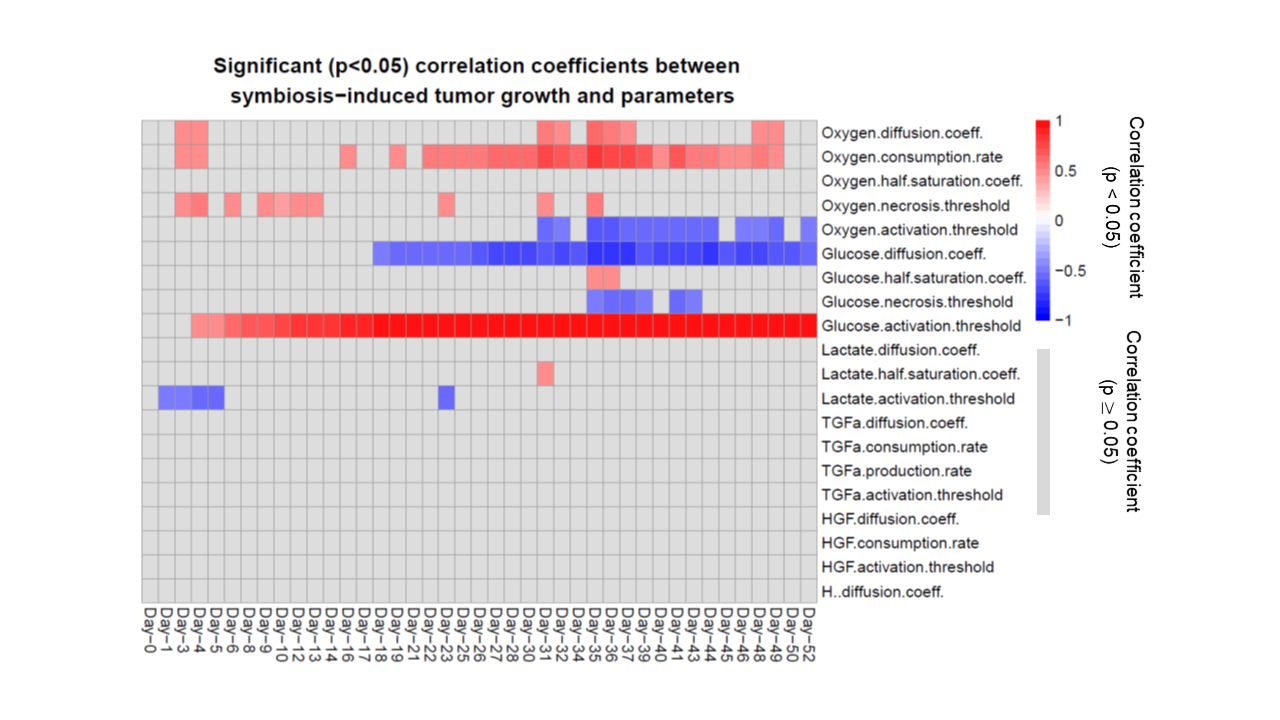


**S16 Fig. Partial correlation coefficients between symbiosis-induced growth increment of tumour and model parameters over time:** Glucose activation threshold and oxygen consumption rate have strong positive correlations with metabolic symbiosis while glucose diffusion coefficient and oxygen activation threshold have negative correlations with symbiosis.
