# Supplementary material for: Metabolic symbiosis between oxygenated and hypoxic tumour cells: An agent-based modelling study
Source: PLoS Comput Biol. 2024 Mar 15;20(3):e1011944. doi: 10.1371/journal.pcbi.1011944 (PMC10971686; doi:10.1371/journal.pcbi.1011944)
Supplement: S17 Fig — (A). Number of active tumour cells with symbiosis (MCT1wt) and without symbiosis (MCT1-) for each perturbed parameter set (PS1-40) and the baseline parameter set (PS-Base). (B). Number of total cells, and mitochondrial and glycolytic ATP producing cells obtained at each parameter set. (DOCX) [file pcbi.1011944.s021.docx]

# **S17 Fig**

**A**

**B**

**
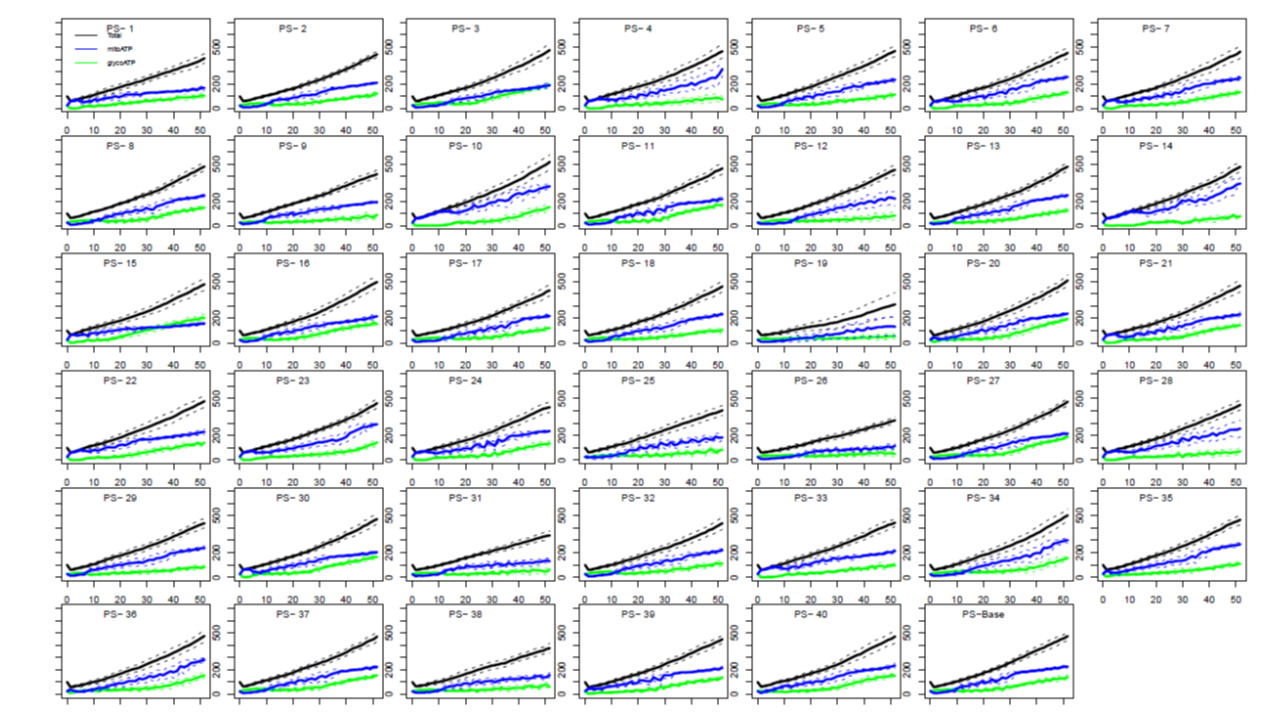
**

**S17 Fig:** **(A)**. Number of active tumour cells with symbiosis (MCT1wt) and without symbiosis (MCT1-) for each perturbed parameter set (PS1-40) and the baseline parameter set (PS-Base). **(B)**. Number of total cells, and mitochondrial and glycolytic ATP producing cells obtained at each parameter set.
